# Clinical Development of Antibacterial Drugs: Recent Progress and Future Outlook

**DOI:** 10.3390/antibiotics15100993

**Published:** 2026-10-07

**Authors:** Dipak Kathayat, Andrea Vidovszky, Yaasmeen Khan, Ashley Denk, Asmita Paudel, Ashutosh Verma, Yosra A. Helmy, Gireesh Rajashekara

**Affiliations:** 1 Richard A. Gillespie College of Veterinary Medicine, Lincoln Memorial University, Harrogate, TN 37752, USAasmita.paudel@lmunet.edu (A.P.); ashutosh.verma@lmunet.edu (A.V.); 2 Department of Veterinary Science, Martin-Gatton College of Agriculture, Food and Environment, University of Kentucky, Lexington, KY 40546, USA; yosra.helmy@uky.edu; 3 Department of Pathobiology, College of Veterinary Medicine, University of Illinois Urbana-Champaign, Urbana, IL 61802, USA; gireesh2@illinois.edu

**Keywords:** antibiotic resistance, antibacterial discovery, antibacterial development, novel antibacterials, innovative antibacterial strategies

## Abstract

The growing burden of antibiotic resistance demands the development of new antibacterials, including those with novel targets and mechanisms of action. This review summarizes key antibacterials that were in clinical development from 2010 to 2026, highlighting their antibacterial classes, mechanisms of action, antibacterial spectrum, clinical applications, development status, and resistance concerns. Antibacterial development during this period encompasses improvements to established antibiotic classes, new β-lactam/β-lactamase inhibitor combinations, siderophore cephalosporins, antibiotic hybrids, antibody-antibiotic conjugates, narrow-spectrum antibacterials, and antibacterials with novel chemical scaffolds, targets, and mechanisms of action. Successful clinical translation, promising emerging antibacterials, and innovations in antibacterial design and delivery are discussed. Lessons from discontinued development programs, including barriers related to safety, efficacy, and emerging resistance, are also highlighted to inform future antibacterial design and clinical trials. Continued investment in antibacterial discovery, early assessment of resistance potential and safety, exploration of nontraditional treatments, application of machine learning and artificial intelligence-guided methods, antibiotic stewardship, and implementation of One Health approaches will be important for addressing the growing challenge of antibiotic resistance.

## 1. Introduction

The growing global burden of antibiotic-resistant bacterial infections, often referred to as a silent pandemic, demands the development of new antibacterial drugs with novel targets or mechanisms of action [1,2,3]. According to an in-depth global analysis of antibiotic resistance, more than 39 million deaths are projected to be attributable to antibiotic-resistant bacterial infections between 2025 and 2050 [1]. *Staphylococcus aureus*, *Acinetobacter baumannii*, *Escherichia coli*, *Klebsiella pneumoniae*, *Streptococcus pneumoniae*, and *Pseudomonas aeruginosa* are the six leading pathogens contributing to the global burden of antibiotic resistance [1]. Reflecting the urgent need to address antibiotic resistance, the 2024 World Health Organization (WHO) Bacterial Priority Pathogens List categorizes antibiotic-resistant bacterial pathogens into critical, high, and medium priority groups to guide research and development of new antibacterial interventions [4]. Among the antibiotic-resistant pathogens prioritized by the WHO are Enterobacterales (including *E*. *coli* and *K*. *pneumoniae*), *A*. *baumannii*, *Mycobacterium tuberculosis*, *S*. *aureus*, *P. aeruginosa*, *Neisseria gonorrhoeae*, *S. pneumoniae*, and *Haemophilus influenzae* [4], underscoring the need for new antibacterials against diverse bacterial pathogens.

The modern antibiotic era began with Alexander Fleming’s discovery of penicillin in 1928 [5]. This breakthrough marked the beginning of a “golden age” of antibiotic discovery from the 1940s to the 1960s, during which most major antibiotic classes were identified [5]. Subsequent decades saw the development of synthetic and semisynthetic antibacterial drugs, while the discovery of fundamentally new antibiotic classes progressively declined [5]. More recently, the growing threat of antibiotic resistance has renewed global interest in antibacterial drugs with novel chemical scaffolds, targets, and mechanisms of action [2,3,6]. This renewed focus has contributed to the development of both approved and investigational antibacterial drugs with activity against priority pathogens and other clinically relevant antibiotic-resistant bacteria [2,3]. Recent approvals, emerging antibacterial candidates, and lessons from discontinued development programs, including barriers related to safety, efficacy, and emerging resistance, provide an opportunity to assess the progress and challenges of antibacterial development and inform future antibacterial design and clinical trials.

**Figure 1:**
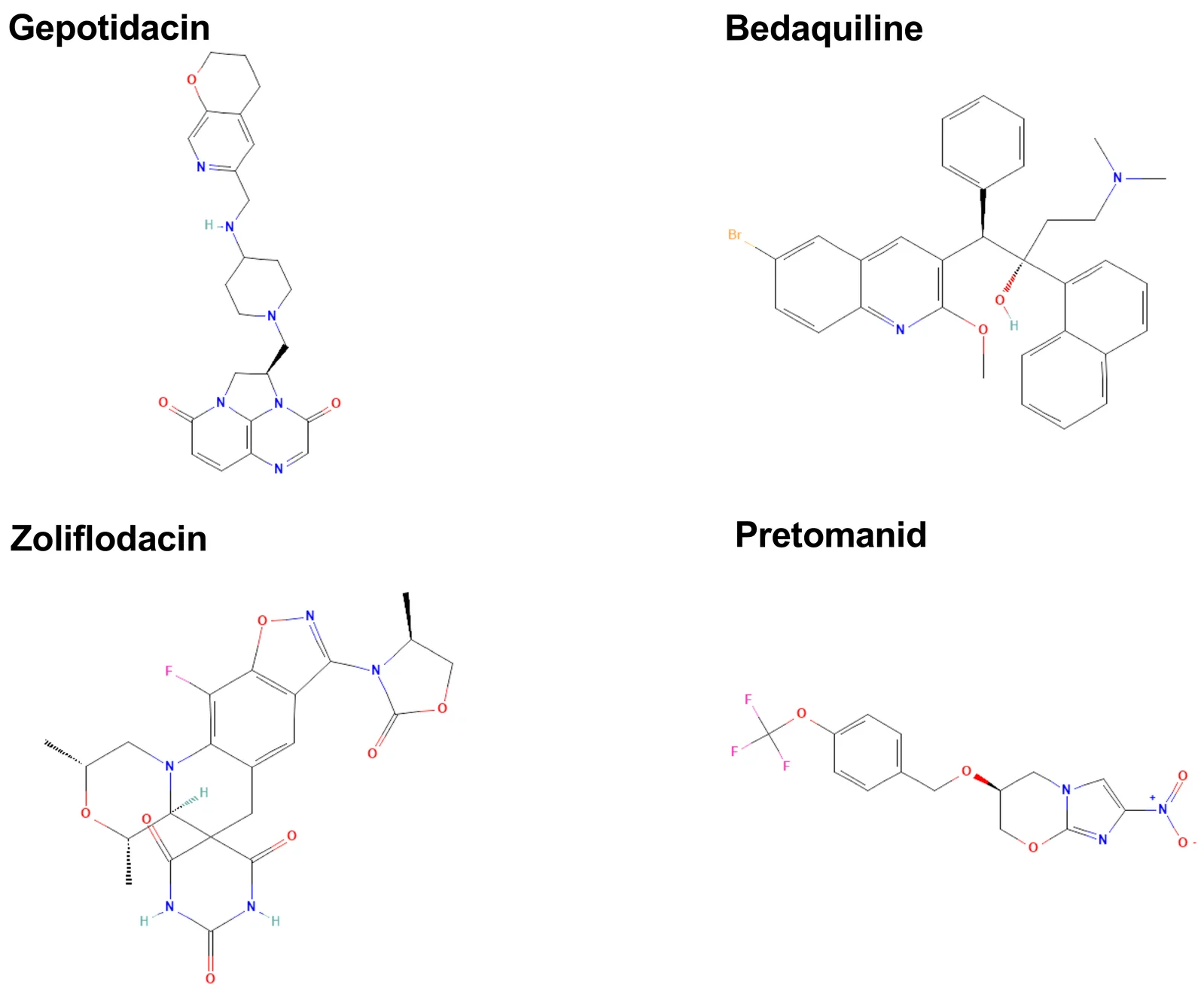
Representative chemical structures of FDA-approved antibacterials with novel chemical scaffolds and distinct mechanisms of action. Gepotidacin is a triazaacenaphthylene derivative that targets bacterial DNA gyrase and topoisomerase IV. Zoliflodacin is a spiropyrimidinetrione that primarily targets bacterial DNA gyrase, with weaker activity against topoisomerase IV. Bedaquiline is a diarylquinoline that targets the c- and ε-subunits of mycobacterial F_1_F_O_-ATP synthase, thereby inhibiting ATP synthesis and disrupting energy production. Pretomanid is a nitroimidazooxazine prodrug that, following activation, inhibits mycolic acid biosynthesis and disrupts mycobacterial respiration.

**Figure 2:**
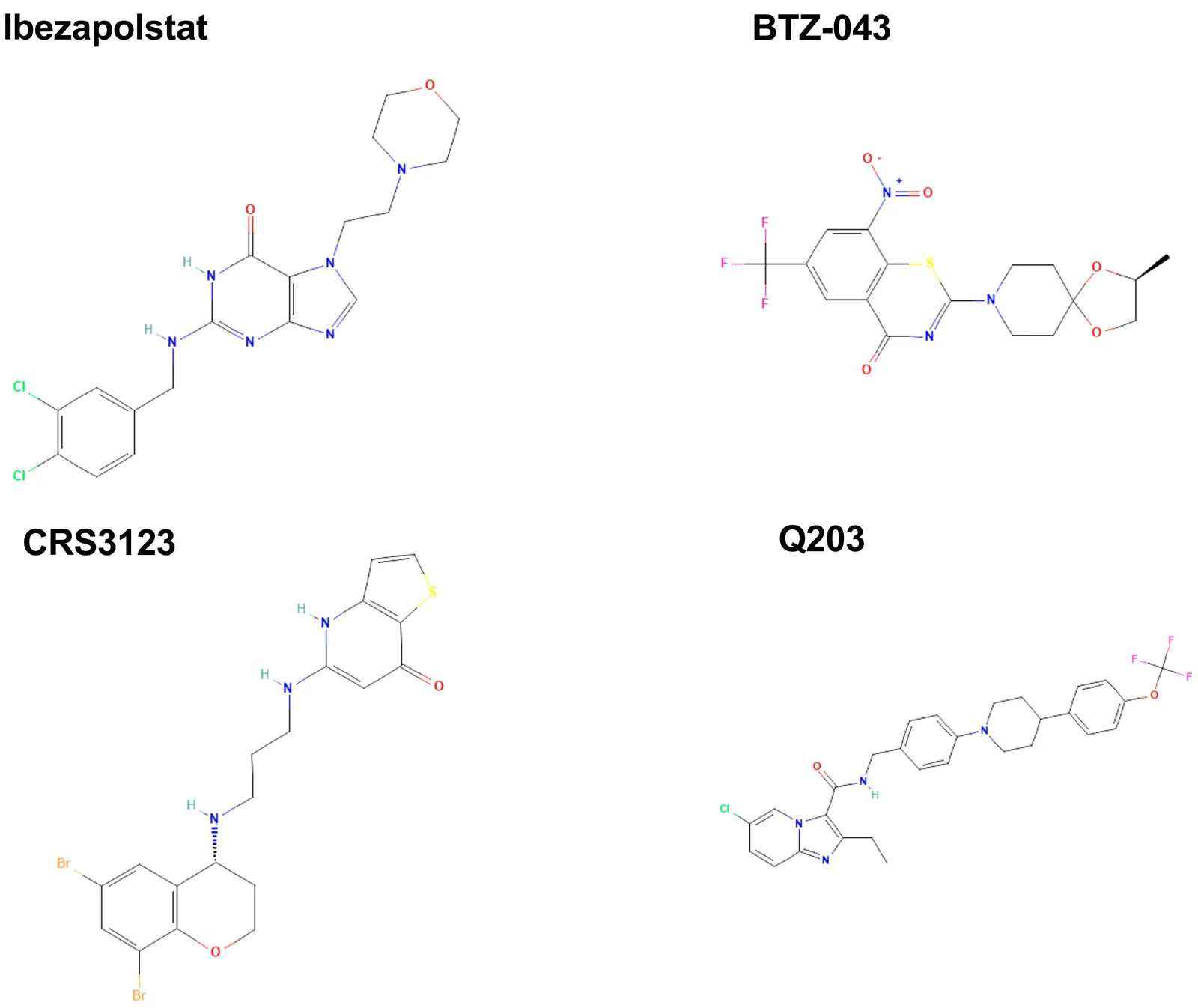
Representative chemical structures of emerging antibacterials with novel chemical scaffolds and distinct mechanisms of action. Ibezapolstat is a synthetic guanine derivative that targets DNA polymerase IIIC, thereby inhibiting DNA replication. CRS3123 is a diaryldiamine that targets bacterial methionyl-tRNA synthetase and inhibits protein synthesis. BTZ-043 is a benzothiazinone derivative that targets decaprenylphosphoryl-β-D-ribose 2’-epimerase 1, thereby inhibiting cell wall arabinan biosynthesis. Q203 is an imidazopyridine amide that targets the QcrB subunit of the cytochrome bcc-aa_3_ oxidase of the mycobacterial electron transport chain.

This review summarizes key antibacterial drugs that were in clinical development from 2010 to 2026 and were identified through searches of the PubMed database. The search was designed to identify antibacterial drugs evaluated in human Phase I-III clinical trials and was supplemented by targeted searches for individual antibacterials. The search strategy combined the major-topic Medical Subject Headings (MeSH) term “Anti-Bacterial Agents” or the title/abstract terms “antibiotic*” and “antibacterial*” with the MeSH term “Bacterial Infections.” These terms were combined with the MeSH term “Drug Development” or the title/abstract terms “investigational,” “development,” “pipeline,” “candidate,” and “first-in-human.” Clinical trials were identified using the publication types “Clinical Trial,” “Clinical Trial, Phase I,” “Clinical Trial, Phase II,” and “Clinical Trial, Phase III,” or the title/abstract terms “phase I,” “phase II,” and “phase III.” The search was restricted to human studies published from 1 January 2010, onward. Antibacterials were included regardless of their subsequent development or approval status and are organized into five major groups: (1) broad-spectrum, (2) Gram-positive infections, (3) Gram-negative infections, (4) mycobacterial infections, and (5) nontraditional treatments. The antibacterial classes, mechanisms of action, antibacterial spectrum, clinical applications, and development status of these antibacterials are highlighted, and associated resistance mechanisms and concerns are discussed. The antibacterial drugs covered in this review are summarized in Table 1, and representative chemical structures of Food and Drug Administration (FDA)-approved and emerging antibacterials with novel chemical scaffolds and distinct mechanisms of action are presented in Figure 1 and Figure 2, respectively. Vaccines, phage-based therapies, monoclonal antibodies, and microbiome-based interventions, including probiotics, live biotherapeutic products, and fecal microbiota transplantation, are beyond the scope of this review.

## 2. Progress in Antibacterial Clinical Development Since 2010

### 2.1. Broad-Spectrum

**Gepotidacin (GSK2140944, BLUJEPA)**. Gepotidacin is an FDA-approved, first-in-class, oral triazaacenaphthylene antibacterial with bactericidal activity against clinically important Gram-positive and Gram-negative pathogens (Figure 1). It inhibits bacterial DNA gyrase and topoisomerase IV through a unique binding mode that is distinct from fluoroquinolones, preventing cross-resistance. Specifically, it binds midway between the scissile DNA bonds and induces single-stranded DNA breaks to arrest the replication fork. This distinct mechanism allows it to retain activity against isolates carrying resistance determinants to established antibiotic classes, including multidrug-resistant (MDR) *N*. *gonorrhoeae*. Gepotidacin is FDA-approved for the treatment of uncomplicated urinary tract infections (uUTIs), including acute uncomplicated cystitis, caused by susceptible *E*. *coli*, *K*. *pneumoniae*, *Citrobacter freundii* complex, *Staphylococcus saprophyticus*, or *Enterococcus faecalis*. It is also FDA-approved for the treatment of uncomplicated urogenital gonorrhea. Clinical efficacy has been demonstrated in both uUTIs and uncomplicated urogenital gonorrhea, and gepotidacin has also been investigated for acute bacterial skin and skin structure infections (ABSSSIs). Its in vitro antibacterial spectrum includes pathogens such as *E*. *coli*, *S*. *aureus*, *S*. *pneumoniae*, and *N*. *gonorrhoeae*, as well as atypical bacteria such as *Chlamydia trachomatis*, *Mycoplasma* spp., and *Ureaplasma* spp. In vitro or preclinical activity has also been reported against selected anaerobic bacteria, *Yersinia pestis*, *Bacillus anthracis*, and drug-resistant mycobacteria, including nontuberculous mycobacteria (NTM) [7,8,9,10,11,12,13,14,15,16,17,18,19,20,21,22,23,24,25].

**Zoliflodacin (ETX0914, AZD0914, Nuzolvence)**. Zoliflodacin is an FDA-approved, first-in-class spiropyrimidinetrione antibacterial that primarily targets DNA gyrase, with weaker activity against topoisomerase IV, through a mechanism distinct from that of fluoroquinolones (Figure 1). It inhibits DNA replication by stabilizing cleaved gyrase-DNA complexes and preventing DNA religation. Consequently, it remains active against MDR *N*. *gonorrhoeae*, including isolates resistant to fluoroquinolones and other established antibiotic classes. It is FDA-approved as a single-dose oral treatment for uncomplicated urogenital gonorrhea caused by *N*. *gonorrhoeae*. Structurally, zoliflodacin contains an *N*-linked oxazolidinone substituent at the 3-position of the benzisoxazole ring fused to a spiropyrimidinetrione core. In vitro activity has also been reported against selected Gram-positive, fastidious Gram-negative, anaerobic, and atypical bacteria, including *Chlamydia* spp., *Helicobacter pylori*, *Mycoplasma* spp., and *Ureaplasma* spp. [26,27,28,29,30,31,32].

**Delafloxacin (ABT-492, Baxdela)**. Delafloxacin is an FDA-approved, potent anionic fluoroquinolone with broad-spectrum activity against clinically important Gram-positive and Gram-negative bacteria, including certain drug-resistant isolates. Its antibacterial spectrum includes *S*. *aureus*, including methicillin-resistant *S*. *aureus* (MRSA), *S*. *pneumoniae*, *E*. *faecalis*, *H*. *influenzae*, *Moraxella catarrhalis*, Enterobacterales such as *E*. *coli* and *K*. *pneumoniae*, *P*. *aeruginosa*, *Legionella* spp., *C*. *trachomatis*, *Chlamydia pneumoniae*, *Mycoplasma* spp., and *Ureaplasma* spp. In vitro or preclinical activity has also been reported against selected anaerobic and microaerophilic bacteria, *Y*. *pestis*, *N*. *gonorrhoeae*, *Neisseria meningitidis*, *H*. *pylori*, *Burkholderia pseudomallei*, *B*. *anthracis*, *Prevotella* spp., *Stenotrophomonas maltophilia*, *Clostridioides difficile*, and selected NTM. Delafloxacin inhibits bacterial DNA gyrase and topoisomerase IV, thereby disrupting DNA replication, transcription, repair, and recombination. Its anionic character enhances bacterial uptake and antibacterial activity under acidic conditions, such as those encountered within inflamed tissues, abscesses, and biofilms, and in vitro antibiofilm activity has been demonstrated. It is FDA-approved for the treatment of ABSSSIs and community-acquired bacterial pneumonia (CABP), while its use for UTIs has also been clinically evaluated [33,34,35,36,37,38,39,40,41,42,43,44,45].

**Ceftobiprole (Ro 63-9141, BAL9141, Zevtera, Mabelio, ceftobiprole medocaril sodium)**. Ceftobiprole is an FDA-approved, broad-spectrum cephalosporin of the pyrrolidinone-3-ylidenemethyl cephem class with bactericidal activity against a wide range of Gram-positive and Gram-negative aerobic bacteria, particularly MRSA, but it has limited activity against many anaerobes. Ceftobiprole inhibits bacterial cell wall synthesis by binding to penicillin-binding proteins (PBPs), including PBP2a in MRSA and PBP2x in penicillin-resistant pneumococci. Its antibacterial spectrum includes drug-resistant staphylococci and streptococci, as well as susceptible *E*. *faecalis*, *H*. *influenzae*, *M*. *catarrhalis*, *E*. *coli*, *P*. *aeruginosa*, *Enterobacter cloacae*, and *K*. *pneumoniae*. In vitro or preclinical activity has also been reported against selected isolates of *A*. *baumannii*, *B*. *pseudomallei*, *N*. *gonorrhoeae*, and *Francisella tularensis*. Its FDA-approved indications include ABSSSIs, *S*. *aureus* bloodstream infections (bacteremia), including cases with right-sided infective endocarditis, and CABP. Ceftobiprole has also been evaluated for hospital-acquired bacterial pneumonia (HABP) and bone and joint infections, but these are not FDA-approved indications [46,47,48,49,50,51,52,53,54].

**Ceftaroline (TAK-599, PPI-0903, Teflaro, ceftaroline fosamil)**. Ceftaroline is an FDA-approved, broad-spectrum cephalosporin with bactericidal activity against MDR Gram-positive and selected Gram-negative pathogens. It inhibits bacterial cell wall synthesis by binding to PBPs, exhibiting high affinity for PBP2a in MRSA and PBP2x in penicillin-resistant *S*. *pneumoniae*. Its antibacterial spectrum includes *S*. *aureus*, including MRSA, *S*. *pneumoniae*, *H*. *influenzae*, *M*. *catarrhalis*, and susceptible Enterobacterales such as *E*. *coli* and *K*. *pneumoniae*. In vitro activity has also been demonstrated against vancomycin-susceptible *E*. *faecalis* and selected Gram-positive anaerobes, although the clinical significance of this activity is less well established. Ceftaroline also exhibits in vitro activity against established *S*. *aureus* biofilms and has been reported to have a relatively limited impact on the human intestinal microbiota. Its FDA-approved indications are ABSSSIs, including cases with concurrent *S*. *aureus* bacteremia, and CABP, including cases with concurrent *S*. *pneumoniae* bacteremia, in both adult and pediatric patients. Off-label or investigational applications include severe MRSA infections such as primary bloodstream infections, infective endocarditis, osteomyelitis and other bone and joint infections, prosthetic joint infections, and meningitis. It has also been evaluated in diabetic foot, postoperative surgical-site, and other complicated wound infections. Mutations in PBP2a and alterations in or increased expression of PBP4 can confer reduced susceptibility or resistance to this antibacterial in *S*. *aureus* [55,56,57,58,59,60,61,62,63,64,65,66].

**GSK2251052 (Epetraborole, AN3365)**. GSK2251052 is an investigational, broad-spectrum, benzoxaborole-class boron-containing leucyl-tRNA synthetase (LeuRS) inhibitor with activity against MDR Gram-negative bacteria, including carbapenem-resistant strains of *E*. *coli*, *K*. *pneumoniae*, and *P*. *aeruginosa*. It inhibits bacterial protein synthesis through an oxaborole tRNA-trapping mechanism that captures the terminal adenosine of tRNA^Leu^ within the LeuRS editing site. Its in vitro antibacterial spectrum also includes anaerobic bacteria, including *Bacteroides fragilis*, *Bacteroides thetaiotaomicron*, and *Clostridium perfringens*, as well as *B*. *pseudomallei*, *N*. *gonorrhoeae*, and *C*. *trachomatis*. Resistance to GSK2251052 emerged rapidly during a Phase II clinical trial for complicated UTIs (cUTIs) owing to mutations in the editing domain of LeuRS, leading to the discontinuation of its original development program for Gram-negative infections. DS86760016 is a newer preclinical LeuRS inhibitor with an improved pharmacokinetic profile and a lower frequency of resistance development than GSK2251052 in animal infection models. Both GSK2251052 and DS86760016 exhibit in vitro, intracellular, and preclinical in vivo activity against *Mycobacterium abscessus*, and DS86760016 also exhibits in vitro activity against other NTM species [67,68,69,70,71,72,73,74,75].

**GSK1322322 (Lanopepden)**. GSK1322322 is an investigational hydroxamic acid-class inhibitor of bacterial peptide deformylase (PDF), an essential enzyme that removes the formyl group from newly synthesized bacterial proteins. Inhibition of PDF disrupts protein maturation and provides activity against MDR respiratory and skin pathogens, including *S*. *aureus*, *S*. *pneumoniae*, *Streptococcus pyogenes*, *H*. *influenzae*, and *M*. *catarrhalis*. It has been clinically evaluated for CABP and ABSSSIs. Further development by GSK was discontinued after potentially reactive metabolites were identified in nonclinical studies, raising concern about possible toxicity that outweighed the anticipated benefit in community-acquired infections [76,77,78].

**Plazomicin (ACHN-90, ACHN-490, Zemdri)**. Plazomicin is an FDA-approved, semisynthetic, next-generation aminoglycoside derived from sisomicin that binds to the 16S rRNA decoding site of the bacterial 30S ribosomal subunit, disrupting translation and inhibiting protein synthesis. Its antibacterial spectrum includes MDR Gram-negative bacilli and selected Gram-positive cocci, particularly Enterobacterales, including isolates of *E*. *coli*, *K*. *pneumoniae*, and *Enterobacter* spp. that produce extended-spectrum β-lactamases (ESBLs), AmpC-β-lactamases, or carbapenemases, as well as *Proteus mirabilis*. In vitro activity has also been reported against selected isolates of *P*. *aeruginosa*, *A*. *baumannii*, MRSA, and colistin-resistant Gram-negative bacteria, although activity against *P*. *aeruginosa* and *A*. *baumannii* is variable. In vitro or preclinical activity has also been demonstrated against *Brucella* spp. and *Y*. *pestis*. Plazomicin is FDA-approved for the treatment of cUTIs, including acute pyelonephritis, caused by susceptible *E*. *coli*, *K*. *pneumoniae*, *P*. *mirabilis*, and *E*. *cloacae*, in patients with limited or no alternative treatment options. Acquired 16S rRNA methyltransferases confer resistance to plazomicin by modifying its ribosomal target. In addition, the recently identified gentamicin-resistance gene *grdA* has been reported to confer high-level plazomicin resistance in *Salmonella enterica*, although its precise biochemical mechanism remains to be determined [79,80,81,82,83,84,85,86,87,88,89,90,91,92].

**Lefamulin (BC-3781, Xenleta, lefamulin acetate)**. Lefamulin is an FDA-approved, semisynthetic pleuromutilin and the first member of this class approved for systemic use in humans. It inhibits bacterial protein synthesis by binding to the peptidyl transferase center of the 50S ribosomal subunit. Lefamulin is active against clinically important respiratory pathogens, including *S*. *aureus*, including MRSA, *S*. *pneumoniae*, *H*. *influenzae*, and *M*. *catarrhalis*, *Legionella pneumophila*, *Mycoplasma pneumoniae*, and *C*. *pneumoniae*. In vitro activity has also been demonstrated against sexually transmitted pathogens, including *Mycoplasma genitalium*, *C*. *trachomatis*, and *N*. *gonorrhoeae*. Its FDA-approved indication is the treatment of CABP, while it has also been clinically evaluated for ABSSSIs [93,94,95,96,97].

**Solithromycin (CEM-101, OP-1068, Solithera)**. Solithromycin is an investigational fluoroketolide that binds to the bacterial 50S ribosomal subunit, thereby inhibiting bacterial protein synthesis. Its in vitro antibacterial spectrum primarily includes Gram-positive bacteria and selected fastidious Gram-negative and atypical pathogens, particularly respiratory tract pathogens, including *S*. *aureus*, *S*. *pneumoniae*, *Streptococcus agalactiae*, *S*. *pyogenes*, *Enterococcus* spp., *H*. *influenzae*, *M*. *catarrhalis*, *Listeria monocytogenes*, *L*. *pneumophila*, and *Bordetella pertussis*, as well as *C*. *trachomatis*, *C*. *pneumoniae*, *N*. *meningitidis*, *N*. *gonorrhoeae*, *Mycoplasma* spp., and *Ureaplasma* spp. It has been clinically evaluated for CABP and uncomplicated urogenital gonorrhea. Although Phase III trials demonstrated noninferiority to moxifloxacin in CABP, solithromycin has not received FDA approval. In a 2016 Complete Response Letter, the FDA requested additional data to characterize the risk of hepatic toxicity and resolution of manufacturing deficiencies [98,99,100,101,102,103,104,105,106,107,108,109,110,111,112].

**NXL-103 (XRP 2868)**. NXL-103 is an investigational, orally administered, semisynthetic combination of the streptogramins linopristin and flopristin, which act at distinct sites on the bacterial 50S ribosomal subunit. Linopristin blocks peptide-chain elongation, whereas flopristin inhibits the peptidyl transferase center. Together, they inhibit bacterial protein synthesis. Its in vitro antibacterial spectrum primarily includes Gram-positive bacteria, including *S*. *aureus*, including MRSA, *S*. *pneumoniae*, *S*. *pyogenes*, *Enterococcus* spp., and Gram-positive anaerobes, as well as selected respiratory Gram-negative bacteria, including *Haemophilus* spp. and *M*. *catarrhalis*. It was clinically evaluated in Phase II trials for CABP and ABSSSIs but has not received regulatory approval [113,114,115,116].

**Iclaprim (RO-48-2622, AR-100)**. Iclaprim is an investigational diaminopyrimidine that selectively inhibits bacterial dihydrofolate reductase, thereby disrupting folate metabolism and DNA synthesis. It is active against drug-resistant Gram-positive pathogens, including *S*. *aureus*, including MRSA, *S*. *pneumoniae*, *S*. *pyogenes*, and *S*. *agalactiae*. In vitro activity has also been reported against selected Gram-negative and atypical bacteria, including *C*. *trachomatis*, *C*. *pneumoniae*, *N*. *gonorrhoeae*, *L*. *pneumophila*, as well as the Gram-positive pathogen *L*. *monocytogenes*. It reached Phase III evaluation for ABSSSIs and was also evaluated for nosocomial pneumonia, but it did not receive FDA approval. A 2009 Complete Response Letter requested additional evidence of efficacy, and a 2019 letter requested additional data on the risk of hepatic toxicity. Its development was subsequently discontinued. At subinhibitory concentrations, iclaprim has also been shown in vitro to suppress the production of selected *S*. *aureus* exotoxins [117,118,119,120,121,122,123,124,125].

**NVC-422 (N,N-dichloro-2,2-dimethyltaurine, Auriclosene)**. NVC-422 is an investigational, broad-spectrum synthetic chloramine and stable analog of *N*-chlorotaurine that inactivates bacterial proteins through oxidation and has rapid microbicidal activity against Gram-positive and Gram-negative bacteria, including *E*. *coli*, *P*. *aeruginosa*, *Staphylococcus* spp., and *Enterococcus* spp. It reached Phase IIb clinical evaluation in topical formulations for impetigo and ocular infections and Phase II evaluation as an irrigant for preventing urinary catheter blockage and encrustation. It also exhibits in vitro antibiofilm activity, and serial-passage studies indicate a low propensity for resistance development. In addition to its direct microbicidal activity, NVC-422 has also been shown in vitro to inactivate virulence factors produced by *S*. *aureus* and *E*. *coli*. However, it has not received regulatory approval [126,127,128,129,130].

**LTX-109 (Voxvoganan, Lytixar)**. LTX-109 is an investigational, broad-spectrum, rapidly acting synthetic antimicrobial peptidomimetic intended for topical use. It exhibits rapid bactericidal activity against *Staphylococcus* spp., including MRSA and isolates resistant to several other antibiotic classes. It binds to negatively charged components of bacterial membranes, causing membrane disruption and cell lysis. Serial-passage studies indicate a low propensity for resistance development. It has been clinically evaluated for the topical treatment of Gram-positive skin infections and nasal decolonization of methicillin-susceptible *S*. *aureus* (MSSA) and MRSA but has not received regulatory approval [131,132].

**Rifasutenizol (TNP-2198)**. Rifasutenizol is an investigational, stable covalent conjugate of rifamycin and nitroimidazole pharmacophores designed as a dual-targeting antibacterial for microaerophilic and anaerobic infections. The rifamycin moiety inhibits bacterial DNA-dependent RNA polymerase, whereas reductive activation of the nitroimidazole moiety generates reactive intermediates that damage bacterial DNA. It exhibits activity against selected microaerophilic and anaerobic pathogens, including *H*. *pylori*, *C*. *difficile*, and *Gardnerella vaginalis*. Clinical development has included bacterial vaginosis but has focused primarily on *H*. *pylori* infection, for which Phase III evaluation has been completed and an application for approval is under review in China [133,134,135].

Collectively, these broad-spectrum antibacterials demonstrate that recent antibacterial development has involved both improvements to established antibiotic classes and the discovery of new chemical scaffolds, targets, and mechanisms of action (Table 1). The successful development of gepotidacin and zoliflodacin, which target bacterial topoisomerases through mechanisms distinct from those of fluoroquinolones, together with delafloxacin, ceftobiprole, ceftaroline, plazomicin, and lefamulin, which build on established classes, illustrates the potential of both approaches. In contrast, the discontinuation of several candidates, including GSK1322322 following safety concerns and GSK2251052 following the emergence of resistance during treatment, underscores the challenges of translating promising antibacterial activity into clinical success and highlights the need to assess safety and resistance potential early in development.

### 2.2. Gram-Positive Infections

**Dalbavancin (BI 397, BI 387, Dalvance)**. Dalbavancin is an FDA-approved, semisynthetic, long-acting lipoglycopeptide that binds the D-Ala-D-Ala terminus of peptidoglycan precursors and inhibits bacterial cell wall synthesis. It is active against Gram-positive pathogens, including MRSA, other susceptible staphylococci, streptococci, vancomycin-susceptible *E*. *faecalis*, and some Gram-positive anaerobes. It is approved for the treatment of ABSSSIs in adult and pediatric patients. Its off-label use and investigation have included bloodstream infections, including catheter-related infections, bone and joint infections, including osteomyelitis and prosthetic joint infections, diabetic foot infections, endocarditis, device-associated infections, and surgical-site infections. Dalbavancin has shown activity against staphylococcal biofilms and has been shown to reduce *S*. *aureus* exotoxin production in vitro. A small study in healthy volunteers found limited effects on intestinal microbiota. In vitro activity has also been reported against *B*. *anthracis*, *C*. *difficile*, *Corynebacterium* spp., *L*. *monocytogenes*, and *Micrococcus* spp. Reduced susceptibility has emerged during treatment, with *walK* mutations identified in some resistant staphylococcal isolates [136,137,138,139,140,141,142,143,144,145,146,147,148,149].

**Oritavancin (LY333328, Orbactiv, Kimysra)**. Oritavancin is an FDA-approved, semisynthetic, long-acting lipoglycopeptide that binds D-Ala-D-Ala terminus of cell wall precursor lipid II to inhibit peptidoglycan synthesis and disrupts bacterial membrane integrity. It is active against drug-resistant Gram-positive bacteria, including MRSA, other susceptible staphylococci, streptococci, and vancomycin-susceptible *E*. *faecalis*. In vitro activity has also been reported against *C*. *difficile*, *B*. *anthracis*, vancomycin-resistant enterococci (VRE), and some Gram-positive anaerobes. It is approved for the treatment of ABSSSIs in adults. Its off-label use and investigation have included bloodstream infections, including catheter-related infections, infective endocarditis, surgical-site infections, pneumonia, and bone and joint infections, including osteomyelitis and prosthetic joint infections [150,151,152,153,154,155,156,157].

**TD-1792 (Cefilavancin)**. TD-1792 is an investigational, first-in-class, multivalent glycopeptide-cephalosporin heterodimer whose components are proposed to bind peptidoglycan precursors and PBPs, respectively, with potent activity against Gram-positive bacteria, including MRSA. In vitro activity has also been reported against anaerobic Gram-positive bacteria and *Corynebacterium* spp. Following evaluation for complicated skin and skin structure infections (cSSSIs) in a Phase II trial, its clinical evaluation advanced into Phase III trials, although its subsequent development status is unclear [158,159,160,161,162].

**Tedizolid phosphate (TR-701, DA-7218, Sivextro)**. Tedizolid phosphate is an FDA-approved, second-generation oxazolidinone prodrug whose active moiety, tedizolid (DA-7157, torezolid, TR-700), binds to the peptidyl transferase center of the bacterial 50S ribosomal subunit and inhibits protein synthesis. Tedizolid is active against Gram-positive pathogens, including MSSA, MRSA, isolates of *S*. *pneumoniae* that are penicillin-susceptible, penicillin-resistant, or MDR, and susceptible enterococci. In vitro activity has also been demonstrated against VRE, certain linezolid-resistant staphylococci and enterococci, *M*. *tuberculosis*, some NTM, *Nocardia brasiliensis*, and the anaerobe *B*. *fragilis*. Tedizolid phosphate is approved for the treatment of ABSSSIs in adult and pediatric patients. It has also been evaluated in a Phase III trial for HABP/ventilator-associated bacterial pneumonia (VABP) and investigated for bone and joint infections [163,164,165,166,167,168,169].

**Cadazolid**. Cadazolid is an investigational, narrow-spectrum, bactericidal fluoroquinolone-oxazolidinone hybrid developed for the treatment of *C*. *difficile* infection. It primarily inhibits bacterial protein synthesis by binding near the peptidyl transferase center of the ribosome and has weaker inhibitory effects on DNA synthesis, with a low potential for spontaneous resistance development observed in vitro. Its antibacterial spectrum includes epidemic *C*. *difficile* strains and isolates resistant to linezolid or moxifloxacin, and it also inhibits toxin production and spore formation in vitro. It is minimally absorbed and has shown a limited impact on the gut microflora in experimental models. In Phase III trials, cadazolid did not meet the clinical-cure noninferiority endpoint in one of two studies, and its development was discontinued [170,171,172,173,174].

**Fusidic acid (CEM-102)**. Fusidic acid is a long-used steroidal antistaphylococcal antibacterial active against staphylococci, including MRSA. It inhibits bacterial protein synthesis through interaction with elongation factor G. In vitro activity has also been demonstrated against *N*. *gonorrhoeae* and *C*. *trachomatis*. It has been clinically investigated for ABSSSIs in a Phase III trial and for prosthetic joint infections in a Phase II combination trial with rifampicin that was prematurely terminated because of a drug–drug interaction [175,176,177,178,179].

**CRS3123 (REP3123)**. CRS3123 is a novel diaryldiamine that targets bacterial methionyl-tRNA synthetase and inhibits protein synthesis in Gram-positive bacteria (Figure 2). It is highly active against *C*. *difficile*, including resistant strains, and aerobic Gram-positive cocci, but has limited activity against many commensal intestinal anaerobes and most Gram-negative bacteria. It also inhibits spore and toxin production in experimental studies, and its narrow spectrum is associated with limited disruption of the normal gut microbiota. In a small Phase II trial for *C*. *difficile* infection, CRS3123 showed clinical activity and numerically lower recurrence rates than vancomycin [180,181,182,183,184,185].

**Ibezapolstat (ACX-362E, MorE-DCBG)**. Ibezapolstat is a synthetic guanine derivative, N^2^-(3,4-dichlorobenzyl)-7-(2-[1-morpholinyl]ethyl)guanine, and a first-in-class DNA polymerase IIIC inhibitor that inhibits DNA replication in *C*. *difficile* (Figure 2). As a Gram-positive-selective antibacterial, it has shown more favorable effects on gut microbiome composition compared with vancomycin, including the preservation or regrowth of certain commensal bacteria. It has also shown clinical activity against *C*. *difficile* infection in Phase IIa and IIb studies, with preliminary findings suggesting a lower risk of recurrence, and exhibits in vitro activity against MDR strains, with Phase III evaluation planned [186,187,188,189,190,191].

**DS-2969b**. DS-2969b is a novel DNA gyrase subunit B inhibitor that blocks the supercoiling activity of *C*. *difficile* DNA gyrase and inhibits DNA replication. Oral DS-2969b was evaluated in Phase I studies in healthy volunteers as a potential treatment for *C*. *difficile* infection, though further clinical development has since been discontinued for reasons that have not been publicly reported. Its intravenous prodrug, DS11960558, was evaluated in preclinical studies. In vitro activity has also been demonstrated against Gram-positive aerobes, including MRSA [192,193,194].

**Ridinilazole (SMT19969)**. Ridinilazole is a novel, narrow-spectrum, nonabsorbable bis-benzimidazole with potent activity against *C*. *difficile* but limited activity against many other Gram-positive and Gram-negative aerobic and anaerobic intestinal bacteria. It binds to the DNA minor groove, resulting in transcriptional dysregulation and bacterial cell death. Experimental studies have also shown impaired cell division and reduced toxin production. It has demonstrated clinical activity against *C*. *difficile* infection while preserving gut microbiome diversity better than vancomycin. In a Phase III trial, recurrence was lower with ridinilazole, although it did not meet the primary endpoint for superiority in sustained clinical response. Further clinical development is planned [195,196,197,198,199,200,201].

**Fidaxomicin (OPT-80, PAR-101, Difimicin, Lipiarmycin A3, Dificid, Tiacumicin B, Clostomicin B1)**. Fidaxomicin is a narrow-spectrum, FDA-approved, macrocyclic antibiotic that binds to bacterial RNA polymerase and inhibits transcription. Its antibacterial activity is primarily directed against the Gram-positive pathogen *C*. *difficile*, with little or no activity against Gram-negative bacteria such as *Bacteroides* spp. It is approved for the treatment of *C*. *difficile*-associated diarrhea in adult and pediatric patients. It largely preserves the normal gut microbiota compared with vancomycin, helping maintain microbiome diversity and colonization resistance. It also inhibits sporulation and toxin production and shows activity against established *C*. *difficile* biofilms in experimental studies. Clinical trials have found lower recurrence rates with fidaxomicin than with standard vancomycin treatment. Mutations in *rpoB* or *rpoC*, which encode the β and β’ subunits, respectively, of bacterial RNA polymerase, can reduce susceptibility to fidaxomicin [202,203,204,205,206,207,208,209,210,211,212,213,214,215,216].

**Surotomycin (CB-183,315, MK-4261)**. Surotomycin is an investigational, membrane-active, bactericidal cyclic lipopeptide that disrupts bacterial membrane potential, with potent activity against *C*. *difficile*, including antibiotic-resistant strains. It is a structural analog of daptomycin and was evaluated for *C*. *difficile* infection in two Phase III trials, meeting the noninferiority criterion against vancomycin in one trial but not the other. Due to these discordant results, its development has been discontinued. Its in vitro antibacterial spectrum also includes a broad range of Gram-positive aerobic and anaerobic pathogens, including MRSA and vancomycin-susceptible and vancomycin-resistant strains of *E*. *faecalis* and *Enterococcus faecium*. It exhibits a low frequency of resistance development in vitro and has been shown to reduce toxin production under some experimental conditions. Its limited activity against Gram-negative anaerobes and Enterobacteriaceae spares some components of the gut microbiota, although effects on Gram-positive intestinal bacteria have been observed [217,218,219,220,221,222,223,224,225].

**XF-73 (Exeporfinium chloride)**. XF-73 is an investigational, first-in-class, dicationic porphyrin derivative that disrupts bacterial membrane integrity and has rapid bactericidal activity against staphylococci. Its in vitro antibacterial spectrum includes antibiotic-susceptible and antibiotic-resistant Gram-positive bacteria. A Phase II clinical study found that XF-73 reduced *S*. *aureus* nasal carriage. It is also active against slow-growing, non-dividing, and biofilm-associated *S*. *aureus* cells in vitro [226,227,228,229,230].

**Afabicin (Debio 1450, AFN-1720)**. Afabicin is a novel, first-in-class, narrow-spectrum antistaphylococcal antibacterial under development. As a prodrug, afabicin is converted in vivo to its active form, afabicin desphosphono (Debio 1452, AFN-1252), which inhibits enoyl-acyl carrier protein reductase, an enzyme critical for fatty acid biosynthesis in staphylococci. Its antibacterial spectrum is primarily restricted to *S*. *aureus*, including MSSA and MRSA, as well as coagulase-negative staphylococci, with limited activity against other Gram-positive or Gram-negative species. It has been clinically evaluated in Phase II trials for ABSSSIs and bone and joint infections. Oral afabicin has shown limited disruption of the gut microbiota in healthy volunteers [231,232,233,234].

**DSTA4637S (RG7861)**. DSTA4637S is a novel antibody-antibiotic conjugate designed to eliminate *S*. *aureus* within host cells, where it can evade standard-of-care antibiotics. The conjugate consists of an anti-*S*. *aureus* Thiomab human immunoglobulin G1 monoclonal antibody linked to a novel rifamycin-class antibiotic, 4-dimethylaminopiperidino-hydroxybenzoxazino rifamycin (dmDNA31), through a protease-cleavable linker. The antibody binds *S*. *aureus*, and following uptake into host cells, intracellular proteases cleave the linker to release dmDNA31, which inhibits bacterial RNA polymerase. DSTA4637S has been evaluated in a Phase I study in healthy volunteers and a Phase Ib study in patients with *S*. *aureus* bacteremia receiving standard-of-care antibiotics [235,236].

**α-Viniferin**. α-Viniferin is a bioactive phytochemical compound with in vitro activity against drug-susceptible and drug-resistant strains of *M*. *tuberculosis* and staphylococci, including MSSA, MRSA, and methicillin-resistant *Staphylococcus epidermidis*. In a small clinical study, topical α-viniferin reduced nasal *S*. *aureus* carriage without significantly reducing the other nasal bacteria measured. Its precise antibacterial target has not been established [237,238,239].

Overall, the development of Gram-positive antibacterials demonstrates the continued advancement of established antibiotic classes alongside a growing emphasis on narrow-spectrum antibacterials with novel targets and mechanisms of action (Table 1). The successful development of dalbavancin, oritavancin, tedizolid, and fidaxomicin, together with the investigation of CRS3123, ibezapolstat, and afabicin, illustrates the potential of both established and targeted approaches against Gram-positive pathogens. In particular, narrow-spectrum antibacterials directed against *C*. *difficile* and *S*. *aureus* reflect increasing interest in pathogen-selective activity with the potential to limit disruption of the normal microbiota. Other approaches, including membrane-active antibacterials and an antibody-antibiotic conjugate designed to reach intracellular *S*. *aureus*, broaden the strategies being explored. However, mixed outcomes in late-stage trials for several candidates underscore the need to establish clear clinical benefits alongside antibacterial activity.

### 2.3. Gram-Negative Infections

**HRS-8427**. HRS-8427 is a novel parenteral siderophore cephalosporin currently in Phase II/III clinical development for the treatment of aerobic Gram-negative bacterial infections, including cUTIs and HABP/VABP. It exploits bacterial iron-uptake systems to enter the periplasm, where it binds PBPs and inhibits cell wall biosynthesis. HRS-8427 has demonstrated potent in vitro activity against MDR Gram-negative bacteria, including carbapenem-resistant Enterobacterales (CRE), *P*. *aeruginosa*, and *A*. *baumannii*, as well as favorable pharmacokinetic and safety profiles in early clinical studies [240,241,242].

**Cefiderocol (S-649266, Fetroja)**. Cefiderocol is an FDA-approved siderophore cephalosporin that forms a ferric iron-chelating complex, exploiting bacterial iron transport systems to enter the periplasm of Gram-negative bacteria. After entry, it binds primarily to PBP3 and inhibits peptidoglycan synthesis. Cefiderocol exhibits potent in vitro activity against MDR Gram-negative pathogens, including ESBL-producing, carbapenemase-producing, and carbapenem-resistant isolates of *E*. *coli*, *K*. *pneumoniae*, *Enterobacter* spp., *P*. *aeruginosa*, *A*. *baumannii*, *S*. *maltophilia*, the *Burkholderia cepacia* complex, *B*. *pseudomallei*, and *Achromobacter* spp., including isolates harboring diverse serine β-lactamases and metallo-β-lactamases. FDA-approved indications include cUTIs, including acute pyelonephritis, and HABP/VABP in adults with infections caused by susceptible Gram-negative bacteria. Cefiderocol has also been evaluated for complicated intra-abdominal infections (cIAIs) and bloodstream infections, while its use for osteomyelitis, prosthetic joint infections, meningitis, endocarditis, and ventriculitis has been described primarily in case reports and observational studies. It also exhibits in vitro activity against biofilm-associated Gram-negative bacteria. Resistance can result from β-lactamase production, mutations affecting PBPs, and alterations in TonB-dependent siderophore receptors, including PiuA, CirA, and FptA in different bacterial species, that reduce drug uptake [243,244,245,246,247,248,249,250,251,252,253,254,255,256].

**Benapenem**. Benapenem is a novel investigational intravenous carbapenem with potent in vitro activity against Enterobacterales, including ESBL-producing *E*. *coli*, *K*. *pneumoniae*, and *E*. *cloacae* isolates. It binds PBPs and inhibits bacterial cell wall synthesis. Benapenem has a prolonged elimination half-life that supports once-daily administration and has been evaluated in a combined Phase II/III clinical trial for cUTIs, including acute pyelonephritis [257].

**Cefepime-taniborbactam**. Cefepime-taniborbactam is an investigational broad-spectrum antibacterial combination consisting of the fourth-generation cephalosporin cefepime and the novel bicyclic boronate β-lactamase inhibitor taniborbactam. It exhibits potent in vitro activity against MDR Gram-negative bacteria, including ESBL-producing strains, CRE, and MDR *P*. *aeruginosa* isolates. Activity has also been reported against selected isolates of *S*. *maltophilia*, the *B*. *cepacia* complex, and *B*. *pseudomallei*. Mechanistically, cefepime binds PBPs and inhibits cell wall synthesis, whereas taniborbactam inhibits serine-β-lactamases from Ambler classes A, C, and D, as well as several class B metallo-β-lactamases, including many NDM- and VIM-type enzymes, thereby protecting cefepime from hydrolysis. It also inhibits cefepime-hydrolyzing PDC variants, including PDC-88, thereby restoring cefepime activity. However, resistance has been associated with IMP-type enzymes and certain NDM and VIM variants, including NDM-9. Clinically, this combination demonstrated superiority over meropenem in a Phase III clinical trial for cUTIs, including acute pyelonephritis. Although the FDA issued a Complete Response Letter requesting additional chemistry, manufacturing, and controls information, it identified no deficiencies related to clinical safety or efficacy, and development remains ongoing [258,259,260,261,262,263,264,265,266,267,268,269].

**Ceftazidime-avibactam (Avycaz)**. Ceftazidime-avibactam is an FDA-approved antibacterial combination consisting of ceftazidime, a cephalosporin that binds PBPs and inhibits cell wall synthesis, and avibactam, a non-β-lactam β-lactamase inhibitor (formerly designated AVE1330A and NXL104). Avibactam belongs to the diazabicyclooctane class and inhibits Ambler classes A and C β-lactamases and certain class D β-lactamases, including KPC and OXA-48-like carbapenemases, but lacks activity against class B metallo-β-lactamases. This combination is active against MDR Gram-negative pathogens, particularly Enterobacterales, such as *E*. *coli* and *K*. *pneumoniae*, including ESBL- and serine-carbapenemase-producing strains. Clinically relevant activity has also been demonstrated against *P*. *aeruginosa* and selected isolates of the *B*. *cepacia* complex. Approved clinical indications include cUTIs, including acute pyelonephritis, cIAIs, when administered in combination with metronidazole, and HABP/VABP. Off-label clinical use has also been documented for bloodstream infections, bone and joint infections, endocarditis, and central nervous system (CNS) infections caused by susceptible Gram-negative bacteria. However, emerging resistance poses a clinical challenge. Mutations and structural alterations in KPC enzymes, particularly substitutions or deletions within the Ω-loop, such as the D179Y substitution, can emerge following treatment and can confer resistance to ceftazidime-avibactam. Altered membrane permeability, upregulated efflux pumps, and increased expression or structural modification of other β-lactamases contribute to treatment failure [270,271,272,273,274,275,276,277,278,279,280,281].

**Ceftolozane-tazobactam (Zerbaxa)**. Ceftolozane-tazobactam is an FDA-approved antibacterial combination consisting of ceftolozane, an oxyimino-aminothiazolyl cephalosporin that binds PBPs, particularly PBP3, to inhibit cell wall synthesis, and tazobactam, a β-lactamase inhibitor that protects ceftolozane from hydrolysis by susceptible β-lactamases. The combination has potent antipseudomonal activity against MDR and selected extensively drug-resistant (XDR) *P*. *aeruginosa* isolates. Its antibacterial spectrum includes *P*. *aeruginosa*, with in vitro activity against biofilm-associated bacteria and isolates recovered from patients with cystic fibrosis, as well as Enterobacterales such as *E*. *coli*, *K*. *pneumoniae*, and *E*. *cloacae* complex isolates, including many ESBL-producing isolates. Variable in vitro activity has also been reported against selected isolates of the *B*. *cepacia* complex, *B*. *pseudomallei*, *H*. *influenzae*, and *Salmonella* spp., with good activity against many *B*. *fragilis* isolates but variable or limited activity against other *Bacteroides* species. Approved indications in adult and pediatric patients include cUTIs, including pyelonephritis, cIAIs, when administered in combination with metronidazole, and HABP/VABP. Reported off-label uses included bloodstream infections, including those associated with sepsis, pulmonary infections outside the approved HABP/VABP protocol, bone and joint infections, including osteomyelitis, CNS infections, including meningitis, endocarditis, and otitis. Resistance is associated with the overexpression or structural modification of PDC, *ampD* mutations that promote PDC derepression, OXA-2 variants carrying a D149 duplication, such as OXA-539, the acquisition of horizontally transferred metallo-β-lactamases, OXA-type β-lactamases, and VEB β-lactamases, as well as mutations that promote the overexpression of the MexCD-OprJ efflux pump [282,283,284,285,286,287,288,289,290,291,292,293,294,295,296,297,298].

**Sulbactam-durlobactam (Xacduro)**. Sulbactam-durlobactam is an FDA-approved antibacterial combination consisting of sulbactam and durlobactam (formerly designated ETX2514), a novel broad-spectrum diazabicyclooctane serine β-lactamase inhibitor. Sulbactam exerts intrinsic antibacterial activity against the *A*. *baumannii*-*calcoaceticus* complex by binding PBPs and inhibiting cell wall synthesis, whereas durlobactam inhibits class A, C, and D β-lactamases that would otherwise hydrolyze sulbactam. This combination is active against MDR and carbapenem-resistant isolates of the *A*. *baumannii*-*calcoaceticus* complex. Its FDA-approved indication is the treatment of HABP/VABP caused by susceptible isolates of this complex in adults with limited or no alternative treatment options. Preclinical activity has also been demonstrated against pathogenic *Burkholderia* species [299,300,301,302,303,304].

Together, the development of Gram-negative antibacterials has focused on complementary strategies, including combinations of β-lactams and β-lactamase inhibitors that counter diverse serine β-lactamases and, in some cases, metallo-β-lactamases, siderophore cephalosporins that exploit bacterial iron-uptake systems to overcome outer-membrane permeability barriers, pathogen-directed combinations such as sulbactam-durlobactam, and a carbapenem with improved pharmacokinetic properties (Table 1). The FDA approvals of cefiderocol, ceftazidime-avibactam, ceftolozane-tazobactam, and sulbactam-durlobactam, together with the clinical development of HRS-8427, cefepime-taniborbactam, and benapenem, support the potential of these approaches to expand treatment options for infections caused by MDR Gram-negative pathogens. However, resistance mediated by β-lactamase acquisition or modification, altered PBPs, reduced siderophore-mediated uptake, decreased membrane permeability, and increased efflux emphasizes the continued need to anticipate and monitor resistance during antibacterial development and clinical use.

### 2.4. Mycobacterial Infections

**Bedaquiline (R207910, TMC207, Sirturo)**. Bedaquiline is a first-in-class, FDA-approved diarylquinoline with potent bactericidal activity (Figure 1). While it possesses in vitro activity against drug-susceptible and drug-resistant strains of *M*. *tuberculosis*, it is FDA-approved as part of combination therapy for pulmonary tuberculosis (TB) caused by *M*. *tuberculosis* resistant to at least rifampicin and isoniazid. It also exhibits in vitro antimycobacterial activity against other mycobacterial species, including *Mycobacterium leprae*, *Mycobacterium ulcerans*, and members of the *Mycobacterium avium*, *M*. *abscessus*, and *Mycobacterium fortuitum* complexes. Bedaquiline interacts with the c-ring and ε-subunit of mycobacterial F_1_F_O_-ATP synthase, thereby inhibiting ATP synthesis and disrupting cellular energy metabolism. Mutations in *atpE* and *rv0678* can confer resistance to bedaquiline, with *rv0678* mutations causing derepression of the MmpS5-MmpL5 efflux pump. While bedaquiline is highly effective, it carries clinical liabilities, most notably tissue accumulation and a risk of QT interval prolongation via hERG channel inhibition. TBAJ-876 (sorfequiline) and TBAJ-587 are next-generation diarylquinoline analogs designed with reduced lipophilicity to reduce tissue accumulation and minimize hERG interaction. Preclinical studies suggest greater antimycobacterial activity and a lower potential for QT prolongation than bedaquiline, while early clinical studies have characterized the analogs’ pharmacokinetics and safety. In preclinical and clinical pharmacokinetic studies, bedaquiline has also demonstrated CNS penetration and accumulation, suggesting its potential to target *M*. *tuberculosis* reservoirs in the CNS. The BPaL regimen, comprising bedaquiline, pretomanid, and linezolid, has demonstrated profound clinical efficacy against highly drug-resistant pulmonary TB, including XDR-TB and treatment-intolerant or nonresponsive MDR-TB [305,306,307,308,309,310,311,312,313,314,315,316,317,318,319,320,321,322,323,324,325,326,327,328,329,330,331,332].

**Sudapyridine (WX-081)**. Sudapyridine is an investigational structural analog of bedaquiline with potent activity against drug-susceptible and drug-resistant *M*. *tuberculosis*, including MDR strains. Preclinical and clinical studies indicate a reduced potential for QT interval prolongation and improved pharmacokinetic properties compared with bedaquiline. Sudapyridine has advanced it into Phase III clinical trials for rifampicin-resistant (RR) pulmonary TB. Sudapyridine has also demonstrated in vitro, intracellular, and in vivo activity against NTM, including *M*. *abscessus*, *M*. *avium*, and *Mycobacterium chelonae*. Similar to bedaquiline, sudapyridine inhibits mycobacterial ATP synthase by targeting the c-ring and disrupts the proton motive force. It has also been reported to enhance innate immune responses via NF-κB and MAPK pathway activation in macrophage-based studies [333,334,335,336,337,338,339,340,341].

**Q203 (Telacebec)**. Q203 is an investigational imidazopyridine amide that targets the QcrB subunit of the mycobacterial cytochrome bcc-aa_3_ oxidase complex, thereby disrupting the electron transport chain and cellular respiration (Figure 2). It exhibits potent in vitro activity against drug-susceptible and drug-resistant *M*. *tuberculosis*, including MDR and XDR strains. It was evaluated clinically in patients with drug-susceptible pulmonary TB. Beyond TB, it has demonstrated exceptional clinical development potential against *M*. *ulcerans* and *M*. *leprae*, successfully advancing into Phase II clinical trials for Buruli ulcer and formal global development partnerships for leprosy [342,343,344,345,346].

**BTZ-043**. BTZ-043 is an investigational benzothiazinone derivative that covalently inhibits decaprenylphosphoryl-β-D-ribose 2′-epimerase 1 (DprE1), thereby blocking the biosynthesis of decaprenylphosphoryl-β-D-arabinose and, consequently, cell wall arabinan in *M*. *tuberculosis* (Figure 2). Having advanced into Phase IIb/c combination clinical trials for drug-susceptible pulmonary TB, BTZ-043 has also demonstrated preclinical activity against other Corynebacterineae, including *Corynebacterium* spp. and *Nocardia* spp. [347,348,349,350,351,352,353,354].

**PBTZ169 (Macozinone)**. PBTZ169 is an investigational piperazine-containing benzothiazinone derivative that covalently inhibits the DprE1 enzyme of *M*. *tuberculosis* by binding to the C387 residue inside the active pocket, thereby blocking cell wall arabinan biosynthesis. Following the completion of safety, tolerability, and pharmacokinetic profiling in healthy volunteers, it has advanced into Phase II clinical evaluation as an early bactericidal candidate for shortened treatment regimens in patients with drug-susceptible pulmonary TB. Additionally, recent macrophage studies demonstrate that PBTZ169 enhances host innate immune responses by upregulating the NF-κB and MAPK signaling pathways [355,356,357].

**Quabodepistat (OPC-167832)**. Quabodepistat is an investigational carbostyril derivative that inhibits DprE1, thereby blocking the biosynthesis of decaprenylphosphoryl-β-D-arabinose and, consequently, cell wall arabinan in *M*. *tuberculosis*. Following clinical evaluation for drug-susceptible pulmonary TB, it advanced into Phase III trials for MDR pulmonary TB. It also demonstrates in vitro bactericidal activity against *M*. *abscessus*. However, due to significantly lower relative potency against this NTM species, therapeutic efficacy was not observed in a mouse infection model, making it an unlikely candidate for NTM repurposing [358,359,360].

**SQ109**. SQ109 is an investigational *N*-geranyl-*N*′-(2-adamantyl)ethane-1,2-diamine structurally related to ethambutol, with activity against *M*. *tuberculosis*. Following early safety studies, SQ109 was evaluated in patients with drug-susceptible pulmonary TB and in a multicenter Phase IIb/III trial as part of a treatment regimen for MDR-TB. It acts by inhibiting the trehalose monomycolate transporter MmpL3 and disrupting the proton motive force, which impairs mycolic acid incorporation into the mycobacterial cell wall. In vitro bactericidal activity has also been reported against both drug-susceptible and drug-resistant strains of *H*. *pylori* [361,362,363,364,365,366].

**Posizolid (AZD-5847, AZD-2563)**. Posizolid is an oxazolidinone that binds to the 50S ribosomal subunit and prevents formation of the translation-initiation complex, thereby inhibiting bacterial protein synthesis. It exhibits in vitro activity against a broad range of Gram-positive bacteria, particularly *Staphylococcus* spp., *Streptococcus* spp., and *Enterococcus* spp., including strains resistant to other antibiotic classes. Its in vitro antibacterial spectrum also includes *Corynebacterium* spp., *Listeria* spp., and *Bacillus* spp., Gram-positive anaerobes, and drug-susceptible and drug-resistant strains of *M*. *tuberculosis*. However, due to a high incidence of adverse hematologic and hepatic effects alongside modest effects compared to linezolid in a Phase II trial involving patients with drug-susceptible pulmonary TB, further development of posizolid has been discontinued [367,368,369,370,371].

**Contezolid (MRX-I)**. Contezolid is a potent oxazolidinone with activity against Gram-positive pathogens, including MRSA, VRE, and other antibiotic-resistant strains. It has also demonstrated in vitro and preclinical activity against both drug-susceptible and drug-resistant strains of *M*. *tuberculosis*, as well as *M*. *abscessus*. Compared with linezolid, contezolid has a more favorable hematologic safety profile, including significantly lower incidences of leukopenia and thrombocytopenia in a Phase III trial. Because contezolid and linezolid both bind the peptidyl transferase center of the 50S ribosomal subunit, cross-resistance can occur in Gram-positive cocci carrying certain oxazolidinone-resistant determinants, although contezolid retains activity against some linezolid-resistant isolates. In contrast, contezolid-resistant *M*. *tuberculosis* mutants selected in vitro carried *mce3R* mutations and did not exhibit cross-resistance to linezolid. Contezolid is approved in China for complicated skin and soft tissue infections (cSSTIs), whereas its use for pneumonia and TB remains investigational or off-label [372,373,374,375,376,377,378,379,380].

**Sutezolid (PNU-100480, U-100480, PF-02341272)**. Sutezolid is an investigational oxazolidinone that inhibits bacterial protein synthesis at the 50S ribosomal subunit and has greater antimycobacterial activity than linezolid in preclinical models against both drug-susceptible and drug-resistant strains of *M*. *tuberculosis*. Preclinical studies showed reduced mitochondrial toxicity with sutezolid compared with linezolid, and a Phase IIb clinical trial involving patients with drug-susceptible pulmonary TB demonstrated favorable tolerability, supporting its potential for an improved safety profile. Preclinical combination studies support the potential of sutezolid-containing regimens to shorten TB treatment, although this treatment-shortening efficacy remains under evaluation in a Phase IIc trial. Its antimycobacterial spectrum also includes *M. avium* complex, against which it shows in vitro activity, and *M. ulcerans*, against which it demonstrates activity in a mouse model of Buruli ulcer [381,382,383,384,385,386,387,388,389].

**Pretomanid (PA-824)**. Pretomanid is an FDA-approved, oral nitroimidazooxazine indicated for use as part of combination therapy against drug-resistant *M*. *tuberculosis* (Figure 1). Its activity against both replicating bacilli and hypoxic, nonreplicating persisters contributes to its sterilizing activity and the treatment-shortening efficacy of pretomanid-containing regimens. As a prodrug, pretomanid requires activation by the deazaflavin-dependent nitroreductase (Ddn) and the reduced cofactor F_420_H_2_. Once activated, it exerts bactericidal activity through the release of reactive nitrogen species, including nitric oxide under hypoxic conditions, inhibition of mycolic acid biosynthesis, and disruption of respiratory bioenergetics. DprE2, which is involved in decaprenylphosphoryl-β-D-arabinose biosynthesis and consequently in arabinogalactan synthesis, has also been proposed as a molecular target. Resistance is primarily mediated by mutations in *ddn*, *fgd1*, *fbiA*, *fbiB*, *fbiC*, and *fbiD*, which impair prodrug activation, F_420_ biosynthesis, or cofactor reduction. Pretomanid is a key component of the BPaL and BPaLM (bedaquiline, pretomanid, linezolid, and moxifloxacin) regimens used to treat indicated forms of MDR and RR pulmonary TB, including fluoroquinolone-resistant disease when moxifloxacin is omitted. BPaMZ (bedaquiline, pretomanid, moxifloxacin, and pyrazinamide) remains an investigational regimen, and pretomanid has also been evaluated in treatment-shortening regimens for drug-susceptible TB. Preclinical studies have explored pretomanid-containing regimens for CNS-TB, but their clinical efficacy for this indication has not been established. Delamanid is another nitroimidazole used for drug-resistant TB in selected clinical settings [316,326,390,391,392,393,394,395,396,397,398,399,400,401,402,403,404,405,406].

**Delamanid (OPC-67683, Deltyba)**. Delamanid is a nitroimidazooxazole prodrug with activity against drug-susceptible and drug-resistant strains of *M*. *tuberculosis*, including MDR and rifampicin-resistant strains. It is used as part of combination regimens for adults and children with MDR/RR-TB. However, evidence supporting its use in extrapulmonary and CNS-TB remains limited and is derived mainly from pharmacokinetic studies, observational reports, and case series. It inhibits the biosynthesis of methoxy- and keto-mycolic acids. As a prodrug, it is activated by Ddn using reduced F_420_. Its activated metabolites also inhibit DprE2, disrupting cell wall arabinan biosynthesis, while reactive nitrogen species can also contribute to its antibacterial activity. Mutations in *ddn*, *fgd1*, *fbiA*, *fbiB*, *fbiC*, and *fbiD* confer resistance by impairing F_420_ biosynthesis or reduction or preventing delamanid activation. Delamanid has demonstrated antitubercular efficacy when incorporated into multidrug regimens and is included in the six-month BDLLfxC regimen (bedaquiline, delamanid, linezolid, levofloxacin, and clofazimine) for MDR/RR-TB. Bedaquiline-, delamanid-, and linezolid-containing regimens have also been used as individualized salvage therapy in selected patients with highly drug-resistant TB [401,405,407,408,409,410,411,412,413,414,415,416,417,418,419,420].

Notably, the development of antimycobacterial drugs reflects substantial progress in targeting essential pathways involved in energy metabolism, cell wall biosynthesis, and protein synthesis (Table 1). The successful development of bedaquiline and pretomanid, together with investigational antibacterials such as sudapyridine, Q203, BTZ-043, PBTZ169, quabodepistat, SQ109, and newer oxazolidinones, underscores the therapeutic potential of diverse targets and mechanisms of action against TB, including drug-resistant TB. Their incorporation into combination regimens and the continued development of safer and more potent treatments are important strategies for addressing drug resistance, reducing toxicity, and shortening TB treatment.

### 2.5. Nontraditional Treatments

**iOWH032**. iOWH032 is a synthetic small-molecule inhibitor of the cystic fibrosis transmembrane conductance regulator chloride channel investigated as an oral, antisecretory, host-directed treatment for cholera caused by *Vibrio cholerae*. In a Phase IIa controlled human infection study, iOWH032 did not significantly reduce diarrheal stool output or the severity of diarrhea compared with placebo, and its development was discontinued [421].

**Ribaxamase (SYN-004, P3A, derived from P1A)**. Ribaxamase is a first-in-class, recombinant β-lactamase that degrades susceptible β-lactam antibiotics. When administered orally in conjunction with intravenous β-lactam antibiotics, it is designed to protect the gut microbiome from antibiotic-induced disruption by degrading excess antibiotics excreted via the biliary route into the small intestine. In a Phase IIb trial involving patients receiving intravenous ceftriaxone, ribaxamase was associated with a lower incidence of *C*. *difficile* infection than placebo, while enrollment in a subsequent trial is currently paused. Its ability to prevent other antibiotic-associated diarrhea has not been established [422,423,424].

These nontraditional treatments represent host-directed and microbiome-protective approaches (Table 1), although establishing clinical benefit remains challenging. Further exploration of nontraditional strategies, including antibacterials guided by bacterial physiology and pathogenesis, is needed to expand options for preventing and treating bacterial infections.

## 3. Future Outlook

Antibacterial development since 2010 encompasses improvements to established antibiotic classes, new β-lactam/β-lactamase inhibitor combinations, siderophore cephalosporins, antibiotic hybrids, antibody-antibiotic conjugates, narrow-spectrum antibacterials, and antibacterials with novel chemical scaffolds, targets, and mechanisms of action. Despite this encouraging progress, the rate at which antibiotic resistance is emerging continues to outpace antibacterial development, necessitating greater investment in antibacterial discovery and development. The successful clinical translation and FDA approval of gepotidacin, zoliflodacin, bedaquiline, and pretomanid represent important milestones and provide valuable lessons for future antibacterial development. Future efforts should prioritize advancing promising antibacterials with novel targets or mechanisms of action, such as ibezapolstat, CRS3123, BTZ-043, and Q203. Innovative antibacterial designs that incorporate multiple targets or mechanisms of action, together with targeted antibiotic delivery, offer opportunities to enhance antibacterial activity, improve selectivity, and overcome the limitations of conventional antibiotics. Machine learning and artificial intelligence-guided methods have the potential to accelerate antibacterial discovery and development [425]. Lessons from discontinued development programs suggest that early assessment of resistance potential and safety, together with rigorous evaluation of clinical efficacy, can improve the likelihood of successful antibacterial development. However, antibiotic resistance is too complex to be addressed solely through the discovery of new antibacterials. Therefore, nontraditional treatments, such as anti-virulence therapies, host-directed therapies, microbiome-based therapeutics, antibiotic adjuvants, phage therapy, and monoclonal antibodies, should also be explored. Finally, antibiotic stewardship remains a priority, and the implementation of One Health approaches is essential to curb the emergence and spread of antibiotic resistance.

## Figures and Tables

**Table 1:** Antibacterial drugs evaluated in clinical trials from 2010 to 2026: classes, mechanisms of action, clinical applications, and development status.

Antibacterial	Class/Mechanism of Action	Clinical Applications *	Development Status **	References
***Broad-spectrum***				
Gepotidacin	Triazaacenaphthylene, inhibits DNA gyrase and topoisomerase IV and disrupts DNA replication	uUTIs, uncomplicated urogenital gonorrhea	FDA-approved	[7,8,9,10,11,12,13,14,15,16,17,18,19,20,21,22,23,24,25]
Zoliflodacin	Spiropyrimidinetrione, primarily inhibits DNA gyrase and disrupts DNA replication	Uncomplicated urogenital gonorrhea	FDA-approved	[26,27,28,29,30,31,32]
Delafloxacin	Anionic fluoroquinolone, inhibits DNA gyrase and topoisomerase IV and disrupts DNA replication	ABSSSIs, CABP	FDA-approved	[33,34,35,36,37,38,39,40,41,42,43,44,45]
Ceftobiprole	Cephalosporin, binds PBPs and inhibits cell wall synthesis	ABSSSIs, bloodstream infections due to *S*. *aureus* (including right-sided infective endocarditis), CABP	FDA-approved	[46,47,48,49,50,51,52,53,54]
Ceftaroline	Cephalosporin, binds PBPs and inhibits cell wall synthesis	ABSSSIs, CABP	FDA-approved	[55,56,57,58,59,60,61,62,63,64,65,66]
GSK2251052	Oxaborole, inhibits leucyl-tRNA synthetase and protein synthesis	cUTIs studied	Development for cUTIs discontinued	[67,68,69,70,71,72,73,74,75]
GSK1322322	Hydrazinopyrimidine, inhibits peptide deformylase and disrupts protein maturation	CABP, ABSSSIs studied	Phase IIa completed, development discontinued	[76,77,78]
Plazomicin	Aminoglycoside, binds the 30S ribosomal subunit and inhibits protein synthesis	cUTIs, including acute pyelonephritis	FDA-approved	[79,80,81,82,83,84,85,86,87,88,89,90,91,92]
Lefamulin	Pleuromutilin, binds the 50S ribosomal subunit and inhibits protein synthesis	CABP, ABSSSIs studied	FDA-approved for CABP	[93,94,95,96,97]
Solithromycin	Fluoroketolide, binds the 50S ribosomal subunit and inhibits protein synthesis	CABP and uncomplicated urogenital gonorrhea studied	Phase III completed, FDA Complete Response Letter issued in 2016, not approved	[98,99,100,101,102,103,104,105,106,107,108,109,110,111,112]
NXL-103	Streptogramin combination, binds the 50S ribosomal subunit and inhibits protein synthesis	CABP, ABSSSIs studied	Phase II completed, subsequent development status unclear	[113,114,115,116]
Iclaprim	Diaminopyrimidine, inhibits dihydrofolate reductase and disrupts folate metabolism and DNA synthesis	ABSSSIs, nosocomial pneumonia studied	Phase III completed, development discontinued	[117,118,119,120,121,122,123,124,125]
NVC-422	Synthetic chloramine, oxidatively inactivates microbial components	Impetigo, ocular infections, and prevention of urinary catheter blockage studied	Phase IIb completed, subsequent development status unclear	[126,127,128,129,130]
LTX-109	Peptidomimetic, disrupts bacterial membrane and causes cell lysis	Topical skin infections, *S*. *aureus* nasal decolonization studied	Phase II completed	[131,132]
Rifasutenizol	Rifamycin-nitroimidazole conjugate, inhibits RNA synthesis and causes DNA damage	*H*. *pylori* infection, bacterial vaginosis studied	Phase III completed, application for approval under review in China	[133,134,135]
***Gram-positive infections***				
Dalbavancin	Lipoglycopeptide, inhibits cell wall synthesis	ABSSSIs	FDA-approved	[136,137,138,139,140,141,142,143,144,145,146,147,148,149]
Oritavancin	Lipoglycopeptide, inhibits cell wall synthesis and disrupts bacterial membranes	ABSSSIs	FDA-approved	[150,151,152,153,154,155,156,157]
TD-1792	Glycopeptide-cephalosporin heterodimer, inhibits cell wall synthesis	cSSSIs studied	Phase III initiated, current status unclear	[158,159,160,161,162]
Tedizolid phosphate	Oxazolidinone, inhibits protein synthesis	ABSSSIs, HABP/VABP and bone and joint infections studied	FDA-approved for ABSSSIs	[163,164,165,166,167,168,169]
Cadazolid	Fluoroquinolone-oxazolidinone hybrid, primarily inhibits protein synthesis	*C*. *difficile* infection	Development discontinued	[170,171,172,173,174]
Fusidic acid	Steroidal antibiotic, inhibits protein synthesis	ABSSSIs and prosthetic joint infections investigated	Approved outside the United States, U.S. development inactive	[175,176,177,178,179]
CRS3123	Diaryldiamine, inhibits methionyl-tRNA synthetase and protein synthesis	*C*. *difficile* infection	Phase II completed	[180,181,182,183,184,185]
Ibezapolstat	Guanine derivative, inhibits DNA polymerase IIIC and DNA replication	*C*. *difficile* infection	Phase IIb completed, Phase III planned	[186,187,188,189,190,191]
DS-2969b	Imidazole-2-carboxamide, inhibits DNA gyrase subunit B and DNA replication	*C*. *difficile* infection	Development discontinued	[192,193,194]
Ridinilazole	Bis-benzimidazole, binds the DNA minor groove and disrupts transcription	*C*. *difficile* infection	Phase III completed, further clinical development planned	[195,196,197,198,199,200,201]
Fidaxomicin	Macrocyclic antibiotic, inhibits transcription	*C*. *difficile*-associated diarrhea	FDA-approved	[202,203,204,205,206,207,208,209,210,211,212,213,214,215,216]
Surotomycin	Cyclic lipopeptide, disrupts bacterial membranes	*C*. *difficile* infection	Development discontinued	[217,218,219,220,221,222,223,224,225]
XF-73	Dicationic porphyrin, disrupts bacterial membranes	*S*. *aureus* nasal decolonization	Phase II completed	[226,227,228,229,230]
Afabicin	Benzofuran-naphthyridinone, inhibits enoyl-acyl carrier protein reductase and fatty acid synthesis	ABSSSIs and bone and joint infections investigated	Phase II completed	[231,232,233,234]
DSTA4637S	Antibody-antibiotic conjugate, inhibits transcription	*S*. *aureus* bacteremia investigated	Phase Ib evaluated, current status unclear	[235,236]
α-Viniferin	Phytochemical with antistaphylococcal activity, precise target undetermined	*S*. *aureus* nasal decolonization investigated	Development status unclear	[237,238,239]
***Gram-negative infections***				
HRS-8427	Siderophore cephalosporin, inhibits cell wall synthesis	cUTIs, HABP/VABP	Phase II/III	[240,241,242]
Cefiderocol	Siderophore cephalosporin, inhibits cell wall synthesis	cUTIs, HABP/VABP	FDA-approved	[243,244,245,246,247,248,249,250,251,252,253,254,255,256]
Benapenem	Carbapenem, inhibits cell wall synthesis	cUTIs, including acute pyelonephritis	Phase II/III	[257]
Cefepime-taniborbactam	Cephalosporin-β-lactamase inhibitor combination, inhibits cell wall synthesis and β-lactamases	cUTIs, including acute pyelonephritis	Phase III completed, FDA complete response letter issued	[258,259,260,261,262,263,264,265,266,267,268,269]
Ceftazidime-avibactam	Cephalosporin-β-lactamase inhibitor combination, inhibits cell wall synthesis and β-lactamases	cUTIs, cIAIs, HABP/VABP	FDA-approved	[270,271,272,273,274,275,276,277,278,279,280,281]
Ceftolozane-tazobactam	Cephalosporin-β-lactamase inhibitor combination, inhibits cell wall synthesis and β-lactamases	cUTIs, cIAIs, HABP/VABP	FDA-approved	[282,283,284,285,286,287,288,289,290,291,292,293,294,295,296,297,298]
Sulbactam-durlobactam	β-lactam-β-lactamase inhibitor combination, inhibits cell wall synthesis and β-lactamases	HABP/VABP caused by susceptible *A*. *baumannii*-*calcoaceticus* complex isolates	FDA-approved	[299,300,301,302,303,304]
***Mycobacterial infections***				
Bedaquiline	Diarylquinoline, inhibits ATP synthase and depletes ATP	Pulmonary TB due to MDR *M*. *tuberculosis* (in combination therapy)	FDA-approved	[305,306,307,308,309,310,311,312,313,314,315,316,317,318,319,320,321,322,323,324,325,326,327,328,329,330,331,332]
Sudapyridine	Bedaquiline analog, inhibits ATP synthase and disrupts proton motive force	RR pulmonary TB due to *M*. *tuberculosis* studied	Phase III	[333,334,335,336,337,338,339,340,341]
Q203	Imidazopyridine amide, inhibits QcrB and cellular respiration	Drug-susceptible pulmonary TB due to *M*. *tuberculosis* studied, Buruli ulcer due to *M*. *ulcerans* studied	Phase II	[342,343,344,345,346]
BTZ-043	Benzothiazinone, inhibits DprE1 and cell wall arabinan synthesis	Drug-susceptible pulmonary TB due to *M*. *tuberculosis* studied	Phase IIb/c	[347,348,349,350,351,352,353,354]
PBTZ169	Benzothiazinone, inhibits DprE1 and cell wall arabinan synthesis	Drug-susceptible pulmonary TB due to *M*. *tuberculosis* studied	Phase II	[355,356,357]
Quabodepistat	Carbostyril, inhibits DprE1 and cell wall arabinan synthesis	Drug-susceptible and MDR pulmonary TB due to *M*. *tuberculosis* studied	Phase III ongoing	[358,359,360]
SQ109	Ethambutol-related diamine, inhibits MmpL3 and disrupts proton motive force	Drug-susceptible and MDR pulmonary TB due to *M*. *tuberculosis* studied	Phase IIb/III completed	[361,362,363,364,365,366]
Posizolid	Oxazolidinone, inhibits protein synthesis	Drug-susceptible pulmonary TB due to *M*. *tuberculosis* studied	Development discontinued	[367,368,369,370,371]
Contezolid	Oxazolidinone, inhibits protein synthesis	cSSTIs due to susceptible Gram-positive bacteria, pneumonia studied, pulmonary TB due to *M*. *tuberculosis* investigated	Approved in China for cSSTIs, TB use investigational	[372,373,374,375,376,377,378,379,380]
Sutezolid	Oxazolidinone, inhibits protein synthesis	Drug-susceptible pulmonary TB due to *M*. *tuberculosis* studied	Phase IIb/c	[381,382,383,384,385,386,387,388,389]
Pretomanid	Nitroimidazooxazine, inhibits cell wall synthesis and respiration	Drug-resistant pulmonary TB due to *M*. *tuberculosis* (in combination regimens)	FDA-approved	[316,326,390,391,392,393,394,395,396,397,398,399,400,401,402,403,404,405,406]
Delamanid	Nitroimidazooxazole, inhibits mycolic acid synthesis	MDR/RR-TB due to *M*. *tuberculosis* (in combination regimens)	Approved outside the U. S.	[401,405,407,408,409,410,411,412,413,414,415,416,417,418,419,420]
***Nontraditional treatments***				
iOWH032	Host-directed oxadiazole-carboxamide, inhibits the cystic fibrosis transmembrane conductance regulator chloride channel and reduces intestinal chloride secretion	Cholera	Development discontinued	[421]
Ribaxamase	Recombinant β-lactamase, degrades β-lactam antibiotics released into the intestine to protect the gut microbiome	Prevention of *C*. *difficile* infection during intravenous β-lactam treatment	Phase IIb, development paused	[422,423,424]

* Clinical applications include approved indications and conditions evaluated in clinical studies. Conditions evaluated in clinical studies are not necessarily approved indications. ** Development status reflects publicly available information as of August 2026.

## Data Availability

Not applicable.

## References

[1] Naghavi M., Vollset S.E., Ikuta K.S., Swetschinski L.R., Gray A.P., Wool E.E., Robles Aguilar G., Mestrovic T., Smith G., Han C. (2024). Global burden of bacterial antimicrobial resistance 1990–2021: A systematic analysis with forecasts to 2050. Lancet.

[2] Theuretzbacher U., Jumde R.P., Hennessy A., Cohn J., Piddock L.J.V. (2025). Global health perspectives on antibacterial drug discovery and the preclinical pipeline. Nat. Rev. Microbiol..

[3] Theuretzbacher U. (2025). The global resistance problem and the clinical antibacterial pipeline. Nat. Rev. Microbiol..

[4] World Health Organization (World Health Organization) (2024). WHO Bacterial Priority Pathogens List, 2024: Bacterial Pathogens of Public Health Importance to Guide Research, Development and Strategies to Prevent and Control Antimicrobial Resistance.

[5] Lewis K. (2013). Platforms for antibiotic discovery. Nat. Rev. Drug Discov..

[6] Theuretzbacher U., Blasco B., Duffey M., Piddock L.J.V. (2023). Unrealized targets in the discovery of antibiotics for Gram-negative bacterial infections. Nat. Rev. Drug Discov..

[7] Ross J.D.C., Wilson J., Workowski K.A., Taylor S.N., Lewis D.A., Gatsi S., Flight W., Scangarella-Oman N.E., Jakielaszek C., Lythgoe D. (2025). Oral gepotidacin for the treatment of uncomplicated urogenital gonorrhoea (EAGLE-1): A phase 3 randomised, open-label, non-inferiority, multicentre study. Lancet.

[8] Scangarella-Oman N.E., Hossain M., Hoover J.L., Perry C.R., Tiffany C., Barth A., Dumont E.F. (2022). Dose Selection for Phase III Clinical Evaluation of Gepotidacin (GSK2140944) in the Treatment of Uncomplicated Urinary Tract Infections. Antimicrob. Agents Chemother..

[9] Biedenbach D.J., Bouchillon S.K., Hackel M., Miller L.A., Scangarella-Oman N.E., Jakielaszek C., Sahm D.F. (2016). In Vitro Activity of Gepotidacin, a Novel Triazaacenaphthylene Bacterial Topoisomerase Inhibitor, against a Broad Spectrum of Bacterial Pathogens. Antimicrob. Agents Chemother..

[10] Farrell D.J., Sader H.S., Rhomberg P.R., Scangarella-Oman N.E., Flamm R.K. (2017). In Vitro Activity of Gepotidacin (GSK2140944) against Neisseria gonorrhoeae. Antimicrob. Agents Chemother..

[11] O’Riordan W., Tiffany C., Scangarella-Oman N., Perry C., Hossain M., Ashton T., Dumont E. (2017). Efficacy, Safety, and Tolerability of Gepotidacin (GSK2140944) in the Treatment of Patients with Suspected or Confirmed Gram-Positive Acute Bacterial Skin and Skin Structure Infections. Antimicrob. Agents Chemother..

[12] Flamm R.K., Farrell D.J., Rhomberg P.R., Scangarella-Oman N.E., Sader H.S. (2017). Gepotidacin (GSK2140944) In Vitro Activity against Gram-Positive and Gram-Negative Bacteria. Antimicrob. Agents Chemother..

[13] Waites K.B., Crabb D.M., Xiao L., Duffy L.B. (2017). In Vitro Activities of Gepotidacin (GSK2140944) and Other Antimicrobial Agents against Human Mycoplasmas and Ureaplasmas. Antimicrob. Agents Chemother..

[14] Taylor S.N., Morris D.H., Avery A.K., Workowski K.A., Batteiger B.E., Tiffany C.A., Perry C.R., Raychaudhuri A., Scangarella-Oman N.E., Hossain M. (2018). Gepotidacin for the Treatment of Uncomplicated Urogenital Gonorrhea: A Phase 2, Randomized, Dose-Ranging, Single-Oral Dose Evaluation. Clin. Infect. Dis..

[15] Jacobsson S., Golparian D., Scangarella-Oman N., Unemo M. (2018). In vitro activity of the novel triazaacenaphthylene gepotidacin (GSK2140944) against MDR Neisseria gonorrhoeae. J. Antimicrob. Chemother..

[16] Scangarella-Oman N.E., Ingraham K.A., Tiffany C.A., Tomsho L., Van Horn S.F., Mayhew D.N., Perry C.R., Ashton T.C., Dumont E.F., Huang J. (2020). In Vitro Activity and Microbiological Efficacy of Gepotidacin from a Phase 2, Randomized, Multicenter, Dose-Ranging Study in Patients with Acute Bacterial Skin and Skin Structure Infections. Antimicrob. Agents Chemother..

[17] Overcash J.S., Tiffany C.A., Scangarella-Oman N.E., Perry C.R., Tao Y., Hossain M., Barth A., Dumont E.F. (2020). Phase 2a Pharmacokinetic, Safety, and Exploratory Efficacy Evaluation of Oral Gepotidacin (GSK2140944) in Female Participants with Uncomplicated Urinary Tract Infection (Acute Uncomplicated Cystitis). Antimicrob. Agents Chemother..

[18] Jensen J.S., Nørgaard C., Scangarella-Oman N., Unemo M. (2020). In vitro activity of the first-in-class triazaacenaphthylene gepotidacin alone and in combination with doxycycline against drug-resistant and -susceptible Mycoplasma genitalium. Emerg. Microbes Infect..

[19] Hackel M.A., Karlowsky J.A., Canino M.A., Sahm D.F., Scangarella-Oman N.E. (2022). In Vitro Activity of Gepotidacin against Gram-Negative and Gram-Positive Anaerobes. Antimicrob. Agents Chemother..

[20] Jakielaszek C., Hossain M., Qian L., Fishman C., Widdowson K., Hilliard J.J., Mannino F., Raychaudhuri A., Carniel E., Demons S. (2022). Gepotidacin is efficacious in a nonhuman primate model of pneumonic plague. Sci. Transl. Med..

[21] Ahmad M.N., Garg T., Singh S., Shukla R., Malik P., Krishnamurthy R.V., Kaur P., Chopra S., Dasgupta A. (2022). In Vitro and In Vivo Activity of Gepotidacin against Drug-Resistant Mycobacterial Infections. Antimicrob. Agents Chemother..

[22] Hilliard J.J., Jakielaszek C., Mannino F., Hossain M., Qian L., Fishman C., Demons S., Hershfield J., Soffler C., Russo R. (2024). Efficacy of therapeutically administered gepotidacin in a rabbit model of inhalational anthrax. Antimicrob. Agents Chemother..

[23] Brown-Elliott B.A., Bush G., Hughes M.D., Rodriguez E., Weikel C.A., Min S.B., Wallace R.J. (2024). In vitro activity of gepotidacin and comparator antimicrobials against isolates of nontuberculous mycobacteria (NTM). Antimicrob. Agents Chemother..

[24] Hackel M.A., Karlowsky J.A., Sahm D.F., West J.M., Scangarella-Oman N.E. (2025). In vitro activity of gepotidacin against urinary tract infection isolates of Enterobacterales, Enterococcus faecalis, and Staphylococcus saprophyticus. Antimicrob. Agents Chemother..

[25] Touati A., Peuchant O., Weikel C.A., Bébéar C. (2026). In vitro activity of gepotidacin against Chlamydia trachomatis. J. Antimicrob. Chemother..

[26] Jacobsson S., Golparian D., Alm R.A., Huband M., Mueller J., Jensen J.S., Ohnishi M., Unemo M. (2014). High in vitro activity of the novel spiropyrimidinetrione AZD0914, a DNA gyrase inhibitor, against multidrug-resistant Neisseria gonorrhoeae isolates suggests a new effective option for oral treatment of gonorrhea. Antimicrob. Agents Chemother..

[27] Kohlhoff S.A., Huband M.D., Hammerschlag M.R. (2014). In vitro activity of AZD0914, a novel DNA gyrase inhibitor, against Chlamydia trachomatis and Chlamydia pneumoniae. Antimicrob. Agents Chemother..

[28] Huband M.D., Bradford P.A., Otterson L.G., Basarab G.S., Kutschke A.C., Giacobbe R.A., Patey S.A., Alm R.A., Johnstone M.R., Potter M.E. (2015). In vitro antibacterial activity of AZD0914, a new spiropyrimidinetrione DNA gyrase/topoisomerase inhibitor with potent activity against Gram-positive, fastidious Gram-Negative, and atypical bacteria. Antimicrob. Agents Chemother..

[29] Waites K.B., Crabb D.M., Duffy L.B., Huband M.D. (2015). In vitro antibacterial activity of AZD0914 against human Mycoplasmas and Ureaplasmas. Antimicrob. Agents Chemother..

[30] Taylor S.N., Marrazzo J., Batteiger B.E., Hook E.W., Seña A.C., Long J., Wierzbicki M.R., Kwak H., Johnson S.M., Lawrence K. (2018). Single-Dose Zoliflodacin (ETX0914) for Treatment of Urogenital Gonorrhea. N. Engl. J. Med..

[31] Liu J., Jia J., Shi T., Bai Y., Huang Y., Zeng L., Bi H. (2024). Evaluating the potency of zoliflodacin against Helicobacter pylori: In vitro activity and conserved GyrB target. Helicobacter.

[32] Luckey A., Balasegaram M., Barbee L.A., Batteiger T.A., Broadhurst H., Cohen S.E., Delany-Moretlwe S., de Vries H.J.C., Dionne J.A., Gill K. (2026). Zoliflodacin versus ceftriaxone plus azithromycin for treatment of uncomplicated urogenital gonorrhoea: An international, randomised, controlled, open-label, phase 3, non-inferiority clinical trial. Lancet.

[33] Kingsley J., Mehra P., Lawrence L.E., Henry E., Duffy E., Cammarata S.K., Pullman J. (2016). A randomized, double-blind, Phase 2 study to evaluate subjective and objective outcomes in patients with acute bacterial skin and skin structure infections treated with delafloxacin, linezolid or vancomycin. J. Antimicrob. Chemother..

[34] Goldstein E.J., Citron D.M., Merriam C.V., Warren Y.A., Tyrrell K.L., Fernandez H.T. (2003). In vitro activities of ABT-492, a new fluoroquinolone, against 155 aerobic and 171 anaerobic pathogens isolated from antral sinus puncture specimens from patients with sinusitis. Antimicrob. Agents Chemother..

[35] Nilius A.M., Shen L.L., Hensey-Rudloff D., Almer L.S., Beyer J.M., Balli D.J., Cai Y., Flamm R.K. (2003). In vitro antibacterial potency and spectrum of ABT-492, a new fluoroquinolone. Antimicrob. Agents Chemother..

[36] Waites K.B., Crabb D.M., Duffy L.B. (2003). Comparative in vitro susceptibilities and bactericidal activities of investigational fluoroquinolone ABT-492 and other antimicrobial agents against human mycoplasmas and ureaplasmas. Antimicrob. Agents Chemother..

[37] Harnett S.J., Fraise A.P., Andrews J.M., Jevons G., Brenwald N.P., Wise R. (2004). Comparative study of the in vitro activity of a new fluoroquinolone, ABT-492. J. Antimicrob. Chemother..

[38] Almer L.S., Hoffrage J.B., Keller E.L., Flamm R.K., Shortridge V.D. (2004). In vitro and bactericidal activities of ABT-492, a novel fluoroquinolone, against Gram-positive and Gram-negative organisms. Antimicrob. Agents Chemother..

[39] Bauer J., Siala W., Tulkens P.M., Van Bambeke F. (2013). A combined pharmacodynamic quantitative and qualitative model reveals the potent activity of daptomycin and delafloxacin against *Staphylococcus aureus* biofilms. Antimicrob. Agents Chemother..

[40] Thabit A.K., Crandon J.L., Nicolau D.P. (2016). Pharmacodynamic and pharmacokinetic profiling of delafloxacin in a murine lung model against community-acquired respiratory tract pathogens. Int. J. Antimicrob. Agents.

[41] Boyanova L., Yordanov D., Ouzounova-Raykova V., Markovska R., Marteva-Proevska Y., Mitov I. (2020). Activity of delafloxacin versus that of levofloxacin against anaerobic and microaerophilic isolates. Anaerobe.

[42] Brown Elliott B.A., Wallace R.J. (2021). Comparison of In Vitro Susceptibility of Delafloxacin with Ciprofloxacin, Moxifloxacin, and Other Comparator Antimicrobials against Isolates of Nontuberculous Mycobacteria. Antimicrob. Agents Chemother..

[43] McCurdy S., Duffy E., Hickman M., Halasohoris S., Zumbrun S.D. (2021). Efficacy of Delafloxacin against the Biothreat Pathogen Burkholderia pseudomallei. Antimicrob. Agents Chemother..

[44] McCurdy S., Halasohoris S.A., Babyak A.L., Lembirik S., Hoover R., Hickman M., Scarff J., Klimko C.P., Cote C.K., Meinig J.M. (2023). Efficacy of delafloxacin against the biothreat pathogen Bacillus anthracis. J. Antimicrob. Chemother..

[45] Najmudin S., Pan X.S., Wang B., Govada L., Chayen N.E., Rubio N., Shaffer M.S.P., Rzepa H.S., Fisher L.M., Sanderson M.R. (2025). Structural basis of topoisomerase targeting by delafloxacin. Nat. Commun..

[46] Overcash J.S., Kim C., Keech R., Gumenchuk I., Ninov B., Gonzalez-Rojas Y., Waters M., Simeonov S., Engelhardt M., Saulay M. (2021). Ceftobiprole Compared With Vancomycin Plus Aztreonam in the Treatment of Acute Bacterial Skin and Skin Structure Infections: Results of a Phase 3, Randomized, Double-blind Trial (TARGET). Clin. Infect. Dis..

[47] Hebeisen P., Heinze-Krauss I., Angehrn P., Hohl P., Page M.G., Then R.L. (2001). In vitro and in vivo properties of Ro 63-9141, a novel broad-spectrum cephalosporin with activity against methicillin-resistant staphylococci. Antimicrob. Agents Chemother..

[48] Jones R.N., Deshpande L.M., Mutnick A.H., Biedenbach D.J. (2002). In vitro evaluation of BAL9141, a novel parenteral cephalosporin active against oxacillin-resistant staphylococci. J. Antimicrob. Chemother..

[49] Bogdanovich T., Clark C., Ednie L., Lin G., Smith K., Shapiro S., Appelbaum P.C. (2006). Activities of ceftobiprole, a novel broad-spectrum cephalosporin, against Haemophilus influenzae and Moraxella catarrhalis. Antimicrob. Agents Chemother..

[50] Noel G.J., Strauss R.S., Amsler K., Heep M., Pypstra R., Solomkin J.S. (2008). Results of a double-blind, randomized trial of ceftobiprole treatment of complicated skin and skin structure infections caused by gram-positive bacteria. Antimicrob. Agents Chemother..

[51] Awad S.S., Rodriguez A.H., Chuang Y.C., Marjanek Z., Pareigis A.J., Reis G., Scheeren T.W., Sánchez A.S., Zhou X., Saulay M. (2014). A phase 3 randomized double-blind comparison of ceftobiprole medocaril versus ceftazidime plus linezolid for the treatment of hospital-acquired pneumonia. Clin. Infect. Dis..

[52] Turner J.M., Connolly K.L., Aberman K.E., Fonseca J.C., Singh A., Jerse A.E., Nicholas R.A., Davies C. (2021). Molecular Features of Cephalosporins Important for Activity against Antimicrobial-Resistant Neisseria gonorrhoeae. ACS Infect. Dis..

[53] Holland T.L., Cosgrove S.E., Doernberg S.B., Jenkins T.C., Turner N.A., Boucher H.W., Pavlov O., Titov I., Kosulnykov S., Atanasov B. (2023). Ceftobiprole for Treatment of Complicated *Staphylococcus aureus* Bacteremia. N. Engl. J. Med..

[54] Hahn M.M., Anderson M.S., Snyder M., Du W., Chou Y.L., McCracken B., Smart J.I., Litherland K., Keech S., von Siebenthal F. (2025). Pharmacokinetic modeling and efficacy of ceftobiprole medocaril against pneumonic tularemia in cynomolgus macaques. Antimicrob. Agents Chemother..

[55] Ishikawa T., Matsunaga N., Tawada H., Kuroda N., Nakayama Y., Ishibashi Y., Tomimoto M., Ikeda Y., Tagawa Y., Iizawa Y. (2003). TAK-599, a novel N-phosphono type prodrug of anti-MRSA cephalosporin T-91825: Synthesis, physicochemical and pharmacological properties. Bioorg. Med. Chem..

[56] Jacqueline C., Caillon J., Le Mabecque V., Miègeville A.F., Hamel A., Bugnon D., Ge J.Y., Potel G. (2007). In vivo efficacy of ceftaroline (PPI-0903), a new broad-spectrum cephalosporin, compared with linezolid and vancomycin against methicillin-resistant and vancomycin-intermediate *Staphylococcus aureus* in a rabbit endocarditis model. Antimicrob. Agents Chemother..

[57] Talbot G.H., Thye D., Das A., Ge Y. (2007). Phase 2 study of ceftaroline versus standard therapy in treatment of complicated skin and skin structure infections. Antimicrob. Agents Chemother..

[58] Ge Y., Biek D., Talbot G.H., Sahm D.F. (2008). In vitro profiling of ceftaroline against a collection of recent bacterial clinical isolates from across the United States. Antimicrob. Agents Chemother..

[59] Sader H.S., Fritsche T.R., Kaniga K., Ge Y., Jones R.N. (2005). Antimicrobial activity and spectrum of PPI-0903M (T-91825), a novel cephalosporin, tested against a worldwide collection of clinical strains. Antimicrob. Agents Chemother..

[60] Jacqueline C., Caillon J., Le Mabecque V., Miègeville A.F., Ge Y., Biek D., Batard E., Potel G. (2009). In vivo activity of a novel anti-methicillin-resistant *Staphylococcus aureus* cephalosporin, ceftaroline, against vancomycin-susceptible and -resistant Enterococcus faecalis strains in a rabbit endocarditis model: A comparative study with linezolid and vancomycin. Antimicrob. Agents Chemother..

[61] Citron D.M., Tyrrell K.L., Merriam C.V., Goldstein E.J. (2010). In vitro activity of ceftaroline against 623 diverse strains of anaerobic bacteria. Antimicrob. Agents Chemother..

[62] Panagiotidis G., Bäckström T., Asker-Hagelberg C., Jandourek A., Weintraub A., Nord C.E. (2010). Effect of ceftaroline on normal human intestinal microflora. Antimicrob. Agents Chemother..

[63] Long S.W., Olsen R.J., Mehta S.C., Palzkill T., Cernoch P.L., Perez K.K., Musick W.L., Rosato A.E., Musser J.M. (2014). PBP2a mutations causing high-level Ceftaroline resistance in clinical methicillin-resistant *Staphylococcus aureus* isolates. Antimicrob. Agents Chemother..

[64] Barber K.E., Smith J.R., Ireland C.E., Boles B.R., Rose W.E., Rybak M.J. (2015). Evaluation of Ceftaroline Alone and in Combination against Biofilm-Producing Methicillin-Resistant *Staphylococcus aureus* with Reduced Susceptibility to Daptomycin and Vancomycin in an In Vitro Pharmacokinetic/Pharmacodynamic Model. Antimicrob. Agents Chemother..

[65] Meeker D.G., Beenken K.E., Mills W.B., Loughran A.J., Spencer H.J., Lynn W.B., Smeltzer M.S. (2016). Evaluation of Antibiotics Active against Methicillin-Resistant *Staphylococcus aureus* Based on Activity in an Established Biofilm. Antimicrob. Agents Chemother..

[66] Hamilton S.M., Alexander J.A.N., Choo E.J., Basuino L., da Costa T.M., Severin A., Chung M., Aedo S., Strynadka N.C.J., Tomasz A. (2017). High-Level Resistance of *Staphylococcus aureus* to β-Lactam Antibiotics Mediated by Penicillin-Binding Protein 4 (PBP4). Antimicrob. Agents Chemother..

[67] O’Dwyer K., Spivak A.T., Ingraham K., Min S., Holmes D.J., Jakielaszek C., Rittenhouse S., Kwan A.L., Livi G.P., Sathe G. (2015). Bacterial resistance to leucyl-tRNA synthetase inhibitor GSK2251052 develops during treatment of complicated urinary tract infections. Antimicrob. Agents Chemother..

[68] Hernandez V., Crépin T., Palencia A., Cusack S., Akama T., Baker S.J., Bu W., Feng L., Freund Y.R., Liu L. (2013). Discovery of a novel class of boron-based antibacterials with activity against gram-negative bacteria. Antimicrob. Agents Chemother..

[69] Goldstein E.J., Citron D.M., Tyrrell K.L., Merriam C.V. (2013). Comparative in vitro activities of GSK2251052, a novel boron-containing leucyl-tRNA synthetase inhibitor, against 916 anaerobic organisms. Antimicrob. Agents Chemother..

[70] Mendes R.E., Alley M.R., Sader H.S., Biedenbach D.J., Jones R.N. (2013). Potency and spectrum of activity of AN3365, a novel boron-containing protein synthesis inhibitor, tested against clinical isolates of Enterobacteriaceae and nonfermentative Gram-negative bacilli. Antimicrob. Agents Chemother..

[71] Purnapatre K.P., Rao M., Pandya M., Khanna A., Chaira T., Bambal R., Upadhyay D.J., Masuda N. (2018). In Vitro and In Vivo Activities of DS86760016, a Novel Leucyl-tRNA Synthetase Inhibitor for Gram-Negative Pathogens. Antimicrob. Agents Chemother..

[72] Ganapathy U.S., Gengenbacher M., Dick T. (2021). Epetraborole Is Active against Mycobacterium abscessus. Antimicrob. Agents Chemother..

[73] Nguyen T.Q., Heo B.E., Hanh B.T.B., Jeon S., Park Y., Choudhary A., Lee S., Kim T.H., Moon C., Min S.J. (2023). DS86760016, a Leucyl-tRNA Synthetase Inhibitor, Is Active against Mycobacterium abscessus. Antimicrob. Agents Chemother..

[74] Cummings J.E., Lunde C.S., Alley M.R.K., Slayden R.A. (2023). Epetraborole, a leucyl-tRNA synthetase inhibitor, demonstrates murine efficacy, enhancing the in vivo activity of ceftazidime against Burkholderia pseudomallei, the causative agent of melioidosis. PLoS Negl. Trop. Dis..

[75] Abdelsattar A.S., Kikiowo B., Abutaleb N.S., Dove A.S., Seleem M.N. (2026). Repurposing epetraborole to combat Neisseria gonorrhoeae and Chlamydia trachomatis infections. Antimicrob. Agents Chemother..

[76] Hoover J., Lewandowski T., Straub R.J., Novick S.J., DeMarsh P., Aubart K., Rittenhouse S., Zalacain M. (2016). Pharmacokinetics/Pharmacodynamics of Peptide Deformylase Inhibitor GSK1322322 against Streptococcus pneumoniae, Haemophilus influenzae, and *Staphylococcus aureus* in Rodent Models of Infection. Antimicrob. Agents Chemother..

[77] Corey R., Naderer O.J., O’Riordan W.D., Dumont E., Jones L.S., Kurtinecz M., Zhu J.Z. (2014). Safety, tolerability, and efficacy of GSK1322322 in the treatment of acute bacterial skin and skin structure infections. Antimicrob. Agents Chemother..

[78] O’Dwyer K., Hackel M., Hightower S., Hoban D., Bouchillon S., Qin D., Aubart K., Zalacain M., Butler D. (2013). Comparative analysis of the antibacterial activity of a novel peptide deformylase inhibitor, GSK1322322. Antimicrob. Agents Chemother..

[79] Wagenlehner F.M.E., Cloutier D.J., Komirenko A.S., Cebrik D.S., Krause K.M., Keepers T.R., Connolly L.E., Miller L.G., Friedland I., Dwyer J.P. (2019). Once-Daily Plazomicin for Complicated Urinary Tract Infections. N. Engl. J. Med..

[80] Endimiani A., Hujer K.M., Hujer A.M., Armstrong E.S., Choudhary Y., Aggen J.B., Bonomo R.A. (2009). ACHN-490, a neoglycoside with potent in vitro activity against multidrug-resistant Klebsiella pneumoniae isolates. Antimicrob. Agents Chemother..

[81] Landman D., Babu E., Shah N., Kelly P., Bäcker M., Bratu S., Quale J. (2010). Activity of a novel aminoglycoside, ACHN-490, against clinical isolates of Escherichia coli and Klebsiella pneumoniae from New York City. J. Antimicrob. Chemother..

[82] Livermore D.M., Mushtaq S., Warner M., Zhang J.C., Maharjan S., Doumith M., Woodford N. (2011). Activity of aminoglycosides, including ACHN-490, against carbapenem-resistant Enterobacteriaceae isolates. J. Antimicrob. Chemother..

[83] Reyes N., Aggen J.B., Kostrub C.F. (2011). In vivo efficacy of the novel aminoglycoside ACHN-490 in murine infection models. Antimicrob. Agents Chemother..

[84] Tenover F.C., Tickler I., Armstrong E.S., Kubo A., Lopez S., Persing D.H., Miller G.H. (2011). Activity of ACHN-490 against meticillin-resistant *Staphylococcus aureus* (MRSA) isolates from patients in US hospitals. Int. J. Antimicrob. Agents.

[85] Walkty A., Adam H., Baxter M., Denisuik A., Lagacé-Wiens P., Karlowsky J.A., Hoban D.J., Zhanel G.G. (2014). In vitro activity of plazomicin against 5,015 gram-negative and gram-positive clinical isolates obtained from patients in canadian hospitals as part of the CANWARD study, 2011–2012. Antimicrob. Agents Chemother..

[86] Olsen S.C., Carlson S.A. (2015). In vitro bactericidal activity of aminoglycosides, including the next-generation drug plazomicin, against Brucella spp. Int. J. Antimicrob. Agents.

[87] Mega W.M., Doyle-Eisele M., Cass R.T., Kostrub C.F., Sherwood R.L., Metz M.A., Cirz R.T. (2016). Plazomicin is effective in a non-human primate pneumonic plague model. Bioorg Med. Chem..

[88] López-Diaz M.D., Culebras E., Rodríguez-Avial I., Rios E., Viñuela-Prieto J.M., Picazo J.J., Rodríguez-Avial C. (2017). Plazomicin Activity against 346 Extended-Spectrum-β-Lactamase/AmpC-Producing Escherichia coli Urinary Isolates in Relation to Aminoglycoside-Modifying Enzymes. Antimicrob. Agents Chemother..

[89] Denervaud-Tendon V., Poirel L., Connolly L.E., Krause K.M., Nordmann P. (2017). Plazomicin activity against polymyxin-resistant Enterobacteriaceae, including MCR-1-producing isolates. J. Antimicrob. Chemother..

[90] Connolly L.E., Riddle V., Cebrik D., Armstrong E.S., Miller L.G. (2018). A Multicenter, Randomized, Double-Blind, Phase 2 Study of the Efficacy and Safety of Plazomicin Compared with Levofloxacin in the Treatment of Complicated Urinary Tract Infection and Acute Pyelonephritis. Antimicrob. Agents Chemother..

[91] Hikal A.F., Zhao S., Foley S., Khan A.A. (2025). The newly identified grdA gene confers high-level plazomicin resistance in Salmonella enterica serovars. Antimicrob. Agents Chemother..

[92] Landman D., Kelly P., Bäcker M., Babu E., Shah N., Bratu S., Quale J. (2011). Antimicrobial activity of a novel aminoglycoside, ACHN-490, against Acinetobacter baumannii and Pseudomonas aeruginosa from New York City. J. Antimicrob. Chemother..

[93] Mendes R.E., Farrell D.J., Flamm R.K., Talbot G.H., Ivezic-Schoenfeld Z., Paukner S., Sader H.S. (2016). In Vitro Activity of Lefamulin Tested against Streptococcus pneumoniae with Defined Serotypes, Including Multidrug-Resistant Isolates Causing Lower Respiratory Tract Infections in the United States. Antimicrob. Agents Chemother..

[94] Waites K.B., Crabb D.M., Duffy L.B., Jensen J.S., Liu Y., Paukner S. (2017). In Vitro Activities of Lefamulin and Other Antimicrobial Agents against Macrolide-Susceptible and Macrolide-Resistant Mycoplasma pneumoniae from the United States, Europe, and China. Antimicrob. Agents Chemother..

[95] Jacobsson S., Paukner S., Golparian D., Jensen J.S., Unemo M. (2017). In Vitro Activity of the Novel Pleuromutilin Lefamulin (BC-3781) and Effect of Efflux Pump Inactivation on Multidrug-Resistant and Extensively Drug-Resistant Neisseria gonorrhoeae. Antimicrob. Agents Chemother..

[96] Paukner S., Gruss A., Jensen J.S. (2018). In Vitro Activity of Lefamulin against Sexually Transmitted Bacterial Pathogens. Antimicrob. Agents Chemother..

[97] Paukner S., Gelone S.P., Arends S.J.R., Flamm R.K., Sader H.S. (2019). Antibacterial Activity of Lefamulin against Pathogens Most Commonly Causing Community-Acquired Bacterial Pneumonia: SENTRY Antimicrobial Surveillance Program (2015–2016). Antimicrob. Agents Chemother..

[98] Waites K.B., Crabb D.M., Duffy L.B. (2009). Comparative in vitro susceptibilities of human mycoplasmas and ureaplasmas to a new investigational ketolide, CEM-101. Antimicrob. Agents Chemother..

[99] McGhee P., Clark C., Kosowska-Shick K.M., Nagai K., Dewasse B., Beachel L., Appelbaum P.C. (2010). In vitro activity of CEM-101 against Streptococcus pneumoniae and Streptococcus pyogenes with defined macrolide resistance mechanisms. Antimicrob. Agents Chemother..

[100] Roblin P.M., Kohlhoff S.A., Parker C., Hammerschlag M.R. (2010). In vitro activity of CEM-101, a new fluoroketolide antibiotic, against Chlamydia trachomatis and Chlamydia (Chlamydophila) pneumoniae. Antimicrob. Agents Chemother..

[101] Woosley L.N., Castanheira M., Jones R.N. (2010). CEM-101 activity against Gram-positive organisms. Antimicrob. Agents Chemother..

[102] Farrell D.J., Sader H.S., Castanheira M., Biedenbach D.J., Rhomberg P.R., Jones R.N. (2010). Antimicrobial characterisation of CEM-101 activity against respiratory tract pathogens, including multidrug-resistant pneumococcal serogroup 19A isolates. Int. J. Antimicrob. Agents.

[103] Biedenbach D.J., Castanheira M., Jones R.N. (2010). Determination of CEM-101 activity tested against clinical isolates of Neisseria meningitidis from a worldwide collection. Antimicrob. Agents Chemother..

[104] Llano-Sotelo B., Dunkle J., Klepacki D., Zhang W., Fernandes P., Cate J.H., Mankin A.S. (2010). Binding and action of CEM-101, a new fluoroketolide antibiotic that inhibits protein synthesis. Antimicrob. Agents Chemother..

[105] Golparian D., Fernandes P., Ohnishi M., Jensen J.S., Unemo M. (2012). In vitro activity of the new fluoroketolide solithromycin (CEM-101) against a large collection of clinical Neisseria gonorrhoeae isolates and international reference strains, including those with high-level antimicrobial resistance: Potential treatment option for gonorrhea?. Antimicrob. Agents Chemother..

[106] Oldach D., Clark K., Schranz J., Das A., Craft J.C., Scott D., Jamieson B.D., Fernandes P. (2013). Randomized, double-blind, multicenter phase 2 study comparing the efficacy and safety of oral solithromycin (CEM-101) to those of oral levofloxacin in the treatment of patients with community-acquired bacterial pneumonia. Antimicrob. Agents Chemother..

[107] Mallegol J., Fernandes P., Melano R.G., Guyard C. (2014). Antimicrobial activity of solithromycin against clinical isolates of Legionella pneumophila serogroup 1. Antimicrob. Agents Chemother..

[108] Hook E.W., Golden M., Jamieson B.D., Dixon P.B., Harbison H.S., Lowens S., Fernandes P. (2015). A Phase 2 Trial of Oral Solithromycin 1200 mg or 1000 mg as Single-Dose Oral Therapy for Uncomplicated Gonorrhea. Clin. Infect. Dis..

[109] Hardy D.J., Vicino D., Fernandes P. (2016). In Vitro Activity of Solithromycin against Bordetella pertussis, an Emerging Respiratory Pathogen. Antimicrob. Agents Chemother..

[110] Lemaire S., Van Bambeke F., Tulkens P.M. (2009). Cellular accumulation and pharmacodynamic evaluation of the intracellular activity of CEM-101, a novel fluoroketolide, against *Staphylococcus aureus*, Listeria monocytogenes, and Legionella pneumophila in human THP-1 macrophages. Antimicrob. Agents Chemother..

[111] Barrera C.M., Mykietiuk A., Metev H., Nitu M.F., Karimjee N., Doreski P.A., Mitha I., Tanaseanu C.M., Molina J.M., Antonovsky Y. (2016). Efficacy and safety of oral solithromycin versus oral moxifloxacin for treatment of community-acquired bacterial pneumonia: A global, double-blind, multicentre, randomised, active-controlled, non-inferiority trial (SOLITAIRE-ORAL). Lancet Infect. Dis..

[112] File T.M., Rewerska B., Vucinic-Mihailovic V., Gonong J.R.V., Das A.F., Keedy K., Taylor D., Sheets A., Fernandes P., Oldach D. (2016). SOLITAIRE-IV: A Randomized, Double-Blind, Multicenter Study Comparing the Efficacy and Safety of Intravenous-to-Oral Solithromycin to Intravenous-to-Oral Moxifloxacin for Treatment of Community-Acquired Bacterial Pneumonia. Clin. Infect. Dis..

[113] Pankuch G.A., Kelly L.M., Lin G., Bryskier A., Couturier C., Jacobs M.R., Appelbaum P.C. (2003). Activities of a new oral streptogramin, XRP 2868, compared to those of other agents against Streptococcus pneumoniae and haemophilus species. Antimicrob. Agents Chemother..

[114] Goldstein E.J., Citron D.M., Merriam C.V., Warren Y.A., Tyrrell K.L., Fernandez H.T., Bryskier A. (2005). Comparative in vitro activities of XRP 2868, pristinamycin, quinupristin-dalfopristin, vancomycin, daptomycin, linezolid, clarithromycin, telithromycin, clindamycin, and ampicillin against anaerobic gram-positive species, actinomycetes, and lactobacilli. Antimicrob. Agents Chemother..

[115] Dupuis M., Leclercq R. (2006). Activity of a new oral streptogramin, XRP2868, against gram-positive cocci harboring various mechanisms of resistance to streptogramins. Antimicrob. Agents Chemother..

[116] Pankuch G.A., Lin G., Clark C., Appelbaum P.C. (2011). Time-kill activity of the streptogramin NXL 103 against Gram-positive and -negative bacteria. Antimicrob. Agents Chemother..

[117] Schneider P., Hawser S., Islam K. (2003). Iclaprim, a novel diaminopyrimidine with potent activity on trimethoprim sensitive and resistant bacteria. Bioorg Med. Chem. Lett..

[118] Kohlhoff S.A., Roblin P.M., Reznik T., Hawser S., Islam K., Hammerschlag M.R. (2004). In vitro activity of a novel diaminopyrimidine compound, iclaprim, against Chlamydia trachomatis and C. pneumoniae. Antimicrob. Agents Chemother..

[119] Morrissey I., Hawser S. (2007). Activity of iclaprim against *Legionella pneumophila*. J. Antimicrob. Chemother..

[120] Morrissey I., Maher K., Hawser S. (2009). Activity of iclaprim against clinical isolates of *Streptococcus pyogenes* and *Streptococcus agalactiae*. J. Antimicrob. Chemother..

[121] Zhanel G.G., Karlowsky J.A. (2009). In vitro activity of Iclaprim against respiratory and bacteremic isolates of Streptococcus pneumoniae. Antimicrob. Agents Chemother..

[122] Krievins D., Brandt R., Hawser S., Hadvary P., Islam K. (2009). Multicenter, randomized study of the efficacy and safety of intravenous iclaprim in complicated skin and skin structure infections. Antimicrob. Agents Chemother..

[123] Holland T.L., O’Riordan W., McManus A., Shin E., Borghei A., File T.M., Wilcox M.H., Torres A., Dryden M., Lodise T. (2018). A Phase 3, Randomized, Double-Blind, Multicenter Study To Evaluate the Safety and Efficacy of Intravenous Iclaprim versus Vancomycin for Treatment of Acute Bacterial Skin and Skin Structure Infections Suspected or Confirmed To Be Due to Gram-Positive Pathogens (REVIVE-2 Study). Antimicrob. Agents Chemother..

[124] Huang D.B. (2019). Extracellular and intracellular activity of iclaprim against Listeria monocytogenes. Int. J. Antimicrob. Agents.

[125] Huang D.B., O’Riordan W., Overcash J.S., Heller B., Amin F., File T.M., Wilcox M.H., Torres A., Dryden M., Holland T.L. (2018). A Phase 3, Randomized, Double-Blind, Multicenter Study to Evaluate the Safety and Efficacy of Intravenous Iclaprim Vs Vancomycin for the Treatment of Acute Bacterial Skin and Skin Structure Infections Suspected or Confirmed to be Due to Gram-Positive Pathogens: REVIVE-1. Clin. Infect. Dis..

[126] Wang L., Belisle B., Bassiri M., Xu P., Debabov D., Celeri C., Alvarez N., Robson M.C., Payne W.G., Najafi R. (2011). Chemical characterization and biological properties of NVC-422, a novel, stable N-chlorotaurine analog. Antimicrob. Agents Chemother..

[127] Martini C., Hammerer-Lercher A., Zuck M., Jekle A., Debabov D., Anderson M., Nagl M. (2012). Antimicrobial and anticoagulant activities of N-chlorotaurine, N,N-dichloro-2,2-dimethyltaurine, and N-monochloro-2,2-dimethyltaurine in human blood. Antimicrob. Agents Chemother..

[128] D’Lima L., Friedman L., Wang L., Xu P., Anderson M., Debabov D. (2012). No decrease in susceptibility to NVC-422 in multiple-passage studies with methicillin-resistant *Staphylococcus aureus*, S. aureus, Pseudomonas aeruginosa, and Escherichia coli. Antimicrob. Agents Chemother..

[129] Jekle A., Yoon J., Zuck M., Najafi R., Wang L., Shiau T., Francavilla C., Rani S.A., Eitzinger C., Nagl M. (2013). NVC-422 inactivates *Staphylococcus aureus* toxins. Antimicrob. Agents Chemother..

[130] Iovino S.M., Krantz K.D., Blanco D.M., Fernández J.A., Ocampo N., Najafi A., Memarzadeh B., Celeri C., Debabov D., Khosrovi B. (2011). NVC-422 topical gel for the treatment of impetigo. Int. J. Clin. Exp. Pathol..

[131] Nilsson A.C., Janson H., Wold H., Fugelli A., Andersson K., Håkangård C., Olsson P., Olsen W.M. (2015). LTX-109 is a novel agent for nasal decolonization of methicillin-resistant and -sensitive *Staphylococcus aureus*. Antimicrob. Agents Chemother..

[132] Saravolatz L.D., Pawlak J., Johnson L., Bonilla H., Saravolatz L.D., Fakih M.G., Fugelli A., Olsen W.M. (2012). In vitro activities of LTX-109, a synthetic antimicrobial peptide, against methicillin-resistant, vancomycin-intermediate, vancomycin-resistant, daptomycin-nonsusceptible, and linezolid-nonsusceptible *Staphylococcus aureus*. Antimicrob. Agents Chemother..

[133] Ma Z., He S., Yuan Y., Zhuang Z., Liu Y., Wang H., Chen J., Xu X., Ding C., Molodtsov V. (2022). Design, Synthesis, and Characterization of TNP-2198, a Dual-Targeted Rifamycin-Nitroimidazole Conjugate with Potent Activity against Microaerophilic and Anaerobic Bacterial Pathogens. J. Med. Chem..

[134] Li X., Liu Y., Wang M., Gao L., Liu J., Zhang H., Wu M., Chen H., Lou J., Wang J. (2024). Safety, pharmacokinetics, and efficacy of rifasutenizol, a novel dual-targeted antibacterial agent in healthy participants and patients in China with Helicobacter pylori infection: Four randomised clinical trials. Lancet Infect. Dis..

[135] Song Z., Zhou L., Wang W., Lan C., Tang T., Xie J., Fan H., Wang X., Zuo X., Zhu Y. (2026). Rifasutenizol-based triple therapy versus bismuth plus clarithromycin-based triple therapy for first-line treatment of Helicobacter pylori infection in China (EVEREST-HP): A phase 3, multicentre, randomised, triple-dummy, double-blind, controlled, non-inferiority trial. Lancet Infect. Dis..

[136] Candiani G., Abbondi M., Borgonovi M., Romanò G., Parenti F. (1999). In-vitro and in-vivo antibacterial activity of BI 397, a new semi-synthetic glycopeptide antibiotic. J. Antimicrob. Chemother..

[137] Goldstein E.J., Citron D.M., Merriam C.V., Warren Y., Tyrrell K., Fernandez H.T. (2003). In vitro activities of dalbavancin and nine comparator agents against anaerobic gram-positive species and corynebacteria. Antimicrob. Agents Chemother..

[138] Seltzer E., Dorr M.B., Goldstein B.P., Perry M., Dowell J.A., Henkel T. (2003). Once-weekly dalbavancin versus standard-of-care antimicrobial regimens for treatment of skin and soft-tissue infections. Clin. Infect. Dis..

[139] Lin G., Credito K., Ednie L.M., Appelbaum P.C. (2005). Antistaphylococcal activity of dalbavancin, an experimental glycopeptide. Antimicrob. Agents Chemother..

[140] Jauregui L.E., Babazadeh S., Seltzer E., Goldberg L., Krievins D., Frederick M., Krause D., Satilovs I., Endzinas Z., Breaux J. (2005). Randomized, double-blind comparison of once-weekly dalbavancin versus twice-daily linezolid therapy for the treatment of complicated skin and skin structure infections. Clin. Infect. Dis..

[141] Lin G., Smith K., Ednie L.M., Appelbaum P.C. (2005). Antipneumococcal activity of dalbavancin compared to other agents. Antimicrob. Agents Chemother..

[142] Nord C.E., Rasmanis G., Wahlund E. (2006). Effect of dalbavancin on the normal intestinal microflora. J. Antimicrob. Chemother..

[143] Goldstein B.P., Draghi D.C., Sheehan D.J., Hogan P., Sahm D.F. (2007). Bactericidal activity and resistance development profiling of dalbavancin. Antimicrob. Agents Chemother..

[144] Biedenbach D.J., Bell J.M., Sader H.S., Turnidge J.D., Jones R.N. (2009). Activities of dalbavancin against a worldwide collection of 81,673 gram-positive bacterial isolates. Antimicrob. Agents Chemother..

[145] Heine H.S., Purcell B.K., Bassett J., Miller L., Goldstein B.P. (2010). Activity of dalbavancin against Bacillus anthracis in vitro and in a mouse inhalation anthrax model. Antimicrob. Agents Chemother..

[146] Hobdey S.E., Katahira E.J., Dockstader P., Davidson S.M., Bond L., Bolz D.D., Bryant A.E., Stevens D.L. (2017). Subinhibitory Dalbavancin Attenuates Exotoxin Production from Methicillin-Sensitive and Methicillin-Resistant *Staphylococcus aureus* In Vitro. Antimicrob. Agents Chemother..

[147] Morata L., Cobo J., Fernández-Sampedro M., Guisado Vasco P., Ruano E., Lora-Tamayo J., Sánchez Somolinos M., González Ruano P., Rico Nieto A., Arnaiz A. (2019). Safety and Efficacy of Prolonged Use of Dalbavancin in Bone and Joint Infections. Antimicrob. Agents Chemother..

[148] Hugg R., Stegger M., Söderquist B. (2026). Emerging resistance in staphylococci following long-term dalbavancin treatment for prosthetic joint infections. J. Antimicrob. Chemother..

[149] Gonzalez D., Bradley J.S., Blumer J., Yogev R., Watt K.M., James L.P., Palazzi D.L., Bhatt-Mehta V., Sullivan J.E., Zhang L. (2017). Dalbavancin Pharmacokinetics and Safety in Children 3 Months to 11 Years of Age. Pediatr. Infect. Dis. J..

[150] Cooper R.D., Snyder N.J., Zweifel M.J., Staszak M.A., Wilkie S.C., Nicas T.I., Mullen D.L., Butler T.F., Rodriguez M.J., Huff B.E. (1996). Reductive alkylation of glycopeptide antibiotics: Synthesis and antibacterial activity. J. Antibiot..

[151] Schwalbe R.S., McIntosh A.C., Qaiyumi S., Johnson J.A., Johnson R.J., Furness K.M., Holloway W.J., Steele-Moore L. (1996). In vitro activity of LY333328, an investigational glycopeptide antibiotic, against enterococci and staphylococci. Antimicrob. Agents Chemother..

[152] Fasola E., Spangler S.K., Ednie L.M., Jacobs M.R., Bajaksouzian S., Appelbaum P.C. (1996). Comparative activities of LY 333328, a new glycopeptide, against penicillin-susceptible and -resistant pneumococci. Antimicrob. Agents Chemother..

[153] Jones R.N., Barrett M.S., Erwin M.E. (1997). In vitro activity and spectrum of LY333328, a novel glycopeptide derivative. Antimicrob. Agents Chemother..

[154] Biavasco F., Vignaroli C., Lupidi R., Manso E., Facinelli B., Varaldo P.E. (1997). In vitro antibacterial activity of LY333328, a new semisynthetic glycopeptide. Antimicrob. Agents Chemother..

[155] O’Connor R., Baines S.D., Freeman J., Wilcox M.H. (2008). In vitro susceptibility of genotypically distinct and clonal Clostridium difficile strains to oritavancin. J. Antimicrob. Chemother..

[156] Heine H.S., Bassett J., Miller L., Bassett A., Ivins B.E., Lehoux D., Arhin F.F., Parr T.R., Moeck G. (2008). Efficacy of oritavancin in a murine model of Bacillus anthracis spore inhalation anthrax. Antimicrob. Agents Chemother..

[157] Corey G.R., Kabler H., Mehra P., Gupta S., Overcash J.S., Porwal A., Giordano P., Lucasti C., Perez A., Good S. (2014). Single-dose oritavancin in the treatment of acute bacterial skin infections. N. Engl. J. Med..

[158] Stryjewski M.E., Potgieter P.D., Li Y.P., Barriere S.L., Churukian A., Kingsley J., Corey G.R. (2012). TD-1792 versus vancomycin for treatment of complicated skin and skin structure infections. Antimicrob. Agents Chemother..

[159] Leuthner K.D., Vidaillac C., Cheung C.M., Rybak M.J. (2010). In vitro activity of the new multivalent glycopeptide-cephalosporin antibiotic TD-1792 against vancomycin-nonsusceptible Staphylococcus isolates. Antimicrob. Agents Chemother..

[160] Long D.D., Aggen J.B., Chinn J., Choi S.K., Christensen B.G., Fatheree P.R., Green D., Hegde S.S., Judice J.K., Kaniga K. (2008). Exploring the positional attachment of glycopeptide/beta-lactam heterodimers. J. Antibiot..

[161] Blais J., Lewis S.R., Krause K.M., Benton B.M. (2012). Antistaphylococcal activity of TD-1792, a multivalent glycopeptide-cephalosporin antibiotic. Antimicrob. Agents Chemother..

[162] Tyrrell K.L., Citron D.M., Warren Y.A., Goldstein E.J. (2012). In vitro activity of TD-1792, a multivalent glycopeptide-cephalosporin antibiotic, against 377 strains of anaerobic bacteria and 34 strains of Corynebacterium species. Antimicrob. Agents Chemother..

[163] Vera-Cabrera L., Gonzalez E., Rendon A., Ocampo-Candiani J., Welsh O., Velazquez-Moreno V.M., Choi S.H., Molina-Torres C. (2006). In vitro activities of DA-7157 and DA-7218 against Mycobacterium tuberculosis and Nocardia brasiliensis. Antimicrob. Agents Chemother..

[164] Vera-Cabrera L., Brown-Elliott B.A., Wallace R.J., Ocampo-Candiani J., Welsh O., Choi S.H., Molina-Torres C.A. (2006). In vitro activities of the novel oxazolidinones DA-7867 and DA-7157 against rapidly and slowly growing mycobacteria. Antimicrob. Agents Chemother..

[165] Shaw K.J., Poppe S., Schaadt R., Brown-Driver V., Finn J., Pillar C.M., Shinabarger D., Zurenko G. (2008). In vitro activity of TR-700, the antibacterial moiety of the prodrug TR-701, against linezolid-resistant strains. Antimicrob. Agents Chemother..

[166] Prokocimer P., Bien P., Surber J., Mehra P., DeAnda C., Bulitta J.B., Corey G.R. (2011). Phase 2, randomized, double-blind, dose-ranging study evaluating the safety, tolerability, population pharmacokinetics, and efficacy of oral torezolid phosphate in patients with complicated skin and skin structure infections. Antimicrob. Agents Chemother..

[167] Choi S., Im W., Bartizal K. (2012). Activity of tedizolid phosphate (TR-701) in murine models of infection with penicillin-resistant and penicillin-sensitive *Streptococcus pneumoniae*. Antimicrob. Agents Chemother..

[168] Goldstein E.J., Citron D.M., Tyrrell K.L., Leoncio E.S., Merriam C.V. (2017). The underappreciated in vitro activity of tedizolid against Bacteroides fragilis species, including strains resistant to metronidazole and carbapenems. Anaerobe.

[169] Wunderink R.G., Roquilly A., Croce M., Rodriguez Gonzalez D., Fujimi S., Butterton J.R., Broyde N., Popejoy M.W., Kim J.Y., De Anda C. (2021). A Phase 3, Randomized, Double-Blind Study Comparing Tedizolid Phosphate and Linezolid for Treatment of Ventilated Gram-Positive Hospital-Acquired or Ventilator-Associated Bacterial Pneumonia. Clin. Infect. Dis..

[170] Locher H.H., Seiler P., Chen X., Schroeder S., Pfaff P., Enderlin M., Klenk A., Fournier E., Hubschwerlen C., Ritz D. (2014). In vitro and in vivo antibacterial evaluation of cadazolid, a new antibiotic for treatment of Clostridium difficile infections. Antimicrob. Agents Chemother..

[171] Locher H.H., Caspers P., Bruyère T., Schroeder S., Pfaff P., Knezevic A., Keck W., Ritz D. (2014). Investigations of the mode of action and resistance development of cadazolid, a new antibiotic for treatment of Clostridium difficile infections. Antimicrob. Agents Chemother..

[172] Chilton C.H., Crowther G.S., Baines S.D., Todhunter S.L., Freeman J., Locher H.H., Athanasiou A., Wilcox M.H. (2014). In vitro activity of cadazolid against clinically relevant *Clostridium difficile* isolates and in an in vitro gut model of *C. difficile* infection. J. Antimicrob. Chemother..

[173] Gerding D.N., Cornely O.A., Grill S., Kracker H., Marrast A.C., Nord C.E., Talbot G.H., Buitrago M., Gheorghe Diaconescu I., Murta de Oliveira C. (2019). Cadazolid for the treatment of Clostridium difficile infection: Results of two double-blind, placebo-controlled, non-inferiority, randomised phase 3 trials. Lancet Infect. Dis..

[174] Louie T., Nord C.E., Talbot G.H., Wilcox M., Gerding D.N., Buitrago M., Kracker H., Charef P., Cornely O.A. (2015). Multicenter, Double-Blind, Randomized, Phase 2 Study Evaluating the Novel Antibiotic Cadazolid in Patients with *Clostridium difficile* Infection. Antimicrob. Agents Chemother..

[175] Pfaller M.A., Castanheira M., Sader H.S., Jones R.N. (2010). Evaluation of the activity of fusidic acid tested against contemporary Gram-positive clinical isolates from the USA and Canada. Int. J. Antimicrob. Agents.

[176] Jones R.N., Biedenbach D.J., Roblin P.M., Kohlhoff S.A., Hammerschlag M.R. (2010). Update on fusidic acid (CEM-102) tested against Neisseria gonorrhoeae and Chlamydia trachomatis. Antimicrob. Agents Chemother..

[177] Craft J.C., Moriarty S.R., Clark K., Scott D., Degenhardt T.P., Still J.G., Corey G.R., Das A., Fernandes P. (2011). A randomized, double-blind phase 2 study comparing the efficacy and safety of an oral fusidic acid loading-dose regimen to oral linezolid for the treatment of acute bacterial skin and skin structure infections. Clin. Infect. Dis..

[178] Sahm D.F., Deane J., Pillar C.M., Fernandes P. (2013). In vitro activity of CEM-102 (fusidic acid) against prevalent clones and resistant phenotypes of *Staphylococcus aureus*. Antimicrob. Agents Chemother..

[179] Pushkin R., Iglesias-Ussel M.D., Keedy K., MacLauchlin C., Mould D.R., Berkowitz R., Kreuzer S., Darouiche R., Oldach D., Fernandes P. (2016). A Randomized Study Evaluating Oral Fusidic Acid (CEM-102) in Combination With Oral Rifampin Compared With Standard-of-Care Antibiotics for Treatment of Prosthetic Joint Infections: A Newly Identified Drug–Drug Interaction. Clin. Infect. Dis..

[180] Citron D.M., Warren Y.A., Tyrrell K.L., Merriam V., Goldstein E.J. (2009). Comparative in vitro activity of REP3123 against Clostridium difficile and other anaerobic intestinal bacteria. J. Antimicrob. Chemother..

[181] Ochsner U.A., Bell S.J., O’Leary A.L., Hoang T., Stone K.C., Young C.L., Critchley I.A., Janjic N. (2009). Inhibitory effect of REP3123 on toxin and spore formation in Clostridium difficile, and in vivo efficacy in a hamster gastrointestinal infection model. J. Antimicrob. Chemother..

[182] Critchley I.A., Green L.S., Young C.L., Bullard J.M., Evans R.J., Price M., Jarvis T.C., Guiles J.W., Janjic N., Ochsner U.A. (2009). Spectrum of activity and mode of action of REP3123, a new antibiotic to treat Clostridium difficile infections. J. Antimicrob. Chemother..

[183] Lomeli B.K., Galbraith H., Schettler J., Saviolakis G.A., El-Amin W., Osborn B., Ravel J., Hazleton K., Lozupone C.A., Evans R.J. (2019). Multiple-Ascending-Dose Phase 1 Clinical Study of the Safety, Tolerability, and Pharmacokinetics of CRS3123, a Narrow-Spectrum Agent with Minimal Disruption of Normal Gut Microbiota. Antimicrob. Agents Chemother..

[184] Louie T., Ribble W., Boccumini L., Johnson K., De Groote M.A., Day J., Mason C., Sun X., Freeman J., Gu K. (2026). Safety and efficacy of CRS3123 in adults with a primary episode or first recurrence of Clostridioides difficile infection: A phase 2, randomised, double-blind, multicentre, vancomycin-controlled study. Lancet Infect. Dis..

[185] Nayak S.U., Griffiss J.M., Blumer J., O’Riordan M.A., Gray W., McKenzie R., Jurao R.A., An A.T., Le M., Bell S.J. (2017). Safety, Tolerability, Systemic Exposure, and Metabolism of CRS3123, a Methionyl-tRNA Synthetase Inhibitor Developed for Treatment of Clostridium difficile, in a Phase 1 Study. Antimicrob. Agents Chemother..

[186] Dvoskin S., Xu W.C., Brown N.C., Yanachkov I.B., Yanachkova M., Wright G.E. (2012). A novel agent effective against *Clostridium difficile* infection. Antimicrob. Agents Chemother..

[187] Murray B., Wolfe C., Marra A., Pillar C., Shinabarger D. (2020). In vitro activity of the novel antibacterial agent ibezapolstat (ACX-362E) against *Clostridioides difficile*. J. Antimicrob. Chemother..

[188] Garey K.W., McPherson J., Dinh A.Q., Hu C., Jo J., Wang W., Lancaster C.K., Gonzales-Luna A.J., Loveall C., Begum K. (2022). Efficacy, Safety, Pharmacokinetics, and Microbiome Changes of Ibezapolstat in Adults with *Clostridioides difficile* Infection: A Phase 2a Multicenter Clinical Trial. Clin. Infect. Dis..

[189] Bassères E., Eubank T.A., Begum K., Alam M.J., Jo J., Le T.M., Lancaster C.K., Gonzales-Luna A.J., Garey K.W. (2024). Antibacterial activity of ibezapolstat against antimicrobial-resistant clinical strains of *Clostridioides difficile*. Antimicrob. Agents Chemother..

[190] Eubank T.A., Jo J., Alam M.J., Begum K., McPherson J.K., Le T.M., Horvath T.D., Haidacher S.J., Poggio E.C., Lin R. (2025). Efficacy, safety, pharmacokinetics, and associated microbiome changes of ibezapolstat compared with vancomycin in adults with *Clostridioides difficile* infection: A phase 2b, randomised, double-blind, active-controlled, multicentre study. Lancet Microbe.

[191] Wolfe T.M., Jo J., Pinkham N.V., Garey K.W., Walk S.T. (2025). The impact of ibezapolstat and other *Clostridioides difficile* infection-relevant antibiotics on the microbiome of humanized mice. Antimicrob. Agents Chemother..

[192] Vandell A.G., Inoue S., Dennie J., Nagasawa Y., Gajee R., Pav J., Zhang G., Zamora C., Masuda N., Senaldi G. (2018). Phase 1 Study To Assess the Safety, Tolerability, Pharmacokinetics, and Pharmacodynamics of Multiple Oral Doses of DS-2969b, a Novel GyrB Inhibitor, in Healthy Subjects. Antimicrob. Agents Chemother..

[193] Mathur T., Barman T.K., Kumar M., Singh D., Kumar R., Khera M.K., Yamada M., Inoue S.I., Upadhyay D.J., Masuda N. (2018). In Vitro and In Vivo Activities of DS-2969b, a Novel GyrB Inhibitor, against *Clostridium difficile*. Antimicrob. Agents Chemother..

[194] Barman T.K., Kumar M., Mathur T., Namba E., Singh D., Chaira T., Kurosaka Y., Yamada M., Upadhyay D.J., Masuda N. (2018). In Vitro and In Vivo Activities of DS-2969b, a Novel GyrB Inhibitor, and Its Water-Soluble Prodrug, DS11960558, against Methicillin-Resistant *Staphylococcus aureus*. Antimicrob. Agents Chemother..

[195] Vickers R., Robinson N., Best E., Echols R., Tillotson G., Wilcox M. (2015). A randomised phase 1 study to investigate safety, pharmacokinetics and impact on gut microbiota following single and multiple oral doses in healthy male subjects of SMT19969, a novel agent for Clostridium difficile infections. BMC Infect. Dis..

[196] Goldstein E.J., Citron D.M., Tyrrell K.L., Merriam C.V. (2013). Comparative in vitro activities of SMT19969, a new antimicrobial agent, against Clostridium difficile and 350 gram-positive and gram-negative aerobic and anaerobic intestinal flora isolates. Antimicrob. Agents Chemother..

[197] Weiss W., Pulse M., Vickers R. (2014). In vivo assessment of SMT19969 in a hamster model of clostridium difficile infection. Antimicrob. Agents Chemother..

[198] Bassères E., Endres B.T., Khaleduzzaman M., Miraftabi F., Alam M.J., Vickers R.J., Garey K.W. (2016). Impact on toxin production and cell morphology in *Clostridium difficile* by ridinilazole (SMT19969), a novel treatment for *C. difficile* infection. J. Antimicrob. Chemother..

[199] Vickers R.J., Tillotson G.S., Nathan R., Hazan S., Pullman J., Lucasti C., Deck K., Yacyshyn B., Maliakkal B., Pesant Y. (2017). Efficacy and safety of ridinilazole compared with vancomycin for the treatment of Clostridium difficile infection: A phase 2, randomised, double-blind, active-controlled, non-inferiority study. Lancet Infect. Dis..

[200] Mason C.S., Avis T., Hu C., Nagalingam N., Mudaliar M., Coward C., Begum K., Gajewski K., Alam M.J., Bassères E. (2023). The Novel DNA Binding Mechanism of Ridinilazole, a Precision Clostridiodes difficile Antibiotic. Antimicrob. Agents Chemother..

[201] Okhuysen P.C., Ramesh M.S., Louie T., Kiknadze N., Torre-Cisneros J., de Oliveira C.M., Van Steenkiste C., Stychneuskaya A., Garey K.W., Garcia-Diaz J. (2024). A Randomized, Double-Blind, Phase 3 Safety and Efficacy Study of Ridinilazole Versus Vancomycin for Treatment of *Clostridioides difficile* Infection: Clinical Outcomes With Microbiome and Metabolome Correlates of Response. Clin. Infect. Dis..

[202] Ackermann G., Löffler B., Adler D., Rodloff A.C. (2004). In vitro activity of OPT-80 against Clostridium difficile. Antimicrob. Agents Chemother..

[203] Credito K.L., Appelbaum P.C. (2004). Activity of OPT-80, a novel macrocycle, compared with those of eight other agents against selected anaerobic species. Antimicrob. Agents Chemother..

[204] Finegold S.M., Molitoris D., Vaisanen M.L., Song Y., Liu C., Bolaños M. (2004). In vitro activities of OPT-80 and comparator drugs against intestinal bacteria. Antimicrob. Agents Chemother..

[205] Karlowsky J.A., Laing N.M., Zhanel G.G. (2008). In vitro activity of OPT-80 tested against clinical isolates of toxin-producing Clostridium difficile. Antimicrob. Agents Chemother..

[206] Louie T.J., Emery J., Krulicki W., Byrne B., Mah M. (2009). OPT-80 eliminates Clostridium difficile and is sparing of bacteroides species during treatment of C. difficile infection. Antimicrob. Agents Chemother..

[207] Louie T., Miller M., Donskey C., Mullane K., Goldstein E.J. (2009). Clinical outcomes, safety, and pharmacokinetics of OPT-80 in a phase 2 trial with patients with Clostridium difficile infection. Antimicrob. Agents Chemother..

[208] Louie T.J., Miller M.A., Mullane K.M., Weiss K., Lentnek A., Golan Y., Gorbach S., Sears P., Shue Y.K. (2011). Fidaxomicin versus vancomycin for Clostridium difficile infection. N. Engl. J. Med..

[209] Louie T.J., Cannon K., Byrne B., Emery J., Ward L., Eyben M., Krulicki W. (2012). Fidaxomicin preserves the intestinal microbiome during and after treatment of Clostridium difficile infection (CDI) and reduces both toxin reexpression and recurrence of CDI. Clin. Infect. Dis..

[210] Babakhani F., Bouillaut L., Gomez A., Sears P., Nguyen L., Sonenshein A.L. (2012). Fidaxomicin inhibits spore production in Clostridium difficile. Clin. Infect. Dis..

[211] Babakhani F., Bouillaut L., Sears P., Sims C., Gomez A., Sonenshein A.L. (2013). Fidaxomicin inhibits toxin production in Clostridium difficile. J. Antimicrob. Chemother..

[212] Smeltzer S., Hassoun A. (2013). Successful use of fidaxomicin in recurrent Clostridium difficile infection in a child. J. Antimicrob. Chemother..

[213] James G.A., Chesnel L., Boegli L., deLancey Pulcini E., Fisher S., Stewart P.S. (2018). Analysis of Clostridium difficile biofilms: Imaging and antimicrobial treatment. J. Antimicrob. Chemother..

[214] Ajami N.J., Cope J.L., Wong M.C., Petrosino J.F., Chesnel L. (2018). Impact of Oral Fidaxomicin Administration on the Intestinal Microbiota and Susceptibility to Clostridium difficile Colonization in Mice. Antimicrob. Agents Chemother..

[215] Wolf J., Kalocsai K., Fortuny C., Lazar S., Bosis S., Korczowski B., Petit A., Bradford D., Croos-Dabrera R., Incera E. (2020). Safety and Efficacy of Fidaxomicin and Vancomycin in Children and Adolescents with Clostridioides (Clostridium) difficile Infection: A Phase 3, Multicenter, Randomized, Single-blind Clinical Trial (SUNSHINE). Clin. Infect. Dis..

[216] Cao X., Boyaci H., Chen J., Bao Y., Landick R., Campbell E.A. (2022). Basis of narrow-spectrum activity of fidaxomicin on *Clostridioides difficile*. Nature.

[217] Citron D.M., Tyrrell K.L., Merriam C.V., Goldstein E.J. (2012). In vitro activities of CB-183,315, vancomycin, and metronidazole against 556 strains of Clostridium difficile, 445 other intestinal anaerobes, and 56 Enterobacteriaceae species. Antimicrob. Agents Chemother..

[218] Snydman D.R., Jacobus N.V., McDermott L.A. (2012). Activity of a novel cyclic lipopeptide, CB-183,315, against resistant Clostridium difficile and other Gram-positive aerobic and anaerobic intestinal pathogens. Antimicrob. Agents Chemother..

[219] Mascio C.T., Mortin L.I., Howland K.T., Van Praagh A.D., Zhang S., Arya A., Chuong C.L., Kang C., Li T., Silverman J.A. (2012). In vitro and in vivo characterization of CB-183,315, a novel lipopeptide antibiotic for treatment of Clostridium difficile. Antimicrob. Agents Chemother..

[220] Mascio C.T., Chesnel L., Thorne G., Silverman J.A. (2014). Surotomycin demonstrates low in vitro frequency of resistance and rapid bactericidal activity in *Clostridium difficile*, *Enterococcus faecalis*, and *Enterococcus faecium*. Antimicrob. Agents Chemother..

[221] Citron D.M., Tyrrell K.L., Dale S.E., Chesnel L., Goldstein E.J. (2016). Impact of Surotomycin on the Gut Microbiota of Healthy Volunteers in a Phase 1 Clinical Trial. Antimicrob. Agents Chemother..

[222] Endres B.T., Bassères E., Khaleduzzaman M., Alam M.J., Chesnel L., Garey K.W. (2016). Evaluating the Effects of Surotomycin Treatment on Clostridium difficile Toxin A and B Production, Immune Response, and Morphological Changes. Antimicrob. Agents Chemother..

[223] Daley P., Louie T., Lutz J.E., Khanna S., Stoutenburgh U., Jin M., Adedoyin A., Chesnel L., Guris D., Larson K.B. (2017). Surotomycin versus vancomycin in adults with Clostridium difficile infection: Primary clinical outcomes from the second pivotal, randomized, double-blind, Phase 3 trial. J. Antimicrob. Chemother..

[224] Lee C.H., Patino H., Stevens C., Rege S., Chesnel L., Louie T., Mullane K.M. (2016). Surotomycin versus vancomycin for Clostridium difficile infection: Phase 2, randomized, controlled, double-blind, non-inferiority, multicentre trial. J. Antimicrob. Chemother..

[225] Cannon K., Byrne B., Happe J., Wu K., Ward L., Chesnel L., Louie T. (2017). Enteric microbiome profiles during a randomized Phase 2 clinical trial of surotomycin versus vancomycin for the treatment of Clostridium difficile infection. J. Antimicrob. Chemother..

[226] Ooi N., Miller K., Hobbs J., Rhys-Williams W., Love W., Chopra I. (2009). XF-73, a novel antistaphylococcal membrane-active agent with rapid bactericidal activity. J. Antimicrob. Chemother..

[227] Ooi N., Miller K., Randall C., Rhys-Williams W., Love W., Chopra I. (2010). XF-70 and XF-73, novel antibacterial agents active against slow-growing and non-dividing cultures of *Staphylococcus aureus* including biofilms. J. Antimicrob. Chemother..

[228] Yendewa G.A., Griffiss J.M., Jacobs M.R., Fulton S.A., O’Riordan M.A., Gray W.A., Proskin H.M., Winkle P., Salata R.A. (2020). A two-part phase 1 study to establish and compare the safety and local tolerability of two nasal formulations of XF-73 for decolonisation of *Staphylococcus aureus*: A previously investigated 0.5 mg/g viscosified gel formulation versus a modified formulation. J. Glob. Antimicrob. Resist..

[229] Farrell D.J., Robbins M., Rhys-Williams W., Love W.G. (2010). In vitro activity of XF-73, a novel antibacterial agent, against antibiotic-sensitive and -resistant Gram-positive and Gram-negative bacterial species. Int. J. Antimicrob. Agents.

[230] Mangino J.E., Firstenberg M.S., Milewski R.K.C., Rhys-Williams W., Lees J.P., Dane A., Love W.G., Gonzalez Moreno J. (2023). Exeporfinium chloride (XF-73) nasal gel dosed over 24 hours prior to surgery significantly reduced *Staphylococcus aureus* nasal carriage in cardiac surgery patients: Safety and efficacy results from a randomized placebo-controlled phase 2 study. Infect. Control Hosp. Epidemiol..

[231] Flamm R.K., Rhomberg P.R., Kaplan N., Jones R.N., Farrell D.J. (2015). Activity of Debio1452, a FabI inhibitor with potent activity against *Staphylococcus aureus* and coagulase-negative Staphylococcus spp., including multidrug-resistant strains. Antimicrob. Agents Chemother..

[232] Wittke F., Vincent C., Chen J., Heller B., Kabler H., Overcash J.S., Leylavergne F., Dieppois G. (2020). Afabicin, a First-in-Class Antistaphylococcal Antibiotic, in the Treatment of Acute Bacterial Skin and Skin Structure Infections: Clinical Noninferiority to Vancomycin/Linezolid. Antimicrob. Agents Chemother..

[233] Nowakowska J., Cameron D.R., De Martino A., Kühn J., Le Fresne-Languille S., Leuillet S., Amouzou Y., Wittke F., Carton T., Le Vacon F. (2023). Evaluation of the microbiota-sparing properties of the anti-staphylococcal antibiotic afabicin. J. Antimicrob. Chemother..

[234] Shamaei Tousi A., Marquardt M., Menetrey A., Nowakowska J., Dao J., Cameron D., Catala-Goldschmidt C., Chaves R. (2025). P-65. Results from A Phase 2 Clinical Trial for Treatment of Bone And Joint Infections with Afabicin, A First-in-Class Selective Anti-Staphylococcal Antibiotic. Open Forum Infect. Dis..

[235] Peck M., Rothenberg M.E., Deng R., Lewin-Koh N., She G., Kamath A.V., Carrasco-Triguero M., Saad O., Castro A., Teufel L. (2019). A Phase 1, Randomized, Single-Ascending-Dose Study To Investigate the Safety, Tolerability, and Pharmacokinetics of DSTA4637S, an Anti-*Staphylococcus aureus* Thiomab Antibody-Antibiotic Conjugate, in Healthy Volunteers. Antimicrob. Agents Chemother..

[236] Lim J., Lewin-Koh N., Chu T., Rymut S.M., Berhanu A., Carrasco-Triguero M., Rosenberger C.C., Hazenbos W.L., Miller L.G., Fowler V.G. (2020). 167. A Phase 1b, Randomized, Double-blind, Placebo-controlled, Multiple-ascending Dose Study to Investigate the Safety, Tolerability, and Pharmacokinetics of DSTA4637S in Patients with *Staphylococcus aureus* Bacteremia Receiving Standard-of-care Antibiotics. Open Forum Infect. Dis..

[237] Kitanaka S., Ikezawa T., Yasukawa K., Yamanouchi S., Takido M., Sung H.K., Kim I.H. (1990). (+)-Alpha-viniferin, an anti-inflammatory compound from Caragana chamlagu root. Chem. Pharm. Bull..

[238] Seo H., Kim M., Kim S., Mahmud H.A., Islam M.I., Nam K.W., Cho M.L., Kwon H.S., Song H.Y. (2017). In vitro activity of alpha-viniferin isolated from the roots of *Carex humilis* against *Mycobacterium tuberculosis*. Pulm. Pharmacol. Ther..

[239] Rahim M.A., Seo H., Kim S., Jeong Y.K., Tajdozian H., Kim M., Lee S., Song H.Y. (2021). A Clinical Trial to Evaluate the Efficacy of α-Viniferin in *Staphylococcus aureus*—Specific Decolonization without Depleting the Normal Microbiota of Nares. Pol. J. Microbiol..

[240] Cao Y., Huang Y., Xie J., Wu Q., Yang S., Yang X., Huang J., Liu J., Meng J., Yang G. (2025). Intrapulmonary penetration and pharmacokinetics of siderophore cephalosporin HRS-8427 injection in Chinese healthy subjects. Antimicrob. Agents Chemother..

[241] Wu Y., Han Q., Yang H., Xue J., Wang L., Mao X., Yin T., Jiang H., Xu S., Huang Y. (2026). Effects of renal impairment and hemodialysis on the pharmacokinetics and safety of HRS-8427, a siderophore cephalosporin for Gram-negative bacterial infections. Antimicrob. Agents Chemother..

[242] Yin T., Qu B., Zhang Q., Xu S., Wu Y., Jiang H., Huang Y., Hu W. (2026). Pharmacokinetics, safety, and tolerability of HRS-8427, a novel anti-carbapenem-resistant cephalosporin, in healthy subjects. Antimicrob. Agents Chemother..

[243] Kohira N., West J., Ito A., Ito-Horiyama T., Nakamura R., Sato T., Rittenhouse S., Tsuji M., Yamano Y. (2016). In Vitro Antimicrobial Activity of a Siderophore Cephalosporin, S-649266, against Enterobacteriaceae Clinical Isolates, Including Carbapenem-Resistant Strains. Antimicrob. Agents Chemother..

[244] Ito A., Kohira N., Bouchillon S.K., West J., Rittenhouse S., Sader H.S., Rhomberg P.R., Jones R.N., Yoshizawa H., Nakamura R. (2016). In vitro antimicrobial activity of S-649266, a catechol-substituted siderophore cephalosporin, when tested against non-fermenting Gram-negative bacteria. J. Antimicrob. Chemother..

[245] Ito A., Nishikawa T., Matsumoto S., Yoshizawa H., Sato T., Nakamura R., Tsuji M., Yamano Y. (2016). Siderophore Cephalosporin Cefiderocol Utilizes Ferric Iron Transporter Systems for Antibacterial Activity against Pseudomonas aeruginosa. Antimicrob. Agents Chemother..

[246] Hackel M.A., Tsuji M., Yamano Y., Echols R., Karlowsky J.A., Sahm D.F. (2017). In Vitro Activity of the Siderophore Cephalosporin, Cefiderocol, against a Recent Collection of Clinically Relevant Gram-Negative Bacilli from North America and Europe, Including Carbapenem-Nonsusceptible Isolates (SIDERO-WT-2014 Study). Antimicrob. Agents Chemother..

[247] Ito A., Sato T., Ota M., Takemura M., Nishikawa T., Toba S., Kohira N., Miyagawa S., Ishibashi N., Matsumoto S. (2018). In Vitro Antibacterial Properties of Cefiderocol, a Novel Siderophore Cephalosporin, against Gram-Negative Bacteria. Antimicrob. Agents Chemother..

[248] Jacobs M.R., Abdelhamed A.M., Good C.E., Rhoads D.D., Hujer K.M., Hujer A.M., Domitrovic T.N., Rudin S.D., Richter S.S., van Duin D. (2019). ARGONAUT-I: Activity of Cefiderocol (S-649266), a Siderophore Cephalosporin, against Gram-Negative Bacteria, Including Carbapenem-Resistant Nonfermenters and Enterobacteriaceae with Defined Extended-Spectrum β-Lactamases and Carbapenemases. Antimicrob. Agents Chemother..

[249] Portsmouth S., van Veenhuyzen D., Echols R., Machida M., Ferreira J.C.A., Ariyasu M., Tenke P., Nagata T.D. (2018). Cefiderocol versus imipenem-cilastatin for the treatment of complicated urinary tract infections caused by Gram-negative uropathogens: A phase 2, randomised, double-blind, non-inferiority trial. Lancet Infect. Dis..

[250] Wunderink R.G., Matsunaga Y., Ariyasu M., Clevenbergh P., Echols R., Kaye K.S., Kollef M., Menon A., Pogue J.M., Shorr A.F. (2021). Cefiderocol versus high-dose, extended-infusion meropenem for the treatment of Gram-negative nosocomial pneumonia (APEKS-NP): A randomised, double-blind, phase 3, non-inferiority trial. Lancet Infect. Dis..

[251] Burnard D., Robertson G., Henderson A., Falconer C., Bauer M.J., Cottrell K., Gassiep I., Norton R., Paterson D.L., Harris P.N.A. (2021). Burkholderia pseudomallei Clinical Isolates Are Highly Susceptible In Vitro to Cefiderocol, a Siderophore Cephalosporin. Antimicrob. Agents Chemother..

[252] Pybus C.A., Felder-Scott C., Obuekwe V., Greenberg D.E. (2021). Cefiderocol Retains Antibiofilm Activity in Multidrug-Resistant Gram-Negative Pathogens. Antimicrob. Agents Chemother..

[253] Findlay J., Bianco G., Boattini M., Nordmann P. (2024). In vivo development of cefiderocol resistance in carbapenem-resistant Acinetobacter baumannii associated with the downregulation of a TonB-dependent siderophore receptor, PiuA. J. Antimicrob. Chemother..

[254] Egge S.L., Rizvi S.A., Simar S.R., Alcalde M., Martinez J.R.W., Hanson B.M., Dinh A.Q., Baptista R.P., Tran T.T., Shelburne S.A. (2024). Cefiderocol heteroresistance associated with mutations in TonB-dependent receptor genes in Pseudomonas aeruginosa of clinical origin. Antimicrob. Agents Chemother..

[255] Lan P., Lu Y., Liao W., Yu Y., Fu Y., Zhou J. (2025). Cefiderocol-resistant hypervirulent Klebsiella pneumoniae with CirA deficiency and co-production of KPC-2 and SHV-12. Clin. Microbiol. Infect..

[256] Kang D., Baptista R.P., Drusin S.I., Moreno D.M., Arias C.A., Miller W.R. (2026). Loss of the ferripyochelin receptor FptA drives reduced cefiderocol susceptibility and impairs fitness in Pseudomonas aeruginosa PA14. Antimicrob. Agents Chemother..

[257] Ji X.W., Xue F., Kang Z.S., Zhong W., Kuan I.H., Yang X.P., Zhu X., Li Y., Lv Y. (2020). Model-Informed Drug Development, Pharmacokinetic/Pharmacodynamic Cutoff Value Determination, and Antibacterial Efficacy of Benapenem against Enterobacteriaceae. Antimicrob. Agents Chemother..

[258] Wagenlehner F.M., Gasink L.B., McGovern P.C., Moeck G., McLeroth P., Dorr M., Dane A., Henkel T. (2024). Cefepime-Taniborbactam in Complicated Urinary Tract Infection. N. Engl. J. Med..

[259] Piccirilli A., Segatore B., Brisdelli F., Amicosante G., Perilli M. (2021). Potent inhibitory activity of taniborbactam towards NDM-1 and NDM-1(Q119X) mutants, and in vitro activity of cefepime/taniborbactam against MBLs producing Enterobacterales. Int. J. Antimicrob. Agents.

[260] Mushtaq S., Vickers A., Doumith M., Ellington M.J., Woodford N., Livermore D.M. (2021). Activity of β-lactam/taniborbactam (VNRX-5133) combinations against carbapenem-resistant Gram-negative bacteria. J. Antimicrob. Chemother..

[261] Kloezen W., Melchers R.J., Georgiou P.C., Mouton J.W., Meletiadis J. (2021). Activity of Cefepime in Combination with the Novel β-Lactamase Inhibitor Taniborbactam (VNRX-5133) against Extended-Spectrum-β-Lactamase-Producing Isolates in In Vitro Checkerboard Assays. Antimicrob. Agents Chemother..

[262] Lasko M.J., Nicolau D.P., Asempa T.E. (2022). Clinical exposure-response relationship of cefepime/taniborbactam against Gram-negative organisms in the murine complicated urinary tract infection model. J. Antimicrob. Chemother..

[263] Mojica M.F., Zeiser E.T., Becka S.A., LiPuma J.J., Six D.A., Moeck G., Papp-Wallace K.M. (2023). Examining the activity of cefepime-taniborbactam against Burkholderia cepacia complex and Burkholderia gladioli isolated from cystic fibrosis patients in the United States. Antimicrob. Agents Chemother..

[264] Moeck G., Gasink L.B., Mendes R.E., Woosley L.N., Dorr M., Chen H., Wagenlehner F.M., Henkel T., McGovern P.C. (2024). Patient outcomes by baseline pathogen resistance phenotype and genotype in CERTAIN-1, a Phase 3 study of cefepime-taniborbactam versus meropenem in adults with complicated urinary tract infection. Antimicrob. Agents Chemother..

[265] Mack A.R., Kumar V., Bethel C.R., Taracila M.A., Miller B.A., Uehara T., Six D.A., Papp-Wallace K.M., van den Akker F., Bonomo R.A. (2025). Structure and mechanism of taniborbactam inhibition of the cefepime-hydrolyzing, partial R2-loop deletion Pseudomonas-derived cephalosporinase variant PDC-88. Antimicrob. Agents Chemother..

[266] Mojica M.F., Becka S.A., Edwards M., Myers C., Uehara K., Uehara T., Hoshino T., Zeiser E.T., Chatwin C.L., Six D.A. (2025). Burkholderia pseudomallei PenI β-lactamase and variants are potently inhibited by taniborbactam. Antimicrob. Agents Chemother..

[267] Liu B., Trout R.E.L., Chu G.H., McGarry D., Jackson R.W., Hamrick J.C., Daigle D.M., Cusick S.M., Pozzi C., De Luca F. (2020). Discovery of Taniborbactam (VNRX-5133): A Broad-Spectrum Serine- and Metallo-β-lactamase Inhibitor for Carbapenem-Resistant Bacterial Infections. J. Med. Chem..

[268] Hamrick J.C., Docquier J.D., Uehara T., Myers C.L., Six D.A., Chatwin C.L., John K.J., Vernacchio S.F., Cusick S.M., Trout R.E.L. (2020). VNRX-5133 (Taniborbactam), a Broad-Spectrum Inhibitor of Serine- and Metallo-β-Lactamases, Restores Activity of Cefepime in Enterobacterales and Pseudomonas aeruginosa. Antimicrob. Agents Chemother..

[269] Drusin S.I., Le Terrier C., Poirel L., Bonomo R.A., Vila A.J., Moreno D.M. (2024). Structural basis of metallo-β-lactamase resistance to taniborbactam. Antimicrob. Agents Chemother..

[270] Bonnefoy A., Dupuis-Hamelin C., Steier V., Delachaume C., Seys C., Stachyra T., Fairley M., Guitton M., Lampilas M. (2004). In vitro activity of AVE1330A, an innovative broad-spectrum non-beta-lactam beta-lactamase inhibitor. J. Antimicrob. Chemother..

[271] Endimiani A., Choudhary Y., Bonomo R.A. (2009). In vitro activity of NXL104 in combination with beta-lactams against *Klebsiella pneumoniae* isolates producing KPC carbapenemases. Antimicrob. Agents Chemother..

[272] Crandon J.L., Schuck V.J., Banevicius M.A., Beaudoin M.E., Nichols W.W., Tanudra M.A., Nicolau D.P. (2012). Comparative in vitro and in vivo efficacies of human simulated doses of ceftazidime and ceftazidime-avibactam against *Pseudomonas aeruginosa*. Antimicrob. Agents Chemother..

[273] Lucasti C., Popescu I., Ramesh M.K., Lipka J., Sable C. (2013). Comparative study of the efficacy and safety of ceftazidime/avibactam plus metronidazole versus meropenem in the treatment of complicated intra-abdominal infections in hospitalized adults: Results of a randomized, double-blind, Phase II trial. J. Antimicrob. Chemother..

[274] Castanheira M., Farrell S.E., Krause K.M., Jones R.N., Sader H.S. (2014). Contemporary diversity of β-lactamases among Enterobacteriaceae in the nine U.S. census regions and ceftazidime-avibactam activity tested against isolates producing the most prevalent β-lactamase groups. Antimicrob. Agents Chemother..

[275] MacVane S.H., Crandon J.L., Nichols W.W., Nicolau D.P. (2014). In vivo efficacy of humanized exposures of Ceftazidime-Avibactam in comparison with Ceftazidime against contemporary Enterobacteriaceae isolates. Antimicrob. Agents Chemother..

[276] Carmeli Y., Armstrong J., Laud P.J., Newell P., Stone G., Wardman A., Gasink L.B. (2016). Ceftazidime-avibactam or best available therapy in patients with ceftazidime-resistant Enterobacteriaceae and Pseudomonas aeruginosa complicated urinary tract infections or complicated intra-abdominal infections (REPRISE): A randomised, pathogen-directed, phase 3 study. Lancet Infect. Dis..

[277] Wagenlehner F.M., Sobel J.D., Newell P., Armstrong J., Huang X., Stone G.G., Yates K., Gasink L.B. (2016). Ceftazidime-avibactam Versus Doripenem for the Treatment of Complicated Urinary Tract Infections, Including Acute Pyelonephritis: RECAPTURE, a Phase 3 Randomized Trial Program. Clin. Infect. Dis..

[278] Tamma P.D., Fan Y., Bergman Y., Sick-Samuels A.C., Hsu A.J., Timp W., Simner P.J., Prokesch B.C., Greenberg D.E. (2018). Successful Treatment of Persistent Burkholderia cepacia Complex Bacteremia with Ceftazidime-Avibactam. Antimicrob. Agents Chemother..

[279] Lai Y.C., Lin L.W., Cheng Y.C., Lee Y.L. (2024). Conversion of bla(KPC-2) to bla(KPC-33) leads to worldwide emergence of ceftazidime-avibactam resistance in Klebsiella pneumoniae. Int. J. Antimicrob. Agents.

[280] Karaba S.M., Hareza D.A., Munita J.M., Martinez J.R.W., Bergman Y., Hawes A.M., Bulman Z.P., Cosgrove S.E., Simner P.J., Tamma P.D. (2026). Comparative outcomes of ceftazidime-avibactam versus meropenem-vaborbactam for KPC-producing Enterobacterales infections. Antimicrob. Agents Chemother..

[281] Kunz Coyne A.J., Judd C., Lucas K., Al Musawa M., Van Helden S., Holger D., Althubyani A., Yost C., VanDorf S., Mynatt R. (2026). Ceftazidime-avibactam for multidrug-resistant gram-negative infections: Outcomes and timing of initiation across 22 U.S. medical centers. Antimicrob. Agents Chemother..

[282] Sader H.S., Rhomberg P.R., Farrell D.J., Jones R.N. (2011). Antimicrobial activity of CXA-101, a novel cephalosporin tested in combination with tazobactam against Enterobacteriaceae, Pseudomonas aeruginosa, and Bacteroides fragilis strains having various resistance phenotypes. Antimicrob. Agents Chemother..

[283] Snydman D.R., McDermott L.A., Jacobus N.V. (2014). Activity of ceftolozane-tazobactam against a broad spectrum of recent clinical anaerobic isolates. Antimicrob. Agents Chemother..

[284] Cabot G., Bruchmann S., Mulet X., Zamorano L., Moyà B., Juan C., Haussler S., Oliver A. (2014). Pseudomonas aeruginosa ceftolozane-tazobactam resistance development requires multiple mutations leading to overexpression and structural modification of AmpC. Antimicrob. Agents Chemother..

[285] Wagenlehner F.M., Umeh O., Steenbergen J., Yuan G., Darouiche R.O. (2015). Ceftolozane-tazobactam compared with levofloxacin in the treatment of complicated urinary-tract infections, including pyelonephritis: A randomised, double-blind, phase 3 trial (ASPECT-cUTI). Lancet.

[286] Miller B., Popejoy M.W., Hershberger E., Steenbergen J.N., Alverdy J. (2016). Characteristics and Outcomes of Complicated Intra-abdominal Infections Involving Pseudomonas aeruginosa from a Randomized, Double-Blind, Phase 3 Ceftolozane-Tazobactam Study. Antimicrob. Agents Chemother..

[287] Mazer D.M., Young C., Kalikin L.M., Spilker T., LiPuma J.J. (2017). In Vitro Activity of Ceftolozane-Tazobactam and Other Antimicrobial Agents against Burkholderia cepacia Complex and Burkholderia gladioli. Antimicrob. Agents Chemother..

[288] Fraile-Ribot P.A., Mulet X., Cabot G., Del Barrio-Tofiño E., Juan C., Pérez J.L., Oliver A. (2017). In Vivo Emergence of Resistance to Novel Cephalosporin-β-Lactamase Inhibitor Combinations through the Duplication of Amino Acid D149 from OXA-2 β-Lactamase (OXA-539) in Sequence Type 235 Pseudomonas aeruginosa. Antimicrob. Agents Chemother..

[289] Fraile-Ribot P.A., Cabot G., Mulet X., Periañez L., Martín-Pena M.L., Juan C., Pérez J.L., Oliver A. (2018). Mechanisms leading to in vivo ceftolozane/tazobactam resistance development during the treatment of infections caused by MDR Pseudomonas aeruginosa. J. Antimicrob. Chemother..

[290] Chan E., Martelli P., Hui S.W., Teng J.L.L., Lau S.K.P., Woo P.C.Y. (2018). In Vitro Susceptibility of Ceftolozane-Tazobactam against Burkholderia pseudomallei. Antimicrob. Agents Chemother..

[291] Chan E., Wu A.K.L., Tse C.W.S., Lau S.K.P., Woo P.C.Y. (2019). In vitro susceptibility of Salmonella enterica serovar Typhi to ceftolozane/tazobactam. J. Antimicrob. Chemother..

[292] Sader H.S., Carvalhaes C.G., Duncan L.R., Shortridge D. (2020). Antimicrobial Activity of Ceftolozane-Tazobactam and Comparators against Clinical Isolates of Haemophilus influenzae from the United States and Europe. Antimicrob. Agents Chemother..

[293] Arca-Suárez J., Lasarte-Monterrubio C., Rodiño-Janeiro B.K., Cabot G., Vázquez-Ucha J.C., Rodríguez-Iglesias M., Galán-Sánchez F., Beceiro A., González-Bello C., Oliver A. (2021). Molecular mechanisms driving the in vivo development of OXA-10-mediated resistance to ceftolozane/tazobactam and ceftazidime/avibactam during treatment of XDR Pseudomonas aeruginosa infections. J. Antimicrob. Chemother..

[294] Fournier D., Carrière R., Bour M., Grisot E., Triponney P., Muller C., Lemoine J., Jeannot K., Plésiat P. (2021). Mechanisms of Resistance to Ceftolozane/Tazobactam in Pseudomonas aeruginosa: Results of the GERPA Multicenter Study. Antimicrob. Agents Chemother..

[295] Gomis-Font M.A., Pitart C., Del Barrio-Tofiño E., Zboromyrska Y., Cortes-Lara S., Mulet X., Marco F., Vila J., López-Causapé C., Oliver A. (2021). Emergence of Resistance to Novel Cephalosporin-β-Lactamase Inhibitor Combinations through the Modification of the Pseudomonas aeruginosa MexCD-OprJ Efflux Pump. Antimicrob. Agents Chemother..

[296] Teng J.L.L., Chan E., Dai A.C.H., Ng G., Li T.T., Lai C., Wu A.K.L., Lau S.K.P., Woo P.C.Y. (2022). In Vitro Susceptibility of Typhoidal, Nontyphoidal, and Extended-Spectrum-β-Lactamase-Producing Salmonella to Ceftolozane/Tazobactam. Antimicrob. Agents Chemother..

[297] Kostoulias X., Fu Y., Morris F.C., Yu C., Qu Y., Chang C.C., Blakeway L., Landersdorfer C.B., Abbott I.J., Wang L. (2025). Ceftolozane/tazobactam disrupts Pseudomonas aeruginosa biofilms under static and dynamic conditions. J. Antimicrob. Chemother..

[298] Shields R.K., Abbo L.M., Ackley R., Aitken S.L., Albrecht B., Babiker A., Burgoon R., Cifuentes R., Claeys K.C., Curry B.N. (2025). Effectiveness of ceftazidime-avibactam versus ceftolozane-tazobactam for multidrug-resistant Pseudomonas aeruginosa infections in the USA (CACTUS): A multicentre, retrospective, observational study. Lancet Infect. Dis..

[299] Durand-Réville T.F., Guler S., Comita-Prevoir J., Chen B., Bifulco N., Huynh H., Lahiri S., Shapiro A.B., McLeod S.M., Carter N.M. (2017). ETX2514 is a broad-spectrum β-lactamase inhibitor for the treatment of drug-resistant Gram-negative bacteria including Acinetobacter baumannii. Nat. Microbiol..

[300] Barnes M.D., Kumar V., Bethel C.R., Moussa S.H., O’Donnell J., Rutter J.D., Good C.E., Hujer K.M., Hujer A.M., Marshall S.H. (2019). Targeting Multidrug-Resistant Acinetobacter spp.: Sulbactam and the Diazabicyclooctenone β-Lactamase Inhibitor ETX2514 as a Novel Therapeutic Agent. mBio.

[301] McLeod S.M., Moussa S.H., Hackel M.A., Miller A.A. (2020). In Vitro Activity of Sulbactam-Durlobactam against Acinetobacter baumannii-calcoaceticus Complex Isolates Collected Globally in 2016 and 2017. Antimicrob. Agents Chemother..

[302] Papp-Wallace K.M., Shapiro A.B., Becka S.A., Zeiser E.T., LiPuma J.J., Lane D.J., Panchal R.G., Mueller J.P., O’Donnell J.P., Miller A.A. (2021). In Vitro Antibacterial Activity and In Vivo Efficacy of Sulbactam-Durlobactam against Pathogenic Burkholderia Species. Antimicrob. Agents Chemother..

[303] Kaye K.S., Shorr A.F., Wunderink R.G., Du B., Poirier G.E., Rana K., Miller A., Lewis D., O’Donnell J., Chen L. (2023). Efficacy and safety of sulbactam-durlobactam versus colistin for the treatment of patients with serious infections caused by Acinetobacter baumannii-calcoaceticus complex: A multicentre, randomised, active-controlled, phase 3, non-inferiority clinical trial (ATTACK). Lancet Infect. Dis..

[304] O’Donnell J., Tanudra A., Chen A., Newman J., McLeod S.M., Tommasi R. (2024). In vivo dose response and efficacy of the β-lactamase inhibitor, durlobactam, in combination with sulbactam against the Acinetobacter baumannii-calcoaceticus complex. Antimicrob. Agents Chemother..

[305] Andries K., Verhasselt P., Guillemont J., Göhlmann H.W., Neefs J.M., Winkler H., Van Gestel J., Timmerman P., Zhu M., Lee E. (2005). A diarylquinoline drug active on the ATP synthase of Mycobacterium tuberculosis. Science.

[306] Ji B., Chauffour A., Andries K., Jarlier V. (2006). Bactericidal activities of R207910 and other newer antimicrobial agents against *Mycobacterium leprae* in mice. Antimicrob. Agents Chemother..

[307] Ji B., Lefrançois S., Robert J., Chauffour A., Truffot C., Jarlier V. (2006). In vitro and in vivo activities of rifampin, streptomycin, amikacin, moxifloxacin, R207910, linezolid, and PA-824 against Mycobacterium ulcerans. Antimicrob. Agents Chemother..

[308] Koul A., Dendouga N., Vergauwen K., Molenberghs B., Vranckx L., Willebrords R., Ristic Z., Lill H., Dorange I., Guillemont J. (2007). Diarylquinolines target subunit c of mycobacterial ATP synthase. Nat. Chem. Biol..

[309] Rustomjee R., Diacon A.H., Allen J., Venter A., Reddy C., Patientia R.F., Mthiyane T.C., De Marez T., van Heeswijk R., Kerstens R. (2008). Early bactericidal activity and pharmacokinetics of the diarylquinoline TMC207 in treatment of pulmonary tuberculosis. Antimicrob. Agents Chemother..

[310] Diacon A.H., Pym A., Grobusch M., Patientia R., Rustomjee R., Page-Shipp L., Pistorius C., Krause R., Bogoshi M., Churchyard G. (2009). The diarylquinoline TMC207 for multidrug-resistant tuberculosis. N. Engl. J. Med..

[311] Hards K., Robson J.R., Berney M., Shaw L., Bald D., Koul A., Andries K., Cook G.M. (2015). Bactericidal mode of action of bedaquiline. J. Antimicrob. Chemother..

[312] Pym A.S., Diacon A.H., Tang S.J., Conradie F., Danilovits M., Chuchottaworn C., Vasilyeva I., Andries K., Bakare N., De Marez T. (2016). Bedaquiline in the treatment of multidrug- and extensively drug-resistant tuberculosis. Eur. Respir. J..

[313] Kundu S., Biukovic G., Grüber G., Dick T. (2016). Bedaquiline Targets the ε Subunit of Mycobacterial F-ATP Synthase. Antimicrob. Agents Chemother..

[314] Brown-Elliott B.A., Philley J.V., Griffith D.E., Thakkar F., Wallace R.J. (2017). In Vitro Susceptibility Testing of Bedaquiline against Mycobacterium avium Complex. Antimicrob. Agents Chemother..

[315] Borisov S.E., Dheda K., Enwerem M., Romero Leyet R., D’Ambrosio L., Centis R., Sotgiu G., Tiberi S., Alffenaar J.W., Maryandyshev A. (2017). Effectiveness and safety of bedaquiline-containing regimens in the treatment of MDR- and XDR-TB: A multicentre study. Eur. Respir. J..

[316] Li S.Y., Tasneen R., Tyagi S., Soni H., Converse P.J., Mdluli K., Nuermberger E.L. (2017). Bactericidal and Sterilizing Activity of a Novel Regimen with Bedaquiline, Pretomanid, Moxifloxacin, and Pyrazinamide in a Murine Model of Tuberculosis. Antimicrob. Agents Chemother..

[317] Dupont C., Viljoen A., Thomas S., Roquet-Banères F., Herrmann J.L., Pethe K., Kremer L. (2017). Bedaquiline Inhibits the ATP Synthase in Mycobacterium abscessus and Is Effective in Infected Zebrafish. Antimicrob. Agents Chemother..

[318] Pamreddy A., Baijnath S., Naicker T., Ntshangase S., Mdanda S., Lubanyana H., Kruger H.G., Govender T. (2018). Bedaquiline has potential for targeting tuberculosis reservoirs in the central nervous system. RSC Adv..

[319] Chawla K., Martinez E., Kumar A., Shenoy V.P., Sintchenko V. (2018). Whole-genome sequencing reveals genetic signature of bedaquiline resistance in a clinical isolate of Mycobacterium tuberculosis. J. Glob. Antimicrob. Resist..

[320] Ismail N., Ismail N.A., Omar S.V., Peters R.P.H. (2019). In Vitro Study of Stepwise Acquisition of rv0678 and atpE Mutations Conferring Bedaquiline Resistance. Antimicrob. Agents Chemother..

[321] Sarathy J.P., Ragunathan P., Shin J., Cooper C.B., Upton A.M., Grüber G., Dick T. (2019). TBAJ-876 Retains Bedaquiline’s Activity against Subunits c and ε of Mycobacterium tuberculosis F-ATP Synthase. Antimicrob. Agents Chemother..

[322] Burki T. (2019). BPaL approved for multidrug-resistant tuberculosis. Lancet Infect. Dis..

[323] Erber J., Weidlich S., Tschaikowsky T., Rothe K., Schmid R.M., Schneider J., Spinner C.D. (2020). Successful bedaquiline-containing antimycobacterial treatment in post-traumatic skin and soft-tissue infection by Mycobacterium fortuitum complex: A case report. BMC Infect. Dis..

[324] Guo H., Courbon G.M., Bueler S.A., Mai J., Liu J., Rubinstein J.L. (2021). Structure of mycobacterial ATP synthase bound to the tuberculosis drug bedaquiline. Nature.

[325] Xu J., Converse P.J., Upton A.M., Mdluli K., Fotouhi N., Nuermberger E.L. (2021). Comparative Efficacy of the Novel Diarylquinoline TBAJ-587 and Bedaquiline against a Resistant Rv0678 Mutant in a Mouse Model of Tuberculosis. Antimicrob. Agents Chemother..

[326] Conradie F., Bagdasaryan T.R., Borisov S., Howell P., Mikiashvili L., Ngubane N., Samoilova A., Skornykova S., Tudor E., Variava E. (2022). Bedaquiline-Pretomanid-Linezolid Regimens for Drug-Resistant Tuberculosis. N. Engl. J. Med..

[327] Zhang Y., Lai Y., Zhou S., Ran T., Zhang Y., Zhao Z., Feng Z., Yu L., Xu J., Shi K. (2024). Inhibition of M. tuberculosis and human ATP synthase by BDQ and TBAJ-587. Nature.

[328] Barreto J., Sammarco Rosa P., Adams L., Aguilar Z., Bakare N., Chaplan S.R., Akli R.D., Ernault E., Kulke S., Lounis N. (2024). Bedaquiline Monotherapy for Multibacillary Leprosy. N. Engl. J. Med..

[329] Goodall R.L., Nunn A.J., Meredith S.K., Bayissa A., Bhatnagar A.K., Chiang C.Y., Conradie F., Gopalan N., Gurumurthy M., Kirenga B. (2024). Long-term efficacy and safety of two short standardised regimens for the treatment of rifampicin-resistant tuberculosis (STREAM stage 2): Extended follow-up of an open-label, multicentre, randomised, non-inferiority trial. Lancet Respir. Med..

[330] Guglielmetti L., Khan U., Velásquez G.E., Gouillou M., Ali M.H., Amjad S., Kamal F., Abubakirov A., Ardizzoni E., Baudin E. (2025). Bedaquiline, delamanid, linezolid, and clofazimine for rifampicin-resistant and fluoroquinolone-resistant tuberculosis (endTB-Q): An open-label, multicentre, stratified, non-inferiority, randomised, controlled, phase 3 trial. Lancet Respir. Med..

[331] Nedelman J., Conradie A., Lombardi A., Hickman D., Abd-Elaziz K., Dogterom P., Bruinenberg P., Sansuán Antonio J.C., Sakiman E., Diamant Z. (2026). Pharmacokinetics and safety of TBAJ-587, a novel antimycobacterial diarylquinoline, in healthy participants. Antimicrob. Agents Chemother..

[332] Darpo B., Nedelman J., Bruning-Barry R., Hickman D., Kleiman R., Lombardi A., Xue H. (2026). Cardiodynamic evaluation of sorfequiline (TBAJ-876): Results from a first-in-human study. Antimicrob. Agents Chemother..

[333] Yao R., Wang B., Fu L., Li L., You K., Li Y.G., Lu Y. (2022). Sudapyridine (WX-081), a Novel Compound against Mycobacterium tuberculosis. Microbiol. Spectr..

[334] Huang Z., Luo W., Xu D., Guo F., Yang M., Zhu Y., Shen L., Chen S., Tang D., Li L. (2022). Discovery and preclinical profile of sudapyridine (WX-081), a novel anti-tuberculosis agent. Bioorg Med. Chem. Lett..

[335] Yu W., Ju Y., Han X., Tian X., Ding J., Wang S., Hameed H.M.A., Gao Y., Li L., Li Y. (2024). Bactericidal and sterilizing activity of sudapyridine-clofazimine-TB47 combined with linezolid or pyrazinamide in a murine model of tuberculosis. Antimicrob. Agents Chemother..

[336] Zhu R., Shang Y., Chen S., Xiao H., Ren R., Wang F., Xue Y., Li L., Li Y., Chu N. (2022). In Vitro Activity of the Sudapyridine (WX-081) against Non-Tuberculous Mycobacteria Isolated in Beijing, China. Microbiol. Spectr..

[337] Nie W., Gao S., Su L., Liu L., Geng R., You Y., Chu N. (2023). Antibacterial activity of the novel compound Sudapyridine (WX-081) against Mycobacterium abscessus. Front. Cell Infect. Microbiol..

[338] Zheng L., Wang H., Qi X., Zhang W., Wang B., Fu L., Chen X., Chen X., Lu Y. (2024). Sudapyridine (WX-081) antibacterial activity against Mycobacterium avium, Mycobacterium abscessus, and Mycobacterium chelonae in vitro and in vivo. Msphere.

[339] Wang H., Zheng L., Fu L., Zhang W., Dong S., Chen X., Lu Y. (2025). In vitro and in vivo antibacterial activity of sudapyridine (WX-081) combined with other drugs against Mycobacterium abscessus. Tuberculosis.

[340] Li X., Luo X., Wang B., Fu L., Chen X., Lu Y. (2025). Sudapyridine (WX-081) inhibits Mycobacterium tuberculosis by targeting ATP synthase and upregulating host innate immunity. Msphere.

[341] Kong W., Liang H., Zhang Y., Li L., Li Y., Yan X., Liu D. (2025). Population pharmacokinetic and exposure-response study of a novel anti-tuberculosis drug to inform its dosage design in phase III clinical trial. Eur. J. Pharm. Sci..

[342] Pethe K., Bifani P., Jang J., Kang S., Park S., Ahn S., Jiricek J., Jung J., Jeon H.K., Cechetto J. (2013). Discovery of Q203, a potent clinical candidate for the treatment of tuberculosis. Nat. Med..

[343] Scherr N., Bieri R., Thomas S.S., Chauffour A., Kalia N.P., Schneide P., Ruf M.T., Lamelas A., Manimekalai M.S.S., Grüber G. (2018). Targeting the Mycobacterium ulcerans cytochrome bc(1):aa(3) for the treatment of Buruli ulcer. Nat. Commun..

[344] Lahiri R., Adams L.B., Thomas S.S., Pethe K. (2022). Sensitivity of Mycobacterium leprae to Telacebec. Emerg. Infect. Dis..

[345] Janssen S., Upton C., de Jager V.R., van Niekerk C., Dawson R., Hutchings J., Kim J., Choi J., Nam K., Sun E. (2025). Telacebec, a Potent Agent in the Fight against Tuberculosis: Findings from a Randomized, Phase 2 Clinical Trial and Beyond. Am. J. Respir. Crit. Care Med..

[346] O’Brien D., Muhi S., Ellis K., Sarah B., Savvides N., Henderson J., Betteridge M., Hickman D., O’Keeffe J.C., Maggs C. (2026). 547. An Open-label Multicenter Phase 2 Trial of Telacebec for Adults with Buruli ulcer: The TREAT-BU Trial. Open Forum Infect. Dis..

[347] Makarov V., Manina G., Mikusova K., Möllmann U., Ryabova O., Saint-Joanis B., Dhar N., Pasca M.R., Buroni S., Lucarelli A.P. (2009). Benzothiazinones kill Mycobacterium tuberculosis by blocking arabinan synthesis. Science.

[348] Grover S., Alderwick L.J., Mishra A.K., Krumbach K., Marienhagen J., Eggeling L., Bhatt A., Besra G.S. (2014). Benzothiazinones mediate killing of Corynebacterineae by blocking decaprenyl phosphate recycling involved in cell wall biosynthesis. J. Biol. Chem..

[349] González-Martínez N.A., Lozano-Garza H.G., Castro-Garza J., De Osio-Cortez A., Vargas-Villarreal J., Cavazos-Rocha N., Ocampo-Candiani J., Makarov V., Cole S.T., Vera-Cabrera L. (2015). In Vivo Activity of the Benzothiazinones PBTZ169 and BTZ043 against Nocardia brasiliensis. PLoS Negl. Trop. Dis..

[350] Eckhardt E., Li Y., Mamerow S., Schinköthe J., Sehl-Ewert J., Dreisbach J., Corleis B., Dorhoi A., Teifke J., Menge C. (2023). Pharmacokinetics and Efficacy of the Benzothiazinone BTZ-043 against Tuberculous Mycobacteria inside Granulomas in the Guinea Pig Model. Antimicrob. Agents Chemother..

[351] Ramey M.E., Kaya F., Bauman A.A., Massoudi L.M., Sarathy J.P., Zimmerman M.D., Scott D.W.L., Job A.M., Miller-Dawson J.A., Podell B.K. (2023). Drug distribution and efficacy of the DprE1 inhibitor BTZ-043 in the C3HeB/FeJ mouse tuberculosis model. Antimicrob. Agents Chemother..

[352] Heinrich N., de Jager V., Dreisbach J., Gross-Demel P., Schultz S., Gerbach S., Kloss F., Dawson R., Narunsky K., Matt L. (2025). Safety, bactericidal activity, and pharmacokinetics of the antituberculosis drug candidate BTZ-043 in South Africa (PanACEA-BTZ-043-02): An open-label, dose-expansion, randomised, controlled, phase 1b/2a trial. Lancet Microbe.

[353] Lutchmun W., Heinrich N., Svensson E.M., Kloss F., Konsten S., Dreisbach J., Hoelscher M. (2025). First-in-human study of the benzothiazinone and DprE1 inhibitor BTZ-043, a novel drug candidate for the treatment of Tuberculosis. JAC Antimicrob. Resist..

[354] Römpp A., Treu A., Kokesch-Himmelreich J., Marwitz F., Dreisbach J., Aboutara N., Hillemann D., Garrelts M., Converse P.J., Tyagi S. (2025). The clinical-stage drug BTZ-043 accumulates in murine tuberculosis lesions and efficiently acts against Mycobacterium tuberculosis. Nat. Commun..

[355] Makarov V., Lechartier B., Zhang M., Neres J., van der Sar A.M., Raadsen S.A., Hartkoorn R.C., Ryabova O.B., Vocat A., Decosterd L.A. (2014). Towards a new combination therapy for tuberculosis with next generation benzothiazinones. EMBO Mol. Med..

[356] Lupien A., Vocat A., Foo C.S., Blattes E., Gillon J.Y., Makarov V., Cole S.T. (2018). Optimized Background Regimen for Treatment of Active Tuberculosis with the Next-Generation Benzothiazinone Macozinone (PBTZ169). Antimicrob. Agents Chemother..

[357] Mariandyshev A.O., Khokhlov A.L., Smerdin S.V., Shcherbakova V.S., Igumnova O.V., Ozerova I.V., Bolgarina A.A., Nikitina N.A. (2020). The main results of clinical trials of the efficacy, safety and pharmacokinetics of the perspective anti-tuberculosis drug makozinone (PBTZ169). Ter. Arkhiv..

[358] Hariguchi N., Chen X., Hayashi Y., Kawano Y., Fujiwara M., Matsuba M., Shimizu H., Ohba Y., Nakamura I., Kitamoto R. (2020). OPC-167832, a Novel Carbostyril Derivative with Potent Antituberculosis Activity as a DprE1 Inhibitor. Antimicrob. Agents Chemother..

[359] Sarathy J.P., Zimmerman M.D., Gengenbacher M., Dartois V., Dick T. (2022). Mycobacterium tuberculosis DprE1 Inhibitor OPC-167832 Is Active against Mycobacterium abscessus In Vitro. Antimicrob. Agents Chemother..

[360] Dawson R., Diacon A.H., De Jager V., Narunsky K., Moodley V.M., Stinson K.W., Liu Y., Zheng B., Hafkin J. (2025). Safety, pharmacokinetics, and early bactericidal activity of quabodepistat in combination with delamanid, bedaquiline, or both in adults with pulmonary tuberculosis: A randomised, active-controlled, open-label trial. Lancet Infect. Dis..

[361] Lee R.E., Protopopova M., Crooks E., Slayden R.A., Terrot M., Barry C.E. (2003). Combinatorial lead optimization of [1,2]-diamines based on ethambutol as potential antituberculosis preclinical candidates. J. Comb. Chem..

[362] Protopopova M., Hanrahan C., Nikonenko B., Samala R., Chen P., Gearhart J., Einck L., Nacy C.A. (2005). Identification of a new antitubercular drug candidate, SQ109, from a combinatorial library of 1,2-ethylenediamines. J. Antimicrob. Chemother..

[363] Tahlan K., Wilson R., Kastrinsky D.B., Arora K., Nair V., Fischer E., Barnes S.W., Walker J.R., Alland D., Barry C.E. (2012). SQ109 targets MmpL3, a membrane transporter of trehalose monomycolate involved in mycolic acid donation to the cell wall core of Mycobacterium tuberculosis. Antimicrob. Agents Chemother..

[364] Makobongo M.O., Einck L., Peek R.M., Merrell D.S. (2013). In vitro characterization of the anti-bacterial activity of SQ109 against Helicobacter pylori. PLoS ONE.

[365] Heinrich N., Dawson R., du Bois J., Narunsky K., Horwith G., Phipps A.J., Nacy C.A., Aarnoutse R.E., Boeree M.J., Gillespie S.H. (2015). Early phase evaluation of SQ109 alone and in combination with rifampicin in pulmonary TB patients. J. Antimicrob. Chemother..

[366] Egbelowo O., Sarathy J.P., Gausi K., Zimmerman M.D., Wang H., Wijnant G.J., Kaya F., Gengenbacher M., Van N., Degefu Y. (2021). Pharmacokinetics and Target Attainment of SQ109 in Plasma and Human-Like Tuberculosis Lesions in Rabbits. Antimicrob. Agents Chemother..

[367] Johnson A.P., Warner M., Livermore D.M. (2002). In vitro activity of a novel oxazolidinone, AZD2563, against randomly selected and multiresistant Gram-positive cocci. J. Antimicrob. Chemother..

[368] Ednie L.M., Jacobs M.R., Appelbaum P.C. (2002). Anti-anaerobic activity of AZD2563, a new oxazolidinone, compared with eight other agents. J. Antimicrob. Chemother..

[369] Werngren J., Wijkander M., Perskvist N., Balasubramanian V., Sambandamurthy V.K., Rodrigues C., Hoffner S. (2014). In vitro activity of AZD5847 against geographically diverse clinical isolates of Mycobacterium tuberculosis. Antimicrob. Agents Chemother..

[370] Alsultan A., Furin J.J., Du Bois J., van Brakel E., Chheng P., Venter A., Thiel B., Debanne S.A., Boom W.H., Diacon A.H. (2017). Population Pharmacokinetics of AZD-5847 in Adults with Pulmonary Tuberculosis. Antimicrob. Agents Chemother..

[371] Furin J.J., Du Bois J., van Brakel E., Chheng P., Venter A., Peloquin C.A., Alsultan A., Thiel B.A., Debanne S.M., Boom W.H. (2016). Early Bactericidal Activity of AZD5847 in Patients with Pulmonary Tuberculosis. Antimicrob. Agents Chemother..

[372] Li C.R., Zhai Q.Q., Wang X.K., Hu X.X., Li G.Q., Zhang W.X., Pang J., Lu X., Yuan H., Gordeev M.F. (2014). In vivo antibacterial activity of MRX-I, a new oxazolidinone. Antimicrob. Agents Chemother..

[373] Huang Y., Xu Y., Liu S., Wang H., Xu X., Guo Q., Wu B., Gordeev M.F., Wang W., Yuan Z. (2014). Selection and characterisation of *Staphylococcus aureus* mutants with reduced susceptibility to the investigational oxazolidinone MRX-I. Int. J. Antimicrob. Agents.

[374] Shoen C., DeStefano M., Hafkin B., Cynamon M. (2018). In Vitro and In Vivo Activities of Contezolid (MRX-I) against Mycobacterium tuberculosis. Antimicrob. Agents Chemother..

[375] Carvalhaes C.G., Duncan L.R., Wang W., Sader H.S. (2020). In Vitro Activity and Potency of the Novel Oxazolidinone Contezolid (MRX-I) Tested against Gram-Positive Clinical Isolates from the United States and Europe. Antimicrob. Agents Chemother..

[376] Guo Q., Xu L., Tan F., Zhang Y., Fan J., Wang X., Zhang Z., Li B., Chu H. (2021). A Novel Oxazolidinone, Contezolid (MRX-I), Expresses Anti-Mycobacterium abscessus Activity In Vitro. Antimicrob. Agents Chemother..

[377] Zhao X., Huang H., Yuan H., Yuan Z., Zhang Y. (2022). A Phase III multicentre, randomized, double-blind trial to evaluate the efficacy and safety of oral contezolid versus linezolid in adults with complicated skin and soft tissue infections. J. Antimicrob. Chemother..

[378] Almeida D., Li S.Y., Lee J., Hafkin B., Mdluli K., Fotouhi N., Nuermberger E.L. (2023). Contezolid can replace linezolid in a novel combination with bedaquiline and pretomanid in a murine model of tuberculosis. Antimicrob. Agents Chemother..

[379] Wu H., Cheng Z., Ding F., Hua J., Wang H., Meng X., Cao Y., Chen Y., Chen Y., Guo B. (2026). Evaluation of the clinical efficacy, safety, and permeability in pulmonary epithelial lining fluid of contezolid, a novel oxazolidinone drug, in adult patients with pneumonia. Antimicrob. Agents Chemother..

[380] Eckburg P.B., Ge Y., Hafkin B. (2017). Single- and Multiple-Dose Study To Determine the Safety, Tolerability, Pharmacokinetics, and Food Effect of Oral MRX-I versus Linezolid in Healthy Adult Subjects. Antimicrob. Agents Chemother..

[381] Barbachyn M.R., Hutchinson D.K., Brickner S.J., Cynamon M.H., Kilburn J.O., Klemens S.P., Glickman S.E., Grega K.C., Hendges S.K., Toops D.S. (1996). Identification of a novel oxazolidinone (U-100480) with potent antimycobacterial activity. J. Med. Chem..

[382] Cynamon M.H., Klemens S.P., Sharpe C.A., Chase S. (1999). Activities of several novel oxazolidinones against *Mycobacterium tuberculosis* in a murine model. Antimicrob. Agents Chemother..

[383] Williams K.N., Stover C.K., Zhu T., Tasneen R., Tyagi S., Grosset J.H., Nuermberger E. (2009). Promising antituberculosis activity of the oxazolidinone PNU-100480 relative to that of linezolid in a murine model. Antimicrob. Agents Chemother..

[384] Zhang M., Sala C., Dhar N., Vocat A., Sambandamurthy V.K., Sharma S., Marriner G., Balasubramanian V., Cole S.T. (2014). In vitro and in vivo activities of three oxazolidinones against nonreplicating *Mycobacterium tuberculosis*. Antimicrob. Agents Chemother..

[385] Heinrich N., Manyama C., Koele S.E., Mpagama S., Mhimbira F., Sebe M., Wallis R.S., Ntinginya N., Liyoyo A., Huglin B. (2025). Sutezolid in combination with bedaquiline, delamanid, and moxifloxacin for pulmonary tuberculosis (PanACEA-SUDOCU-01): A prospective, open-label, randomised, phase 2b dose-finding trial. Lancet Infect. Dis..

[386] Williams K.N., Brickner S.J., Stover C.K., Zhu T., Ogden A., Tasneen R., Tyagi S., Grosset J.H., Nuermberger E.L. (2009). Addition of PNU-100480 to first-line drugs shortens the time needed to cure murine tuberculosis. Am. J. Respir. Crit. Care Med..

[387] Almeida D.V., Omansen T.F., Li S.Y., Lee J., Grosset J.H., Converse P.J., Nuermberger E.L. (2019). Oxazolidinones Can Replace Clarithromycin in Combination with Rifampin in a Mouse Model of Buruli Ulcer. Antimicrob. Agents Chemother..

[388] Wallis R.S., Jakubiec W., Kumar V., Bedarida G., Silvia A., Paige D., Zhu T., Mitton-Fry M., Ladutko L., Campbell S. (2011). Biomarker-assisted dose selection for safety and efficacy in early development of PNU-100480 for tuberculosis. Antimicrob. Agents Chemother..

[389] Mudzengi D., Mashatole S., Xiang Q., Adrion C., Glover N., Ngwanto T., Mdluli J., Boniface S., Rassool M., Matome B. (2026). panTB-HM: A multi-arm clinical trial of a pan-TB regimen targeting both host and microbe. Trials.

[390] Stover C.K., Warrener P., VanDevanter D.R., Sherman D.R., Arain T.M., Langhorne M.H., Anderson S.W., Towell J.A., Yuan Y., McMurray D.N. (2000). A small-molecule nitroimidazopyran drug candidate for the treatment of tuberculosis. Nature.

[391] Tyagi S., Nuermberger E., Yoshimatsu T., Williams K., Rosenthal I., Lounis N., Bishai W., Grosset J. (2005). Bactericidal activity of the nitroimidazopyran PA-824 in a murine model of tuberculosis. Antimicrob. Agents Chemother..

[392] Singh R., Manjunatha U., Boshoff H.I., Ha Y.H., Niyomrattanakit P., Ledwidge R., Dowd C.S., Lee I.Y., Kim P., Zhang L. (2008). PA-824 kills nonreplicating *Mycobacterium tuberculosis* by intracellular NO release. Science.

[393] Tasneen R., Li S.Y., Peloquin C.A., Taylor D., Williams K.N., Andries K., Mdluli K.E., Nuermberger E.L. (2011). Sterilizing activity of novel TMC207- and PA-824-containing regimens in a murine model of tuberculosis. Antimicrob. Agents Chemother..

[394] Diacon A.H., Dawson R., du Bois J., Narunsky K., Venter A., Donald P.R., van Niekerk C., Erondu N., Ginsberg A.M., Becker P. (2012). Phase II dose-ranging trial of the early bactericidal activity of PA-824. Antimicrob. Agents Chemother..

[395] Dutta N.K., Karakousis P.C. (2014). PA-824 is as effective as isoniazid against latent tuberculosis infection in C3HeB/FeJ mice. Int. J. Antimicrob. Agents.

[396] Haver H.L., Chua A., Ghode P., Lakshminarayana S.B., Singhal A., Mathema B., Wintjens R., Bifani P. (2015). Mutations in genes for the F420 biosynthetic pathway and a nitroreductase enzyme are the primary resistance determinants in spontaneous in vitro-selected PA-824-resistant mutants of *Mycobacterium tuberculosis*. Antimicrob. Agents Chemother..

[397] Tasneen R., Betoudji F., Tyagi S., Li S.Y., Williams K., Converse P.J., Dartois V., Yang T., Mendel C.M., Mdluli K.E. (2016). Contribution of Oxazolidinones to the Efficacy of Novel Regimens Containing Bedaquiline and Pretomanid in a Mouse Model of Tuberculosis. Antimicrob. Agents Chemother..

[398] Xu J., Li S.Y., Almeida D.V., Tasneen R., Barnes-Boyle K., Converse P.J., Upton A.M., Mdluli K., Fotouhi N., Nuermberger E.L. (2019). Contribution of Pretomanid to Novel Regimens Containing Bedaquiline with either Linezolid or Moxifloxacin and Pyrazinamide in Murine Models of Tuberculosis. Antimicrob. Agents Chemother..

[399] Conradie F., Diacon A.H., Ngubane N., Howell P., Everitt D., Crook A.M., Mendel C.M., Egizi E., Moreira J., Timm J. (2020). Treatment of Highly Drug-Resistant Pulmonary Tuberculosis. N. Engl. J. Med..

[400] Nyang’wa B.T., Berry C., Kazounis E., Motta I., Parpieva N., Tigay Z., Solodovnikova V., Liverko I., Moodliar R., Dodd M. (2022). A 24-Week, All-Oral Regimen for Rifampin-Resistant Tuberculosis. N. Engl. J. Med..

[401] Abrahams K.A., Batt S.M., Gurcha S.S., Veerapen N., Bashiri G., Besra G.S. (2023). DprE2 is a molecular target of the anti-tubercular nitroimidazole compounds pretomanid and delamanid. Nat. Commun..

[402] Nyang’wa B.T., Berry C., Kazounis E., Motta I., Parpieva N., Tigay Z., Moodliar R., Dodd M., Solodovnikova V., Liverko I. (2024). Short oral regimens for pulmonary rifampicin-resistant tuberculosis (TB-PRACTECAL): An open-label, randomised, controlled, phase 2B-3, multi-arm, multicentre, non-inferiority trial. Lancet Respir. Med..

[403] Cevik M., Thompson L.C., Upton C., Rolla V.C., Malahleha M., Mmbaga B., Ngubane N., Abu Bakar Z., Rassool M., Variava E. (2024). Bedaquiline-pretomanid-moxifloxacin-pyrazinamide for drug-sensitive and drug-resistant pulmonary tuberculosis treatment: A phase 2c, open-label, multicentre, partially randomised controlled trial. Lancet Infect. Dis..

[404] Rahman N.A., Singh S., Wiggins T., Santos M.D., Moraski G.C., Miller M.J., Berney M., Pethe K. (2026). A bactericidal tuberculosis drug regimen driven by inhibition of the terminal oxidases by pretomanid. EMBO Mol. Med..

[405] Cummings J.E., Woolhiser L.K., Guglielmi V., Romano A., Pauly S., Zimmerman M., Kaya F., Walter N.D., Robertson G.T., Slayden R.A. (2026). Pretomanid vs delamanid in a bedaquiline-linezolid regimen: Efficacy in a high-burden tuberculosis mouse model. Antimicrob. Agents Chemother..

[406] Diacon A.H., Dawson R., von Groote-Bidlingmaier F., Symons G., Venter A., Donald P.R., van Niekerk C., Everitt D., Winter H., Becker P. (2012). 14-day bactericidal activity of PA-824, bedaquiline, pyrazinamide, and moxifloxacin combinations: A randomised trial. Lancet.

[407] Matsumoto M., Hashizume H., Tomishige T., Kawasaki M., Tsubouchi H., Sasaki H., Shimokawa Y., Komatsu M. (2006). OPC-67683, a nitro-dihydro-imidazooxazole derivative with promising action against tuberculosis in vitro and in mice. PLoS Med..

[408] Saliu O.Y., Crismale C., Schwander S.K., Wallis R.S. (2007). Bactericidal activity of OPC-67683 against drug-tolerant *Mycobacterium tuberculosis*. J. Antimicrob. Chemother..

[409] Gler M.T., Skripconoka V., Sanchez-Garavito E., Xiao H., Cabrera-Rivero J.L., Vargas-Vasquez D.E., Gao M., Awad M., Park S.K., Shim T.S. (2012). Delamanid for multidrug-resistant pulmonary tuberculosis. N. Engl. J. Med..

[410] Chen X., Hashizume H., Tomishige T., Nakamura I., Matsuba M., Fujiwara M., Kitamoto R., Hanaki E., Ohba Y., Matsumoto M. (2017). Delamanid Kills Dormant Mycobacteria In Vitro and in a Guinea Pig Model of Tuberculosis. Antimicrob. Agents Chemother..

[411] Tucker E.W., Pieterse L., Zimmerman M.D., Udwadia Z.F., Peloquin C.A., Gler M.T., Ganatra S., Tornheim J.A., Chawla P., Caoili J.C. (2019). Delamanid Central Nervous System Pharmacokinetics in Tuberculous Meningitis in Rabbits and Humans. Antimicrob. Agents Chemother..

[412] Hayashi M., Nishiyama A., Kitamoto R., Tateishi Y., Osada-Oka M., Nishiuchi Y., Kaboso S.A., Chen X., Fujiwara M., Inoue Y. (2020). Adduct Formation of Delamanid with NAD in Mycobacteria. Antimicrob. Agents Chemother..

[413] Rifat D., Li S.Y., Ioerger T., Shah K., Lanoix J.P., Lee J., Bashiri G., Sacchettini J., Nuermberger E. (2020). Mutations in fbiD (Rv2983) as a Novel Determinant of Resistance to Pretomanid and Delamanid in *Mycobacterium tuberculosis*. Antimicrob. Agents Chemother..

[414] Pieterman E.D., Keutzer L., van der Meijden A., van den Berg S., Wang H., Zimmerman M.D., Simonsson U.S.H., Bax H.I., de Steenwinkel J.E.M. (2021). Superior Efficacy of a Bedaquiline, Delamanid, and Linezolid Combination Regimen in a Mouse Tuberculosis Model. J. Infect. Dis..

[415] Padmapriyadarsini C., Vohra V., Bhatnagar A., Solanki R., Sridhar R., Anande L., Muthuvijaylakshmi M., Rana M.B., Jeyadeepa B., Taneja G. (2022). Bedaquiline, Delamanid, Linezolid, and Clofazimine for Treatment of Pre-extensively Drug-Resistant Tuberculosis. Clin. Infect. Dis..

[416] Liu Y., Shi J., Li L., Wu T., Chu P., Pang Y., Guo Y., Gao M., Lu J. (2022). Spontaneous Mutational Patterns and Novel Mutations for Delamanid Resistance in *Mycobacterium tuberculosis*. Antimicrob. Agents Chemother..

[417] Seung K.J., Khan P., Franke M.F., Ahmed S., Aiylchiev S., Alam M., Putri F.A., Bastard M., Docteur W., Gottlieb G. (2020). Culture Conversion at 6 Months in Patients Receiving Delamanid-containing Regimens for the Treatment of Multidrug-resistant Tuberculosis. Clin. Infect. Dis..

[418] Franke M.F., Khan P., Hewison C., Khan U., Huerga H., Seung K.J., Rich M.L., Zarli K., Samieva N., Oyewusi L. (2021). Culture Conversion in Patients Treated with Bedaquiline and/or Delamanid. A Prospective Multicountry Study. Am. J. Respir. Crit. Care Med..

[419] Hewison C., Khan U., Bastard M., Lachenal N., Coutisson S., Osso E., Ahmed S., Khan P., Franke M.F., Rich M.L. (2022). Safety of Treatment Regimens Containing Bedaquiline and Delamanid in the endTB Cohort. Clin. Infect. Dis..

[420] Tasneen R., Garcia A., Converse P.J., Zimmerman M.D., Dartois V., Kurbatova E., Vernon A.A., Carr W., Stout J.E., Dooley K.E. (2022). Novel Regimens of Bedaquiline-Pyrazinamide Combined with Moxifloxacin, Rifabutin, Delamanid and/or OPC-167832 in Murine Tuberculosis Models. Antimicrob. Agents Chemother..

[421] Erdem R., Ambler G., Al-Ibrahim M., Fraczek K., Dong S.D., Gast C., Mercer L.D., Raine M., Tennant S.M., Chen W.H. (2021). A Phase 2a randomized, single-center, double-blind, placebo-controlled study to evaluate the safety and preliminary efficacy of oral iOWH032 against cholera diarrhea in a controlled human infection model. PLoS Negl. Trop. Dis..

[422] Kokai-Kun J.F., Roberts T., Coughlin O., Le C., Whalen H., Stevenson R., Wacher V.J., Sliman J. (2019). Use of ribaxamase (SYN-004), a β-lactamase, to prevent Clostridium difficile infection in β-lactam-treated patients: A double-blind, phase 2b, randomised placebo-controlled trial. Lancet Infect. Dis..

[423] Kaleko M., Bristol J.A., Hubert S., Parsley T., Widmer G., Tzipori S., Subramanian P., Hasan N., Koski P., Kokai-Kun J. (2016). Development of SYN-004, an oral beta-lactamase treatment to protect the gut microbiome from antibiotic-mediated damage and prevent Clostridium difficile infection. Anaerobe.

[424] Kokai-Kun J.F., Roberts T., Coughlin O., Sicard E., Rufiange M., Fedorak R., Carter C., Adams M.H., Longstreth J., Whalen H. (2017). The Oral β-Lactamase SYN-004 (Ribaxamase) Degrades Ceftriaxone Excreted into the Intestine in Phase 2a Clinical Studies. Antimicrob. Agents Chemother..

[425] Torres M.D.T., Wan F., de la Fuente-Nunez C. (2025). Deep learning reveals antibiotics in the archaeal proteome. Nat. Microbiol..

